# Inhaled LTI-03 for idiopathic pulmonary fibrosis: a randomized dose escalation study

**DOI:** 10.1038/s41467-026-75291-3

**Published:** 2026-07-31

**Authors:** Philip L. Molyneaux, Nikhil A. Hirani, Collin C. K. Chia, Tejaswini Kulkarni, Tanzira Zaman, Robert J. Kaner, Ana Lucia Coelho, Yago Amigo Pinho Jannini-Sa, Brian Windsor, Sydney Kruger, Dale J. Christensen, Steven A. Shoemaker, Cory M. Hogaboam, BreAnne MacKenzie, Andreas Günther

**Affiliations:** 1 National Heart and Lung Institute, Imperial College London, London, UK; 2 Institute for Regeneration and Repair, University of Edinburgh, Edinburgh, UK; 3 Launceston Respiratory and Sleep Center, Launceston General Hospital, Launceston, TAS Australia; 4 Pulmonary, Allergy & Critical Care Medicine, University of Alabama at Birmingham, Birmingham, AL USA; 5 Pulmonary & Critical Care Medicine, Cedars Sinai Medical Center, Los Angeles, CA USA; 6 Weill Cornell Medicine, New York, NY USA; 7 Women’s Guild Lung Institute, Cedars Sinai Medical Center, Los Angeles, CA USA; 8 Rein Therapeutics, Inc., Austin, TX USA; 9 Department of Medicine, Duke University School of Medicine, Durham, NC USA; 10 Center for Interstitial and Rare Lung Diseases, Justus-Liebig-University, University of Giessen and Marburg Lung Center, Member of the German Center for Lung Research, Giessen, Germany

**Keywords:** Biomarkers, Respiratory tract diseases, Respiratory signs and symptoms

## Abstract

Idiopathic pulmonary fibrosis (IPF) is a fatal interstitial lung disease with limited treatment options. LTI-03 promotes alveolar epithelial cell survival and reduces profibrotic protein expression in experimental models of IPF. In this Phase 1b, randomized, double-blind, placebo-controlled dose-escalation study, 24 participants with IPF were randomized 3:1 to inhaled LTI-03 5 mg/day (N = 9), LTI-03 10 mg/day (N = 9) or placebo (N = 6) for 14 days and included in all analyses (ClinicalTrials.gov: NCT05954988). The primary endpoint was the incidence of treatment-emergent adverse events (TEAEs). Exploratory analyses included pharmacokinetics and disease-related biomarkers. LTI-03 was well-tolerated, with no treatment-related discontinuations, no severe TEAEs, and no evidence of airway obstruction by spirometry and associated symptoms. In deep bronchial brushings, both LTI-03 doses significantly reduced interleukin-11 (p = 0.0406 at 5 mg/day; p = 0.044 at 10 mg/day) and thymic stromal lymphopoietin (p = 0.0256 at 5 mg/day; p = 0.0128 at 10 mg/day) versus placebo. The 10 mg/day dose suppressed collagen type 1 alpha chain 1 (p = 0.0248), CXC chemokine ligand 7 (p = 0.0248) and galectin-7 (p = 0.0332). Other measured biomarkers were not significantly changed. The favorable safety profile and reductions in disease-related biomarkers support further evaluation of inhaled LTI-03 for IPF. This study was fully funded by Rein Therapeutics, Inc.

## Abstract (web summary)

Idiopathic pulmonary fibrosis (IPF) is a progressive and often fatal scarring disease of the lungs with very limited treatment options. Here, the authors show that in a randomized placebo-controlled Phase 1b clinical trial in IPF patients, inhaled LTI-03 was safe and well tolerated. Exploratory analyses showed a reduction in several key proteins driving lung fibrosis.

## Introduction

Idiopathic pulmonary fibrosis (IPF) is a progressive, fatal, age-associated lung disease with poor survival^1^. The pathogenesis of IPF is characterized by apoptosis and senescence of alveolar epithelial type 2 cells, proliferation and accumulation of activated myofibroblasts, deposition of extracellular matrix (ECM), and fibrosis, resulting in progressive dyspnea and loss of lung function, especially forced vital capacity (FVC)^2,3^. The approved anti-fibrotic drugs nintedanib (Ofev^®^), pirfenidone (Esbriet^®^), and nerandomilast (Jascayd^®^) slow the rate of FVC decline^2,4^, but do not halt disease progression or restore lung function. Additionally, nintedanib and pirfenidone may be poorly tolerated due to gastrointestinal or cutaneous side effects, leading to frequent discontinuation^5,6^. Hence, there is an urgent need for therapies better targeting the underlying causes of IPF and other interstitial lung diseases; in particular, alveolar protective treatments that may act to restore healthy lung function with a more favorable tolerability profile.

Caveolin-1 protein (Cav-1) is a structural protein that plays a critical role in regulating lung repair and cellular movement by restoring pathways that both promote epithelial health and halt fibrosis in the lung^7–9^. Cav-1 expression in pulmonary fibroblasts and alveolar epithelial cells is decreased in transgenic and rodent bleomycin models of lung injury and in the lungs of patients with IPF^10–12^. Augmentation of endogenous Cav-1 levels and supplementation of exogenous caveolin scaffolding domain (CSD) peptide have been shown to suppress lung fibrosis^7,10,11,13,14^. LTI-03 is a synthetic oligopeptide consisting of seven natural L-amino acids of the CSD. LTI-03 has been shown to exert strong antifibrotic and epithelial supportive effects in experimental animal models of lung fibrosis^15–17^, in human IPF precision cut lung slices^18^, and in human IPF alveolar organoids^19^.

While LTI-03 first demonstrated efficacy upon intraperitoneal administration in mouse models of IPF^20^, its limited aqueous solubility necessitated an alternate formulation and delivery strategy. Micronized, excipient-free LTI-03 demonstrated excellent stability, and importantly, the ability to achieve deep lung deposition^21^. LTI-03 (2.5 mg/capsule) is self-administered by study participants using a commercially available dry-powder inhaler.

Here we report the safety and tolerability of LTI-03 following twice-daily (BID) inhaled administration (total doses of 5 mg/day or 10 mg/day) to participants with IPF for 14 days. Finally, LTI-03 treatment-related impacts on exploratory biomarkers, which were selected based on nonclinical, translational experimental results, are presented and discussed.

## Results

### Participant disposition and characteristics

**Fig. 1:**
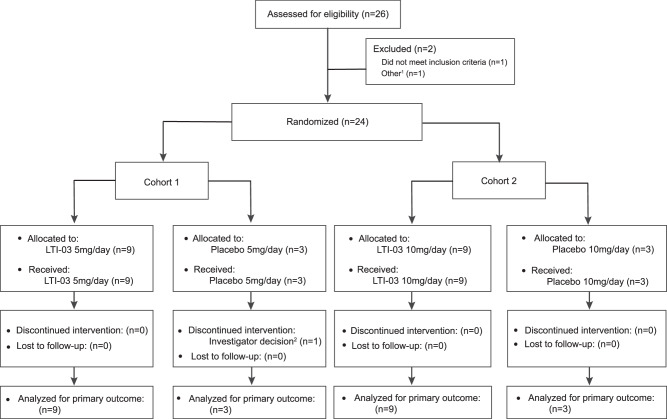
Phase 1b clinical study participant disposition. Notes: ^1^Participant screen failed due to a lack of study drug on-site. ^2^LTI-03 dosing incorrectly stopped by the Investigator on Day 14 due to “met stopping criteria with decrease in forced expiratory volume in 1 s (FEV_1_)”; however, the protocol stopping criteria also required an associated decrease from baseline in the FEV_1_/FVC ratio, which was not met and would have supported continued dose administration. The participant completed the study.

Twenty-four participants with IPF met eligibility criteria and were enrolled between June 2023 and October 2024 into 2 sequential dose cohorts: Cohort 1 (5 mg/day LTI-03 versus placebo), followed by Cohort 2 (LTI-03 10 mg/day versus placebo) (Fig. 1). Within each cohort, participants were randomized 3:1 to study drug (LTI-03:placebo), which was self-administered BID for 14 days. No participant discontinued study treatment due to a treatment-emergent adverse event (TEAE), and all completed the study. LTI−03 dosing was incorrectly stopped by the Investigator on Day 14 for 1 participant in the LTI-03 5 mg/day group due to “met stopping criteria with decrease in forced expiratory volume in 1 s (FEV_1_)”; however, the protocol stopping criteria also required an associated decrease from baseline in the FEV_1_/FVC ratio, which was not met and would have supported continued dose administration. At baseline, demographics and disease characteristics were generally well balanced across the treatment groups (Table 1). None of the participants reported use of any approved or investigational antifibrotic drugs within 2 months of screening, or during the study.

**Table 1:** Baseline Demographics and Disease Characteristics

Parameter	LTI-03 5 mg/day (*N* = 9)	LTI-03 10 mg/day (*N* = 9)	LTI-03 Pooled (*N *= 18)	Placebo Pooled (*N* = 6)
Age, yr, mean (SD)	67.8 (9.76)	68.4 (10.79)	68.1 (9.99)	71.7 (6.15)
Male, *n* (%)	7 (77.8)	7 (77.8)	14 (77.8)	6 (100)
Female, *n* (%)	2 (22.2)	2 (22.2)	4 (22.2)	0
Race and Ethnicity, *n* (%)				
White, Not Hispanic or Latino	9 (100)	9 (100)	18 (100)	6 (100)
BMI (kg/m^2^), mean (SD)	28.9 (2.73)	29.4 (6.63)	29.1 (4.93)	29.3 (4.99)
Past/current cigarette use, n (%)	8 (88.9)	7 (77.8)	15 (83.3)	4 (66.7)
FVC percent predicted, mean (SD)	84.8 (10.22)	92.1 (18.66)	88.5 (15.08)	75.6 (8.94)
DLCO percent predicted, mean (SD)	59.0 (7.66)*	60.9 (11.70)	60.0 (9.75)^†^	61.4 (10.11)
LCQ total score, mean (SD)	18.2 (3.26)	16.6 (3.24)	17.4 (3.26)	16.1 (3.37)
Cough VAS score, mean (SD)	29.6 (30.65)	38.1 (21.51)	33.8 (26.06)	35.3 (27.20)

SD, standard deviation; BMI, body mass index; DLCO, diffusion capacity of the lungs for carbon monoxide; FVC, forced vital capacity; LCQ, Leicester Cough Questionnaire; VAS, Visual Analog Scale. Baseline was defined as the last non-missing measurement prior to the first dose of study drug.

^*^Data available for *n* = 8.

^†^Data available for *n* = 17.

### Safety and tolerability

LTI-03 was well tolerated over 14 days of BID dosing at total doses of 5 mg/day and 10 mg/day. The incidence of TEAEs was 66.7% (LTI-03 5 mg/day), 77.8% (LTI-03 10 mg/day), and 50% (placebo), respectively (Table 2). There were no fatalities or TEAEs leading to treatment discontinuation. One (4.2%) participant in the LTI-03 10 mg/day group experienced an unrelated serious TEAE of Grade 2 post-bronchoscopy fever requiring overnight inpatient observation. Most TEAEs in all dose groups were Grade 1 in severity. Grade 2 TEAEs were reported by 3 participants (16.7%) receiving LTI-03, including 2 cases of cough and 1 case of post-procedural fever; no TEAEs in any group were Grade 3 or higher. All TEAEs are summarized in Supplementary Table 1.

**Table 2:** Overview of Treatment-Emergent Adverse Events

Event, *n* (%)	LTI-03 5 mg/day (*N* = 9)	LTI-03 10 mg/day (*N* = 9)	LTI-03 Pooled (*N* = 18)	Placebo Pooled (*N* = 6)
Any TEAE	6 (66.7)	7 (77.8)	13 (72.2)	3 (50.0)
TEAEs leading to treatment discontinuation	0	0	0	0
Serious TEAEs	0	1 (11.1)	1 (5.6)	0
TEAEs related to the study drug	2 (22.2)	5 (55.6)	7 (38.9)	1 (16.7)
TEAEs Grade 1	5 (55.6)	5 (55.6)	10 (55.6)	3 (50)
Grade 2	1 (11.1)	2 (22.2)	3 (16.7)	0
≥ Grade 3	0	0	0	0
TEAEs in ≥2 participants				
Cough	3 (33.3)	5 (55.6)	8 (44.4)	2 (33.3)
Rhinorrhea	1 (11.1)	1 (11.1)	2 (11.1)	0

TEAE, treatment-related adverse event: an AE which began or increased in severity or frequency at or after the administration of the first study drug. Adverse events were graded following the Common Terminology Criteria for Adverse Events, Version 5, including Grade 1 (mild), Grade 2 (moderate), Grade 3 (severe), Grade 4 (life-threatening), and Grade 5 (fatal). The summary of all TEAEs is provided in Supplemental Table 1.

Cough was the most common TEAE, reported by 3 (33.3%; LTI-03 5 mg/day), 5 (55.6%; LTI-03 10 mg/day), and 2 (33.3%; placebo) participants, respectively. Rhinorrhea, reported by 1 participant in each of the LTI-03 treatment groups, was the only other TEAE reported by more than 1 participant in any group. A Grade 2 cough related to the study drug that resolved on the same day was reported in 1 (11.1%) participant each in the LTI-03 5 mg/day and LTI-03 10 mg/day groups, respectively. Cough was the only study drug-related TEAE experienced by more than 1 participant. No symptoms of airway obstruction such as chest tightness, dyspnea, or wheezing were reported (Supplementary Table 1). None of the study drug-related TEAEs was a serious TEAE or led to treatment discontinuation.

No apparent treatment- or dose-related trends in clinical laboratory parameters, vital sign measurements, 12-lead electrocardiogram (ECG) results, or physical examination findings were observed during the study. Similarly, mean changes from baseline in FEV_1_ and FVC measured by spirometry at each clinic visit (Table 3) were not clinically significant, indicating no meaningful effects on lung function following 14 days of study drug inhalation. Consistent with these findings, individual participant data showed no notable trends (Supplementary Fig. 1).

**Table 3:** Lung Function Measurements Over Time

	LTI-03 5.0 mg/day (*N* = 9)		LTI-03 10 mg/day (*N* = 9)		Placebo Pooled (*n *= 6)	
FEV_1_ (L)	Absolute	Change from Baseline	Absolute	Change from Baseline	Absolute	Change from Baseline
Baseline	2.70 (0.76)	—	2.59 (0.55)	—	2.46 (0.25)	—
Day 7	2.75 (0.73)	0.05 (0.13)	2.61 (0.49)*	−0.08 (0.21)	2.53 (0.34)	0.07 (0.22)
Day 14	2.64 (0.78)*	−0.04 (0.30)	2.48 (0.55)	−0.11 (0.20)	2.58 (0.23)	0.13 (0.25)
Follow-up	2.75 (0.81)	0.05 (0.16)	2.54 (0.53)	−0.05 (0.12)	2.59 (0.26)	0.13 (0.19)
**FVC (L)**	**Absolute**	**Change from Baseline**	**Absolute**	**Change from Baseline**	**Absolute**	**Change from Baseline**
Baseline	3.23 (0.83)	—	3.28 (0.64)	—	3.08 (0.26)	—
Day 7	3.25 (0.80)	0.30 (0.01)	3.41 (0.47)*	−0.02 (0.19)	3.23 (0.55)	0.15 (0.41)
Day 14	3.17 (0.93)*	−0.01 (0.35)	3.22 (0.59)	−0.07 (0.20)	3.35 (0.55)	0.28 (0.46)
Follow-up	3.34 (0.92)	−0.12 (0.19)	3.29 (0.61)	0.00 (0.13)	3.31 (0.51)	0.24 (0.38)

FEV_1_, Forced Expiratory Volume in 1 s; FVC, Forced Vital Capacity. Change from baseline to end of treatment (Day 14) and post-treatment follow-up (Day 21). Values presented as mean ± standard deviation (SD). Baseline spirometry values are calculated as averages of Screening and Day 1 pre-dose values. Change from baseline is calculated as post-baseline value minus baseline value. *Data available for *n* = 8.

Participants self-reported cough symptoms using the Leicester Cough Questionnaire (LCQ)^22^ and a cough severity visual analogue scale (VAS). Baseline results are summarized in Table 1. No clinically significant changes in the LCQ total score or the cough VAS were observed during the treatment period for any treatment group, indicating that inhalation of the study drug for 14 days did not appear to negatively affect participants’ quality of life related to cough or worsen pre-existing chronic cough symptoms.

### Pharmacokinetics

All post-dose plasma concentrations following LTI-03 inhalation were below the limit of quantification (BLQ) on Days 1, 7, and 14. LTI-03 concentrations in bronchoalveolar lavage fluid (BALF) at Day 14 were quantifiable in 3 participants in the LTI-03 5 mg/day group (1.55, 6.60, and 1.37 ng/mL) and 2 participants in the LTI-03 10 mg/day group (2.57 and 14.7 ng/mL); all other BALF sample concentrations were BLQ.

### Exploratory Biomarkers

Our exploratory biomarker strategy was based on the pathobiology of Cav-1 in the context of fibrosis^7,23–26^; IPF pathogenesis^27,28^, and importantly, pharmacodynamic data for LTI-03 from a variety of translational models^17–19^. Based on these data, it was hypothesized that LTI-03 treatment would cause a reduction of these biomarkers in a clinical setting. Plasma levels of surfactant protein D (SP-D), an indicator of epithelial cell stress, trended downwards and was decreased by 5% in the LTI-03 10 mg/day group compared to placebo. LTI-03 treatment did not change phosphorylated (p) AKT over total AKT levels in peripheral blood mononuclear cells (PBMCs). In deep bronchial brushing (DBB) samples, LTI-03 treatment significantly reduced expression of multiple profibrotic proteins compared to placebo, including collagen type 1 alpha chain 1 (COL1A1), chemokine (C-X-C motif) ligand 7 (CXCL7), galectin-7 (GAL-7), interleukin 11 (IL-11), and thymic stromal lymphopoietin (TSLP) (Table 4; Supplementary Fig. 2).

**Table 4:** Exploratory Biomarkers Change from Baseline to Day 14

Analyte	Treatment Group	N	Δ from Baseline median (IQR)	U Statistic	p-value (one-tailed Mann-Whitney)	Median Difference (Hodges-Lehmann)	90% CI for Median Difference	Cliff’s delta
**PLASMA**								
SP-D (pg/mL)	Placebo	5	2760 (−8442 to 10644)	-	-	-	-	-
	LTI-03 5 mg/day	8	3290 (−2235 to 18476)	16	0.3108	3428	−9397 to 20313	0.20
	LTI-03 10 mg/day	8	−3264 (−29238 to 1299)	11	0.1111	−8368	−34056 to 2168	0.45
**PERIPHERAL BLOOD MONONUCLEAR CELLS**								
%p-AKT (%pAKT/total AKT)	Placebo	6	−4.15 (−24.61 to 19.55)	-	-	-	-	-
	LTI-03 5 mg/day	9	2.67 (−2.013 to 16.32)	18	0.1638	6.818	−17.14 to 39.48	−0.33
	LTI-03 10 mg/day	9	−2.65 (−7.972 to 1.948)	23.5	0.3584	0.9608	−15.35 to 11.93	−0.13
**DEEP BRONCHIAL BRUSHINGS**								
COL1A1 (pg/mg protein)	Placebo	6	4.74 (−13.29 to 31.24)	-	-	-	-	-
	LTI-03 5 mg/day	8	0.16 (−1.169 to 2.486)	12	0.0709	−3.486	−21.63 to 1.507	0.50
	LTI-03 10 mg/day	9	−11.9 (−52.80 to −1.265)	10	0.0248	−20.95	−66.84 to −3.058	0.72
CXCL7 (ng/mg protein)	Placebo	6	−140.7 (−589.8 to 3143.0)	-	-	-	-	-
	LTI-03 5 mg/day	8	−100.2 (−198.6 to 83.1)	24	0.5	8.265	−2667 to 470.2	0.00
	LTI-03 10 mg/day	8	−1276.23 (−1611 to −60.56)	10	0.0248	−1262	−3763 to −147.7	0.58
GAL7 (pg/mg protein)	Placebo	6	6010.4385 (−1746 to 7369)	-	-	-	-	-
	LTI-03 5 mg/day	8	250.8575 (−2526 to 2159)	12	0.0709	−4730	−8196 to 1235	0.50
	LTI-03 10 mg/day	9	−726.395 (−2660 to 810.1)	11	0.0332	−6048	−8754 to −478.0	0.59
IL-11 (pg/mg protein)	Placebo	6	10.6765 (−12.13 to 38.33)	-	-	-	-	-
	LTI-03 5 mg/day	8	−11.037 (−15.70 to 3.427)	10	0.0406	−21.71	−68.30 to −2.159	0.58
	LTI-03 10 mg/day	9	−6.528 (−20.45 to −1.258)	12	0.044	−19.15	−59.36 to −2.040	0.56
TSLP (pg/mg protein)	Placebo	6	14.7125 (0.3573 to 23.15)	-	-	-	-	-
	LTI-03 5 mg/day	7	−2.106 (−11.05 to 1.145)	7	0.0256	−19.66	−27.06 to −2.780	0.67
	LTI-03 10 mg/day	9	−2.09 (−12.75 to 2.656)	8	0.0128	−19.07	−26.74 to −3.454	0.70

SP-D, surfactant protein D; COL1A1, collagen 1 type A chain 1; CXCL-7, chemokine (C-X-C motif) ligand 7; GAL-7, galectin-7; IL-11 interleukin 11; TSLP, thymic stromal lymphopoietin. Change from baseline was calculated as Day 14 minus screening. Note: Cliff’s delta is the effect size ( < 0.3 small, 0.3–0.5 medium, >0.5 large), with negative values indicating reductions in treatment vs. placebo. Sample sizes (N) vary due to missing samples or values below the limit of quantification (BLQ; excluded).

## Discussion

LTI-03 is a CSD peptide formulated as an excipient-free dry powder for inhalation. This first-in-patient study evaluated the safety and tolerability of LTI-03 compared to placebo when administered BID for 14 days in 24 participants with IPF. Participants were diagnosed with IPF within 3 years of screening per guidelines; none reported receiving prior treatment with nintedanib, pirfenidone, or nerandomilast within 2 months of screening or during the study. Inhaled LTI-03 at doses of 5 mg/day (2.5 mg BID) and 10 mg/day (5 mg BID) was well tolerated, and all 24 participants completed the study. There were no TEAEs that led to treatment discontinuation. The majority of TEAEs were mild (Grade 1) in severity; only 3 (16.7%) participants reported Grade 2 AEs, and none (0%) reported TEAEs Grade 3 or higher. One participant in the LTI-03 5 mg/day group experienced a serious TEAE of Grade 2 fever following bronchoscopy that warranted overnight admission for observation; they had no clinical signs or symptoms of infection, no antibiotic treatment was required during or after hospital discharge, and the event was considered unrelated to study drug. Post-procedural fever is a known complication following bronchoscopy^29^.

Coughing is a hallmark of IPF and represents one of the key symptoms of IPF patients in larger cohorts^30^. In line with previous reports on inhaled drugs in IPF^31^, cough was also the most frequently reported TEAE for 44% of participants receiving LTI-03, as compared to the 33% receiving placebo, and the only study drug-related TEAE experienced by more than 1 participant. The majority of cough TEAEs were mild in severity except for 2 study drug-related TEAEs of Grade 2 severity, each resolving on the same day: one occurred as a single episode, and the other occurred intermittently. All other study drug-related TEAEs of cough were infrequent and Grade 1 in severity, occurring after study drug administration. Other than rhinorrhea, which was reported for 1 participant each in the LTI-03 5 mg/day and 10 mg/day groups, no other TEAEs were reported by more than 1 participant.

Participants self-reported the effect of LTI-03 on their chronic cough symptoms and daily life using the LCQ^22^, and the severity of their cough symptoms using a visual analogue scale that rated cough severity (0 = no cough to 100 = worst cough ever). Self-reported LCQ and VAS results indicated that inhalation of the study drug did not appear to negatively affect participants’ quality of life or worsen pre-existing chronic cough symptoms.

Spirometry was performed as a safety assessment to evaluate the effects of repeat-dose study drug inhalation on airways. There were no clinically significant differences between the LTI-03 and placebo groups in the mean changes in FEV1 and FVC over the treatment period. Furthermore, there was no evidence of airway obstruction by spirometry and participant-reported symptoms such as chest tightness, non-exertional dyspnea, or wheezing following study drug inhalation.

Systemic levels of LTI-03 measured immediately after LTI-03 inhalation were undetectable in all participants, regardless of LTI-03 dose. Undetectable plasma levels of LTI-03, a hydrophobic peptide, are consistent with the PK behavior of other inhaled hydrophobic compounds, such as inhaled cyclosporine formulations, which exhibit rapid and sustained lung absorption with minimal systemic exposure^32^. Taken together, these data support lung-targeted delivery of LTI-03 with minimal systemic exposure. However, LTI-03 was detected in BALF samples collected after inhalation in 5 participants (3 in the 5 mg/day; 2 in the 10 mg/day group), confirming the distribution of LTI-03 into the lung. In BALF, the measurement of LTI-03 was limited by the low solubility of LTI-03 in aqueous solutions, differences in time between drug inhalation and sample collection via bronchoscopy, as well as variations in the volume of BALF sample collected.

It is hypothesized that IPF pathophysiology is driven by the persistent microinjury of alveolar epithelial cells leading to apoptosis and necrosis, downstream aberrant fibroblast activation, and abnormal accumulation of extracellular matrix^3^. In multiple animal models of fibrosis, LTI-03 has repeatedly demonstrated significant attenuation of profibrotic factors and pathways as well as supportive effects on alveolar epithelial cell maintenance and homeostasis^17,20^. These pharmacological effects were also demonstrated in IPF and donor precision cut lung slices (PCLS)^18^, which contain all diseased lung cell types, as well as in both lung epithelial cell organoid cultures^19^ and fibroblasts sourced from end-stage IPF patients^20^.

Exploratory biomarkers for this study were selected based on previous translational study outcomes, which demonstrated robust, consistent reduction of these markers following LTI-03 treatment either in vitro or in in vivo animal models of IPF. Changes in biomarker levels from baseline to day 14 were assessed in plasma, PBMCs, and bronchoscopy-derived DBB samples. A one-tailed Mann-Whitney U test was selected to test the directional hypotheses of reduced biomarker expression following LTI-03 treatment.

In plasma samples, levels of SP-D, an indicator of epithelial cell health, were decreased by 5% (*p* = 0.1111) for the LTI-03 10 mg/day group compared to placebo. While not statistically significant, these reductions may be clinically relevant, as increased circulating SP-D levels are significantly linked to decline in lung function and are responsive to standard of care^33,34^. Moreover, the 5% drop in SP-D is comparable to decreases described in standard of care IPF drug trials after longer treatment durations, notably the CAPACITY study of pirfenidone (5% at 12 weeks)^35^ and the INMARK study of nintedanib (4% at 12 weeks)^36,37^ which may indicate the potential for LTI-03 to slow the rate of lung function decline. In addition, SP-D is a key analyte in a platform currently in development by the PROLIFIC consortium, which is on track to gain regulatory approval for the creation of a diagnostic, prognostic, and theragnostic assay for IPF patients^38^.

In this study, LTI-03, compared to placebo, did not modulate the per cent phosphorylated AKT over total AKT in PBMCs from patients, suggesting that inhaled administration does not lead to changes in circulating immune cells.

In DBB samples, analyses revealed consistent reductions in key profibrotic and inflammatory markers with LTI-03 compared to placebo, particularly at the 10 mg/day dose level. LTI-03 consistently reduced collagen expression and deposition in animal models and in vitro^17,20^. Levels of COL1A1, a marker of extracellular matrix deposition, decreased significantly with LTI-03 10 mg/day compared to placebo. Similarly, CXCL7, a chemokine involved in inflammatory processes in the lung, was significantly attenuated in DBB samples from participants who received LTI-03 10 mg/day. These results are consistent with prior data demonstrating decreased CXCL7 levels with LTI-03 treatment in IPF PCLS supernatants^18^. CXCL7 levels were elevated in bronchoalveolar lavage fluid (BALF) from patients with IPF compared with healthy controls^39^. While chemokine signaling is increasingly recognized as contributing to IPF pathobiology, the specific role of CXCL7 in disease initiation or progression has not yet been defined^40^.

Galectin-7, a protein associated with epithelial repair and fibrosis^17^ was reduced for both the LTI-03 5 mg/day and 10 mg/day groups compared to placebo. Immunohistochemical analyses of IPF lung tissue have shown intense GAL-7 expression localized to the alveolar and bronchiolar epithelium and alveolar septum^12^. LTI-03-mediated reductions in GAL-7 in DBB samples suggest that inhaled LTI-03 had a targeted effect in the deep lung.

Interleukin-11 levels in DBB samples were significantly reduced in both LTI-03 treatment groups compared to placebo. This is consistent with results from aged male mice challenged with bleomycin, in which BALF levels of IL-11 were reduced with LTI-03 treatment^17^. IL-11 is a key driver of myofibroblast activation and differentiation in IPF^41^, and is currently the focus of an ongoing clinical study in IPF with an investigational monoclonal antibody (ClinicalTrials.gov: NCT07036523).

Robust reductions of TSLP were also observed in both the LTI-03 5 mg/day and 10 mg/day treatment groups compared to placebo. TSLP is an alarmin cytokine that is released following epithelium damage and promotes type 2 inflammation and fibrosis, driving pathological processes such as mucus hypersecretion and airway remodeling^42^. Levels of TSLP are significantly elevated in BALF^43^ and lung tissue from patients with IPF^44^ and immunohistochemical studies have localized elevated TSLP expression to alveolar epithelial cells and fibrotic foci^44^. In IPF, TSLP may promote fibrogenesis by activating fibroblasts and recruiting immune cells to sites of epithelial injury^44^. Statistically significant reductions in TSLP following LTI-03 treatment support the hypothesis that LTI-03 may have positive effects on lung epithelial cell health.

Although the sample size was limited, the reductions in profibrotic biomarkers were supported by medium-to-large effect sizes (Cliff’s delta), reinforcing the biological relevance of these pharmacodynamic signals despite the short 14-day treatment period. The effects suggest LTI-03 attenuates fibrotic pathways and supports epithelial integrity in the lung microenvironment, aligning with LTI-03’s mechanism of action targeting caveolin-1-mediated signaling. Together, these data support the disease-modifying potential of LTI-03, warranting further clinical evaluation with functional endpoints to confirm disease modification in IPF.

There remains an unmet medical need for safe and effective treatments that target epithelial cell injury in IPF. Inhaled therapies can facilitate increased drug delivery to the site of disease while minimizing systemic off-target effects. The overall low incidence of serious, treatment-related, or severe TEAEs, absence of treatment discontinuations due to a TEAE, and no detectable systemic absorption suggest that inhaled LTI-03 has the potential for a more favorable safety, tolerability, and adherence profile compared to the currently approved, systemically administered treatments. Preclinical data in experimental models and evidence of active LTI-03 pharmacodynamics in this study suggest that LTI-03 may regulate processes driving both lung repair and fibrosis.

Limitations in the interpretation of this study include the small sample size (*n* = 24) and a short treatment duration of 14 days. A sample size of 24 participants was planned to ensure that safety and tolerability of LTI-03 were adequately assessed while minimizing unnecessary participant exposure. No conclusion of LTI-03 effects on disease progression can be derived from the data collected. While none of the participants were receiving antifibrotic therapy in this study, combination therapies are emerging as standard of care. Larger studies of longer treatment durations in patients with IPF receiving antifibrotic therapy will be required to confirm and expand upon the findings of this first-in-patient study.

This study showed that the administration of inhaled LTI-03 to participants with IPF was safe and well-tolerated at doses of 5 and 10 mg/day. Additionally, an exploratory analysis of pro-fibrotic, epithelial health, and inflammatory biomarkers suggests a potential pharmacodynamic benefit of LTI-03 administration without effects on circulating immune cells. These encouraging data support further evaluation of inhaled LTI-03 in a Phase 2 study of longer duration in patients with IPF receiving background standard of care (RENEW; ClinicalTrials.gov: NCT06968845).

## Methods

### Study design

This was a Phase 1b, randomized, double-blind, placebo-controlled, dose escalation study conducted at 6 sites in the United States, United Kingdom, Germany, and Australia (ClinicalTrials.gov: NCT05954988). The primary objective was to assess the safety and tolerability of inhaled LTI-03 compared to placebo in patients with IPF. Exploratory objectives were to assess LTI-03 pharmacokinetics (PK) and pharmacodynamics.

**Fig. 2:**
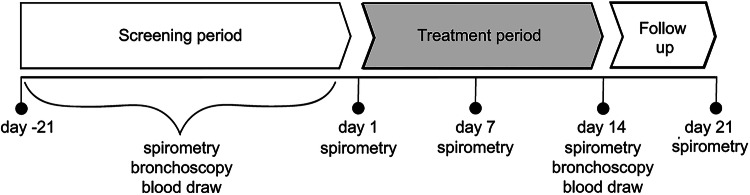
Phase 1b clinical study protocol design. Spirometry, bronchoscopy, and blood biomarker collection timepoints. Blood for biomarker analyses were collected during screening prior to the bronchoscopy.

The study had 3 periods: screening (up to 21 days), treatment (14 days) and follow-up (7 days), with study visits on Days 1, 7, 14, and 21 (Fig. 2). The full study protocol and statistical analysis plan are provided as Supplementary Notes 1 and 2, respectively. The completed CONSORT checklist for reporting of this study is provided as Supplementary Note 3.

### Study population

Participants were recruited beginning on 22 June 2023. The first participant was enrolled on 06 July 2023, and the last participant completed all outcome assessments on 01 October 2024. Participants were compensated for their time and travel expenses according to local IRB/ethics committee-approved guidelines and standard clinical trial practices.

Eligible participants were male or female ≥ 40 years of age, willing and able to provide written informed consent. Participants were required to have a diagnosis of IPF per ATS/ERS/JRS/ALAT guidelines^45^ made within 3 years of enrollment, with FVC percent predicted ≥ 40%, DLCO percent predicted between 30% and 80%, and FEV₁/FVC ≥ 0.7.

Participants with interstitial lung disease other than IPF, significant obstructive lung disease, a current diagnosis of asthma, or who experienced a pulmonary exacerbation within 6 months prior to screening, or a febrile illness within 7 days prior to dosing were excluded; as were those with severe progressive or uncontrolled, clinically significant disease that in the judgment of the investigator or designee rendered the participant unsuitable for the study. Also excluded were participants with significant renal impairment (estimated glomerular filtration rate < 30 mL/min) or hepatic impairment (bilirubin > 3 mg/dL [> 51.3 μmol/L] and albumin <2.8 g/dL [<28 g/L] and PT prolongation > 6 sec or INR > 2.3), and those with a serious or active medical or psychiatric condition which, in the opinion of the Investigator, would interfere with treatment, assessment, or compliance with the protocol.

Participants who were unable to use the study inhaler device appropriately were ineligible. Use of N-acetyl cysteine or other supplements within 7 days prior to dosing and throughout the Treatment Period was not allowed. In addition, participants could not have received treatment with an approved or investigational anti-fibrotic therapy for IPF within 2 months of the baseline bronchoscopy, or a vaccination within 2 weeks of the start of dosing (Day 1) and throughout the Treatment Period. Participation in a clinical study or treatment with an investigational drug or device within 30 days of the Screening Visit (or 5 half-lives of the investigational agent, whichever is longer) was also not allowed. Females of childbearing potential were ineligible if they had a positive pregnancy test or were lactating. Females of childbearing potential and men with partners of childbearing potential who did not agree to use an acceptable form of contraception for the duration of study treatment and for at least 90 days after the last dose of study drug were also excluded, as were male participants who did not agree to refrain from donating sperm during this same period.

The sex of participants was self-reported (as male or female) for demographic purposes after informed consent was obtained. Gender identity was not collected. Sex was not considered in the design of this study due to the small sample size.

### Randomization treatment and blinding

Eligible participants were randomized 3:1 to LTI-03 or placebo into 2 sequential cohorts (*n* = 12 per cohort). The 5 mg/day (low-dose) cohort received 2.5 mg BID of LTI-03 or placebo, and the 10mg/day (high-dose) cohort received 5mg BID of LTI-03 or placebo. Randomization was performed by interactive response system. Participants, study staff, and sponsor personnel were blinded to treatment assignment throughout the study.

The study complied with Good Clinical Practice and the Declaration of Helsinki. The study protocol and informed consent form were approved by the Institutional Review Board or Independent Ethics Committee for each site: South Central – Oxford A Research Ethics Committee for the Royal Brompton and Harefield Hospitals and the Royal Infirmary of Edinburgh (UK); Bellberry Human Research Ethics Committee for Launceston General Hospital (Australia); WCG IRB for University of Alabama at Birmingham (USA); Advarra, Inc. for Cedars-Sinai Medical Center (USA); and the Ethics Committee of the Faculty of Medicine for Justus-Liebig-University Giessen (Germany). All participants provided written informed consent.

### Study procedures

Safety and tolerability assessments were performed through treatment and follow-up and included evaluation of TEAEs, concomitant medications, physical examinations, vital signs, 12-lead ECGs, clinical laboratory assessments, spirometry, and cough symptoms using the validated LCQ^22^ and a cough VAS. Spirometry was performed according to American Thoracic Society (ATS) / European Respiratory Society (ERS) Task Force guidelines^46^ at all clinic visits, with assessments conducted both pre-and post-dose on study drug dosing days. Study staff monitored participants’ self-administration of the study drug (the morning dose) during on-site visits, and symptoms were monitored for at least 1h.

### Bronchoscopy sample collection

Samples collected by bronchoscopy were used to assess LTI-03 lung concentrations and to evaluate pharmacodynamic biomarkers of inhaled LTI-03. Bronchoscopy was performed to collect DBB samples, BALF, and/or bronchoabsorption (BA) samples at baseline and then on Day 14, approximately 2-3 h after the participant self-administered their morning dose of study drug (LTI-03 or placebo) and after all other study assessments had been completed.

Deep bronchial brushings were required for all participants; the site had the option to collect either BAL or BA samples, or both, depending on local expertise, training, and procedural confidence. The sequence of procedures was to be BA, BAL, and then brushings to minimize the risk of blood contamination of samples. Sedation, local anesthesia, and post-procedure monitoring were performed according to the investigative site’s standard of care. Either oral or nasal bronchoscopy routes were acceptable.

Given significant variations in volume of BAL fluid retrieved and that only two sites successfully collected bronchoabsorption samples, the biomarker data presented describe results from DBB samples only.

### Deep bronchial brushings

Bronchoscopists were instructed to obtain a total of 6 brushings from 3 of the basilar bronchial segments of the right lower lobe: the anterior basilar segment, the lateral basilar segment, and the posterior basilar segment. With the bronchoscope proximal to or within the orifice of the appropriate basilar segment, the brush sheath was to be passed out 5 cm beyond the subsegment orifice, and then the brush was fully extended an additional 1 cm for a total of 6 cm beyond the orifice of the subsegmental bronchus. At the desired depth, the sheath was to be gently pulled back and forward for 15 strokes to brush the small airways. The brushing process was repeated using any combination of the 3 basilar segments described above, at the discretion of the bronchoscopist, for a total of 6 six separate brush samples.

### Bronchoalveolar lavage

Bronchoscopists were instructed to lavage a total of 150 mL of normal saline, in 30-50 mL aliquots, to either of the two bronchial segments of the right middle lobe: the lateral segment and the medial segment. If the airways were too small to access the medial or lateral segments, then the bronchoscope could be wedged in the right middle lobe bronchus. With the bronchoscope wedged in the appropriate right middle lobe segment (or right middle lobe bronchus if necessary), 30–50 mL of room temperature or warmed normal saline was injected using an appropriately sized syringe (e.g., 50-60 ml syringe) via tubing attached to the working channel of the bronchoscope lumen. Using the same syringe, the saline was aspirated out through the lumen. Wall suction into a sterile trap could be used as an alternative to syringe aspiration.

Samples for measurement of LTI-03 lung concentrations were collected from the first aspirate.

### Pharmacokinetic analysis

Blood samples for the measurement of systemic LTI-03 concentrations were collected pre-dose (trough) and +5 min after dosing on study Days 1, 7, and 14.

### Biomarker analysis

Blood samples for biomarker assessments in PBMCs and platelet-rich plasma were collected prior to the initial dose and on day 14 following the last dose. Following optimization for detection of analytes in IPF matrices, meso-scale discovery electrochemiluminescence (MSD-ECL) assays were performed at BioAgylitix for SP-D (#K1519XR-2, MSD) in plasma and AKT/pAKT (#K15100D-1, MSD) in PBMCs according to manufacturer’s instructions.

Protein isolation and preparation of DBB samples for ELISAs were performed as previously described^47^ according to manufacturer’s instructions for COL1A1 (#ab210966, abcam), CXCL7 (#LS-F4967-1, LS Bio), GAL7 (#DY1339, R&D Systems), IL-11 (#DY218, R&D Systems), and TSLP (#DY1398, R&D Systems).

### Statistical analyses

All descriptive statistical analyses on measurements other than biomarkers were performed using SAS statistical software (Version 9.4). Missing data were not imputed unless otherwise stated. Continuous data were summarized using the mean, SD, median, minimum value, and maximum value. Categorical data were summarized using frequency counts and percentages. No formal subgroup comparisons by sex, race, or ethnicity were performed.

The primary endpoint was the incidence of TEAEs reported from Day 1 through follow-up. TEAEs were coded using MedDRA (Version 26.1) and graded using the National Cancer Institute Common Terminology for Adverse Events (CTCAE) Version 5. Safety analyses included all participants who received the study drug. A sample size of 18 LTI-03 participants provided a 60% chance of detecting an AE with a true incidence rate of 5% and an 85% chance of detecting a more common AE with a true incidence rate of 10%. Placebo participants in each cohort were pooled for safety and biomarker analyses. No secondary endpoints were specified.

Since exploratory biomarkers were selected based on data generated by prior translational experiments, which demonstrated reduction following LTI-03 treatment in various models, one-tailed statistical analyses were performed. Sample results for placebo-treated participants from each cohort were pooled to increase power (from *n* = 3 to *n* = 6). Due to small sample sizes (*n* = 5 to 9 per group) and non-normality confirmed by Shapiro-Wilk tests (p < 0.05 for TSLP, IL-11, COL1A1, SP-D), a one-tailed Mann-Whitney U test (exact method) was used to compare changes from baseline to Day 14 between the pooled placebo group and each LTI-03 treatment group (5 mg/day and 10 mg/day).

Due to errors in either sample collection (leading to missing samples) or results below the lower limit of detection, there was occasional variability in the total number of samples. For statistical analysis of plasma SP-D, blood samples were inadvertently not collected for the following participants: one in the placebo group (baseline and Day 14 missed), one in the LTI-03 5 mg/day (baseline missed), and a participant in LTI-03 10 mg/day (baseline and Day 14 missed), reducing the total sample size to N = 5 for placebo and N = 8 for each LTI-03 treatment group. Due to a COVID-19 infection, DBB samples were not collected for one participant in the LTI-03 5 mg/day treatment group on Day 14, reducing the total sample size to N = 8 for analyses of all DBB biomarkers in this group. For TSLP, one result was BLQ from one participant in the LTI-03 5 mg/kg treatment group, and the data point was excluded, reducing the sample size for the statistical analysis of TSLP to N = 7.

Descriptive statistics are reported as medians with interquartile ranges (IQR). The Hodges-Lehmann estimator was used for the median difference between groups (treatment minus placebo), with approximate 90% confidence intervals (asymmetric, exact level varied slightly with small n). Cliff’s delta was calculated as effect size using the formula δ = (2U / (n1 × n2)) − 1, with thresholds: |δ | <0.3 small effect, 0.3–0.5 medium effect, >0.5 large effect^48^. The sign was flipped to negative when treatment changes were lower than placebo (indicating reductions). No adjustment for multiplicity was performed, as these were pre-specified, hypothesis-driven exploratory analyses. All biomarker statistical analyses were conducted using GraphPad Prism Version v10.0.

### Reporting summary

Further information on research design is available in the Nature Portfolio Reporting Summary linked to this article.

## Supplementary information

Supplmentary Information

Reporting Summary

Transparent Peer Review file

## Source data

Source data

## Data Availability

De-identified individual participant data for the analyses reported in this article are provided in the Source Data file and include baseline disease characteristics, adverse events, spirometry results, and exploratory biomarker data. Participant-level indirect identifiers, including demographic variables and other potentially identifying study information, are not included because specific written informed consent was not obtained for publication of these data, and disclosure could increase the risk of participant re-identification. Additional participant-level data not provided in the Source Data file, including other clinical source data that are not required to reproduce the reported analyses, may be made available to qualified researchers under controlled access for scientifically valid research purposes, subject to review and approval by Rein Therapeutics Inc. and, where applicable, institutional or ethics approvals. Requests should be submitted to the corresponding author and include a research proposal, analysis plan, intended use, and evidence of appropriate qualifications and approvals. Access will require a data use agreement prohibiting participant re-identification, onward sharing, and use outside the approved purpose. Requests will generally be acknowledged within 30 days; timelines for data access will depend on completion of review, approval, and required agreements. The full study protocol is provided as Supplementary Note 1, and the statistical analysis plan is provided as Supplementary Note 2. Source data are provided with this paper.

## References

[1] Lederer, D. J. & Martinez, F. J. Idiopathic Pulmonary Fibrosis. *N. Engl. J. Med.* **378**, 1811–1823 (2018). 29742380 10.1056/NEJMra1705751

[2] Martinez, F. J. et al. Idiopathic Pulmonary Fibrosis. *Nat. Rev. Dis. Prim.* **3**, 17074 (2017). 29052582 10.1038/nrdp.2017.74

[3] Mei, Q., Liu, Z., Zuo, H., Yang, Z. & Qu, J. Idiopathic Pulmonary Fibrosis: An update on pathogenesis. *Front Pharm.* **12**, 797292 (2022). 10.3389/fphar.2021.797292 PMC8807692 35126134

[4] Richeldi, L. et al. Nerandomilast in Patients with Idiopathic Pulmonary Fibrosis. *N. Engl. J. Med.* **392**, 2193–2202 (2025). 40387033 10.1056/NEJMoa2414108

[5] Belhassen, M., Dalon, F., Nolin, M. & Van Ganse, E. Comparative outcomes in patients receiving pirfenidone or nintedanib for idiopathic pulmonary fibrosis. *Respir. Res* **22**, 135 (2021). 33947414 10.1186/s12931-021-01714-y PMC8094468

[6] Takehara, K. et al. Differential discontinuation profiles between pirfenidone and nintedanib in patients with idiopathic pulmonary fibrosis. *Cells* **11**, 143 (2022). 35011705 10.3390/cells11010143 PMC8750555

[7] Wang, X. M. et al. Caveolin-1: a critical regulator of lung fibrosis in idiopathic pulmonary fibrosis. *J. Exp. Med.* **203**, 2895–2906 (2006). 17178917 10.1084/jem.20061536 PMC1850940

[8] Kulshrestha, R. et al. Caveolin-1 as a critical component in the pathogenesis of lung fibrosis of different etiology: Evidences and mechanisms. *Exp. Mol. Pathol.* **111**, 104315 (2019). 31629729 10.1016/j.yexmp.2019.104315

[9] Fan, J., Zheng, S., Wang, M. & Yuan, X. The critical roles of caveolin-1 in lung diseases. *Front Pharm.* **15**, 1417834 (2024). 10.3389/fphar.2024.1417834 PMC11458383 39380904

[10] Sanders, Y. Y. et al. Epigenetic regulation of caveolin-1 gene expression in lung fibroblasts. *Am. J. Respir. Cell Mol. Biol.* **56**, 50–61 (2017). 27560128 10.1165/rcmb.2016-0034OC PMC5248956

[11] Nagaraja, M. R. et al. p53 Expression in lung fibroblasts is linked to mitigation of fibrotic lung remodeling. *Am. J. Pathol.* **188**, 2207–2222 (2018). 30253845 10.1016/j.ajpath.2018.07.005 PMC6168957

[12] Tian, Y. et al. Quantitative proteomic characterization of lung tissue in idiopathic pulmonary fibrosis. *Clin. Proteom.* **16**, 6 (2019). 10.1186/s12014-019-9226-4 PMC6364390 30774578

[13] Hohmann, M. S., Habiel, D. M., Coelho, A. L., Verri, W. A. & Hogaboam, C. M. Quercetin enhances ligand-induced apoptosis in senescent idiopathic pulmonary fibrosis fibroblasts and reduces lung fibrosis in vivo. *Am. J. Respir. Cell Mol. Biol.* **60**, 28–40 (2019). 30109946 10.1165/rcmb.2017-0289OC PMC6348716

[14] Fan, L. et al. p53-miR-34a Feedback in lung fibroblasts regulates antifibrotic effects of CSP7, nintedanib and pirfenidone. *Am. J. Physiol. Lung Cell Mol. Physiol.* **329**, L480–L498 (2025). 40824943 10.1152/ajplung.00295.2024 PMC12519591

[15] Komatsu, S., Fan, L., Idell, S., Shetty, S. & Ikebe, M. Caveolin-1-derived peptide reduces ER stress and enhances gelatinolytic activity in IPF fibroblasts. *Int J. Mol. Sci.* **23**, 3316 (2022). 35328736 10.3390/ijms23063316 PMC8950460

[16] Venkatesan, S., Fan, L., Tang, H., Konduru, N. V. & Shetty, S. Caveolin-1 scaffolding domain peptide abrogates autophagy dysregulation in pulmonary fibrosis. *Sci. Rep.* **12**, 11086 (2022). 35773303 10.1038/s41598-022-14832-4 PMC9246916

[17] MacKenzie, B. et al. Caveolin scaffolding domain LTI-03 modulates both inflammatory and fibrotic responses in aged mice after bleomycin challenge. *Am. J. Respir. Crit. Care Med.* **205**, A2307 (2022).

[18] MacKenzie, B. et al. LTI-03 peptide demonstrates anti-fibrotic activity in ex vivo lung slices from patients with IPF. *iScience* **28**, 113437 (2025). 41054530 10.1016/j.isci.2025.113437 PMC12496166

[19] Jannini-Sa, Y. A. P., Coelho, A. L., Mackenzie, B. & Hogaboam, C. M. Evaluating alveolar regenerative properties of caveolin scaffolding peptides (CSD) in three dimensional (3D) alveolospheres from IPF and normal donor lung samples. *Am. J. Respir. Crit. Care Med.* **211**, A4691 (2025).

[20] Marudamuthu, A. S. et al. Caveolin-1-derived peptide limits development of pulmonary fibrosis. *Sci. Transl. Med.* **11**, eaat2848 (2019). 31826982 10.1126/scitranslmed.aat2848

[21] Zhang, Y. et al. Development of an excipient-free peptide dry powder inhalation for the treatment of pulmonary fibrosis. *Mol. Pharm.* **17**, 632–644 (2020). 31913640 10.1021/acs.molpharmaceut.9b01085

[22] Birring, S. S. et al. Development of a symptom-specific health status measure for patients with chronic cough: Leicester Cough Questionnaire (LCQ). *Thorax* **58**, 339–343 (2003). 12668799 10.1136/thorax.58.4.339 PMC1746649

[23] Tourkina, E. & Hoffman, S. Caveolin-1 signaling in lung fibrosis. *Open Rheumatol. J.* **6**, 116–122 (2012). 22802909 10.2174/1874312901206010116 PMC3396359

[24] Gvaramia, D., Blaauboer, M. E., Hanemaaijer, R. & Everts, V. Role of caveolin-1 in fibrotic diseases. *Matrix Biol.* **32**, 307–315 (2013). 23583521 10.1016/j.matbio.2013.03.005

[25] Shivani, G. & Lokesh Kumar, B. Caveolin-1: A promising therapeutic target for diverse diseases. *Curr. Mol. Pharm.* **15**, 701–715 (2022). 10.2174/1874467214666211130155902 34847854

[26] Shetty, S. & Idell, S. Caveolin-1-Related intervention for fibrotic lung diseases. *Cells* **12**, 554 (2023). 36831221 10.3390/cells12040554 PMC9953971

[27] Raghu, G. et al. Idiopathic Pulmonary Fibrosis: Prospective, case-controlled study of natural history and circulating biomarkers. *Chest* **154**, 1359–1370 (2018). 30526970 10.1016/j.chest.2018.08.1083

[28] Sgalla, G. et al. Idiopathic pulmonary fibrosis: pathogenesis and management. *Respir. Res.* **19**, 32 (2018). 29471816 10.1186/s12931-018-0730-2 PMC5824456

[29] Permatasari, T. W. & Karuniawati, A. Post-Bronchoscopy infections: A literature review. *J. Clin. Microbiol. Infect. Dis.* **4**, 41–49 (2024).

[30] Guenther, A. et al. The European IPF registry (eurIPFreg): baseline characteristics and survival of patients with idiopathic pulmonary fibrosis. *Respir. Res* **19**, 141 (2018). 30055613 10.1186/s12931-018-0845-5 PMC6064050

[31] Waxman, A. et al. Inhaled treprostinil in pulmonary hypertension due to interstitial lung disease. *N. Engl. J. Med.* **384**, 325–334 (2021). 33440084 10.1056/NEJMoa2008470

[32] Corcoran, T. E., Niven, R., Verret, W., Dilly, S. & Johnson, B. A. Lung deposition and pharmacokinetics of nebulized cyclosporine in lung transplant patients. *J. Aerosol Med Pulm. Drug Deliv.* **27**, 178–184 (2014). 23668548 10.1089/jamp.2013.1042 PMC4088352

[33] Adegunsoye, A. et al. Circulating plasma biomarkers of survival in antifibrotic-treated patients With idiopathic pulmonary fibrosis. *Chest* **158**, 1526–1534 (2020). 32450241 10.1016/j.chest.2020.04.066 PMC7545483

[34] He, X. et al. Serum surfactant protein D as a significant biomarker for predicting occurrence, progression, acute exacerbation, and mortality in interstitial lung disease: a systematic review and meta-analysis. *Front Immunol.* **16**, 1450798 (2025). 40028331 10.3389/fimmu.2025.1450798 PMC11868069

[35] Ikeda, K. et al. Serum surfactant protein D as a predictive biomarker for the efficacy of pirfenidone in patients with idiopathic pulmonary fibrosis: a post-hoc analysis of the phase 3 trial in Japan. *Respir. Res.* **21**, 316 (2020). 33256760 10.1186/s12931-020-01582-y PMC7706186

[36] Jenkins, R. G. et al. Effects of nintedanib on circulating biomarkers of idiopathic pulmonary fibrosis. *ERJ Open Research*, 00558-2023 (2024). 10.1183/23120541.00558-2023 PMC11647937 39687396

[37] Maher, T. M. et al. Circulating biomarkers and progression of idiopathic pulmonary fibrosis: data from the INMARK trial. *ERJ Open Res.* **10**, 00335-2023 (2024). 39040590 10.1183/23120541.00335-2023 PMC11261372

[38] Schafer, P. et al. Development of a multi-biomarker risk score based on serum proteins by the Prognostic Lung Fibrosis Consortium (PROLIFIC). *Eur. Respir. J.* **64**, PA4257 (2024).

[39] Lee, J. et al. Bronchoalveolar lavage (BAL) cells in idiopathic pulmonary fibrosis express a complex pro-inflammatory, pro-repair, angiogenic activation pattern, likely associated with macrophage iron accumulation. *PLoS One* **13**, e0194803 (2018). 29649237 10.1371/journal.pone.0194803 PMC5896901

[40] Zhao, T. et al. Identification and validation of chemokine system-related genes in idiopathic pulmonary fibrosis. *Front Immunol.* **14**, 1159856 (2023). 37122736 10.3389/fimmu.2023.1159856 PMC10140527

[41] Ng, B. et al. Interleukin-11 is a therapeutic target in idiopathic pulmonary fibrosis. *Sci. Transl. Med.* **11**, eaaw1237 (2019). 31554736 10.1126/scitranslmed.aaw1237

[42] Ebina-Shibuya, R. & Leonard, W. J. Role of thymic stromal lymphopoietin in allergy and beyond. *Nat. Rev. Immunol.* **23**, 24–37 (2023). 35650271 10.1038/s41577-022-00735-y PMC9157039

[43] Lee, J. U. et al. Upregulation of interleukin-33 and thymic stromal lymphopoietin levels in the lungs of idiopathic pulmonary fibrosis. *BMC Pulm. Med.* **17**, 39 (2017). 28202030 10.1186/s12890-017-0380-z PMC5312598

[44] Datta, A. et al. Evidence for a functional thymic stromal lymphopoietin signaling axis in fibrotic lung disease. *J. Immunol.* **191**, 4867–4879 (2013). 24081992 10.4049/jimmunol.1300588 PMC3836180

[45] Raghu, G. et al. Diagnosis of Idiopathic Pulmonary Fibrosis. An Official ATS/ERS/JRS/ALAT Clinical Practice Guideline. *Am. J. Respir. Crit. Care Med.* **198**, e44–e68 (2018). 30168753 10.1164/rccm.201807-1255ST

[46] Miller, M. R. et al. Standardisation of spirometry. *Eur. Respir. J.* **26**, 319–338 (2005). 16055882 10.1183/09031936.05.00034805

[47] Espindola, M. S. et al. Translational studies reveal the divergent effects of simtuzumab targeting LOXL2 in Idiopathic Pulmonary Fibrosis. *Fibros. (Hong. Kong)* **1**, 10007 (2023). 10.35534/fibrosis.2023.10007 PMC11175361 38873180

[48] Romano, J., Kromrey, J. D., Coraggio, J. & Skowronek, J. Appropriate statistics for ordinal level data: should we really be using t-test and Cohen’s d for evaluating group differences on the NSSE and other surveys? In *Proceedings of the Annual meeting of the Florida Association of Institutional Research*, Cocoa Beach, FL, 1-3 February (2006).

