# Supplementary material for: Inhaled LTI-03 for idiopathic pulmonary fibrosis: a randomized dose escalation study
Source: Nat Commun. 2026 Jul 31;17:7620. doi: 10.1038/s41467-026-75291-3 (PMC13427755; doi:10.1038/s41467-026-75291-3)
Supplement: Supplementary file 1 — Supplmentary Information [file 41467_2026_75291_MOESM1_ESM.pdf]

Inhaled LTI-03 for Idiopathic Pulmonary Fibrosis: A Randomized Dose Escalation Study

Supplementary Information

Table of Contents

Supplementary Table 1. Summary of All Treatment Emergent Adverse Events

Supplementary Fig. 1. By-Patient Change in Lung Function Over Time.

Supplementary Fig. 2. Exploratory biomarkers, change from baseline compared to placebo.

Supplementary Note 1: Clinical Study Protocol for Study LTI-03-1002

Supplementary Note 2: Statistical Analysis Plan for Study LTI-03-1002

Supplementary Note 3: CONSORT 2010 Checklist

Supplementary Table 1. Summary of All Treatment Emergent Adverse Events

Event<br>Preferred Term, n (%)	LTI-03<br>5 mg/day<br>(N=9)	LTI-03<br>10 mg/day<br>(N=9)	LTI-03<br>Pooled<br>(N=18)	Placebo<br>Pooled<br>(N=6)
**Any TEAE**	6 (66.7)	7 (77.8)	13 (72.2)	3 (50.0)
Cough	3 (33.3)[2]	5 (55.6)[2]	8 (44.4)	2 (33.3)
Rhinorrhea	1 (11.1)	1 (11.1)	2 (11.1)	0
Dyspnea, exertional	1 (11.1)	0	1 (5.6)	0
Productive cough	0	1 (11.1)	1 (5.6)	0
Throat irritation	0	1 (11.1)	1 (5.6)	0
Dry mouth	0	1 (11.1)	1 (5.6)	0
Hypoesthesia oral	0	1 (11.1)	1 (5.6)	0
Esophageal spasm	0	0	0	1 (16.7)
Dizziness	0	0	0	1 (16.7)
Dysgeusia	1 (11.1)	0	1 (5.6)	0
Ophthalmic migraine	1 (11.1)	0	1 (5.6)	0
Chest discomfort	1 (11.1)	0	1 (5.6)	0
Fatigue	1 (11.1)	0	1 (5.6)	0
COVID-19	1 (11.1)	0	1 (5.6)	0
Nasopharyngitis	0	1 (11.1)	1 (5.6)	0
Palpitations	0	1 (11.1)	1 (5.6)	0
Vertigo	0	1 (11.1)	1 (5.6)	0
Post procedural fever[1,2]	0	1 (11.1)[1]	1 (5.6)	0
Body temperature increased	1 (11.1)	0	1 (5.6)	0
Hyperkaliemia	1 (11.1)	0	1 (5.6)	0
Back pain	1 (11.1)	0	1 (5.6)	
Cold sweat	0	1 (11.1)	1 (5.6)	0
**TEAEs Related to Study Drug**	2 (22.2)	5 (55.6)	7 (38.9)	1 (16.7)
Cough	2 (22.2)[2]	4 (44.4)[2]	6 (33.3)	1 (16.7)
Throat Irritation	0	1 (11.1)	1 (5.6)	0
Dry Mouth	0	1 (11.1)	1 (5.6)	0
Hypoesthesia oral	0	1 (11.1)	1 (5.6)	0
Palpitations	0	1 (11.1)	1 (5.6)	0

TEAE, Treatment-related Adverse Event: an AE which began or increased in severity or frequency at or after the administration of first study drug. Unless otherwise noted, TEAEs were reported as Grade 1 (mild) in severity, non-serious, and unrelated to study drug.

[1]Serious TEAE

[2] Grade 2 (moderate severity) TEAEs: cough for 1 participant in each LTI-03 treatment group, post-procedural fever for 1 participant (LTI-03 10 mg/day).

Supplementary Fig. 1. By-Patient Change in Lung Function Over Time.

Panels A, C, E: Individual data points represent percent (%) change values from baseline in FEV_1 (L) over time for placebo, LTI-03 5mg/day and LTI-03 10 mg/day, respectively.

Panels B, D, F: Individual data points represent absolute FVC (L) values over time for placebo, LTI-03 5mg/day and LTI-03 10 mg/day, respectively. Black arrows in panel B and panel F denote the participant for whom LTI-03 10mg/day dosing was incorrectly discontinued on Day14. This participant had decreases in FEV_1 and FVC from baseline of similar magnitude prior to dosing on Day 14, with an FEV_1 /FVC ratio decreased by 2.5% from baseline. This participant was clinically asymptomatic and assessed as having no clinical evidence of airway obstruction. Of note, the participant (10 mg/day dose group) in panel C with the largest decreases in FEV_1 was also clinically asymptomatic and assessed as having no evidence of airway obstruction.

Supplementary Fig.1

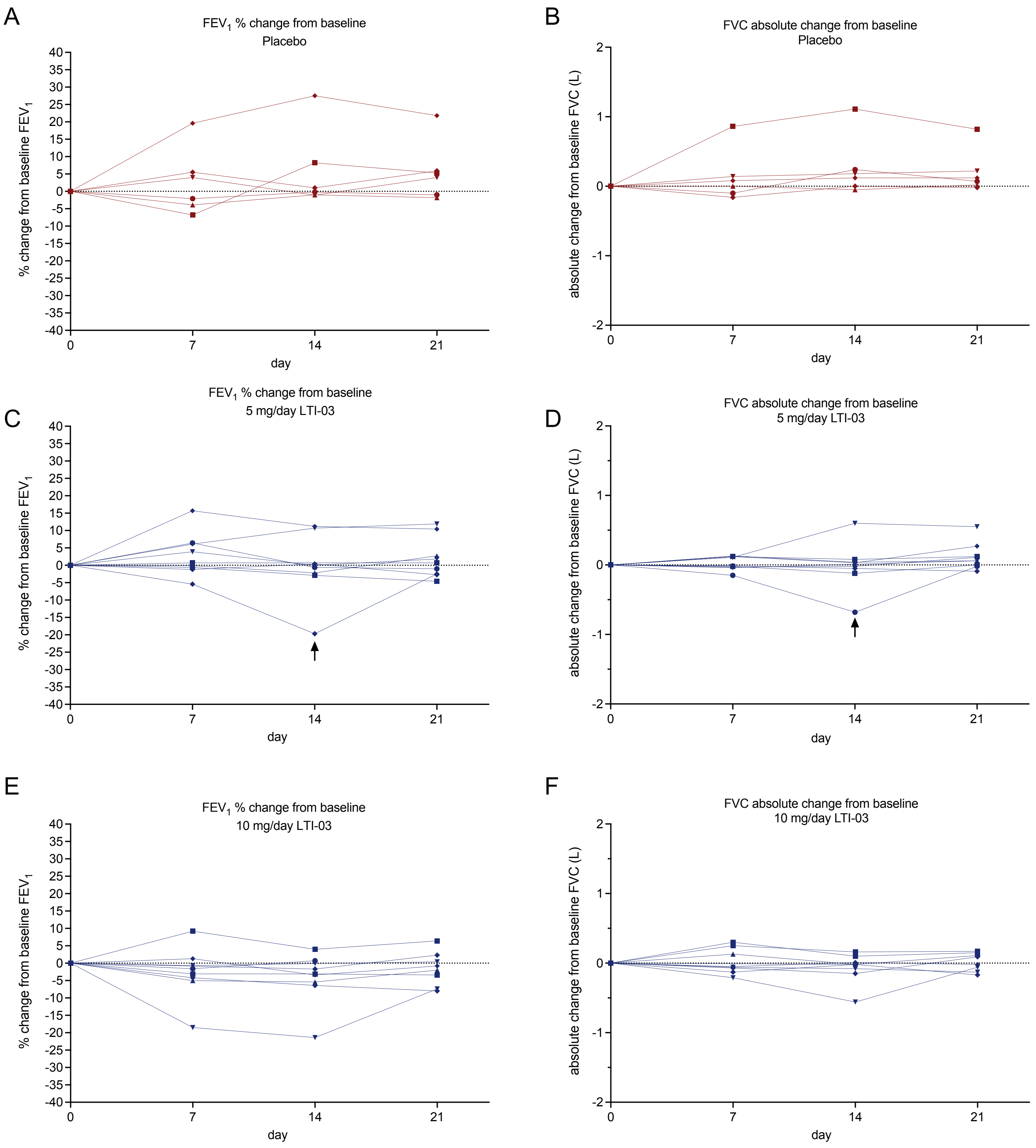

Supplementary Fig. 2. Exploratory biomarkers, change from baseline compared to placebo. Violin plots showing interquartile ranges with individual analyte change in baseline values overlaid are shown. One-tailed, unpaired, non-parametric Mann-Whitney U tests were performed on analyte changes from baseline (pre-dose) to Day 14 for placebo (pooled) vs. LTI-03 5 mg/day or LTI-03 10 mg/day. A p-value of ≤ 0.05 was considered statistically significant. N: total number of samples. Data points were excluded either due to missed sample collection or an analyte measurement being below lower limit of quantitation (BLQ).

DBB, deep bronchial brushings; SCR, screening (baseline); SP-D, Surfactant Protein D; *%p-AKT* % phospho-AKT/total AKT in PBMCs; COL1A1*,* collagen type 1 alpha chain 1; CXCL-7, chemokine (C-X-C motif) ligand 7; GAL-7, galectin-7; IL-11 interleukin 11; TSLP, thymic stromal lymphopoietin. Units: SP-D (pg/mL in plasma); %p-AKT (% phospho-AKT/total AKT in PBMCs); COL1A1, CXCL7, GAL7, IL-11, TSLP (pg/mg protein) or ng/mg protein for CXCL7. No multiple comparison correction applied (exploratory analysis).

Supplementary Fig. 2.

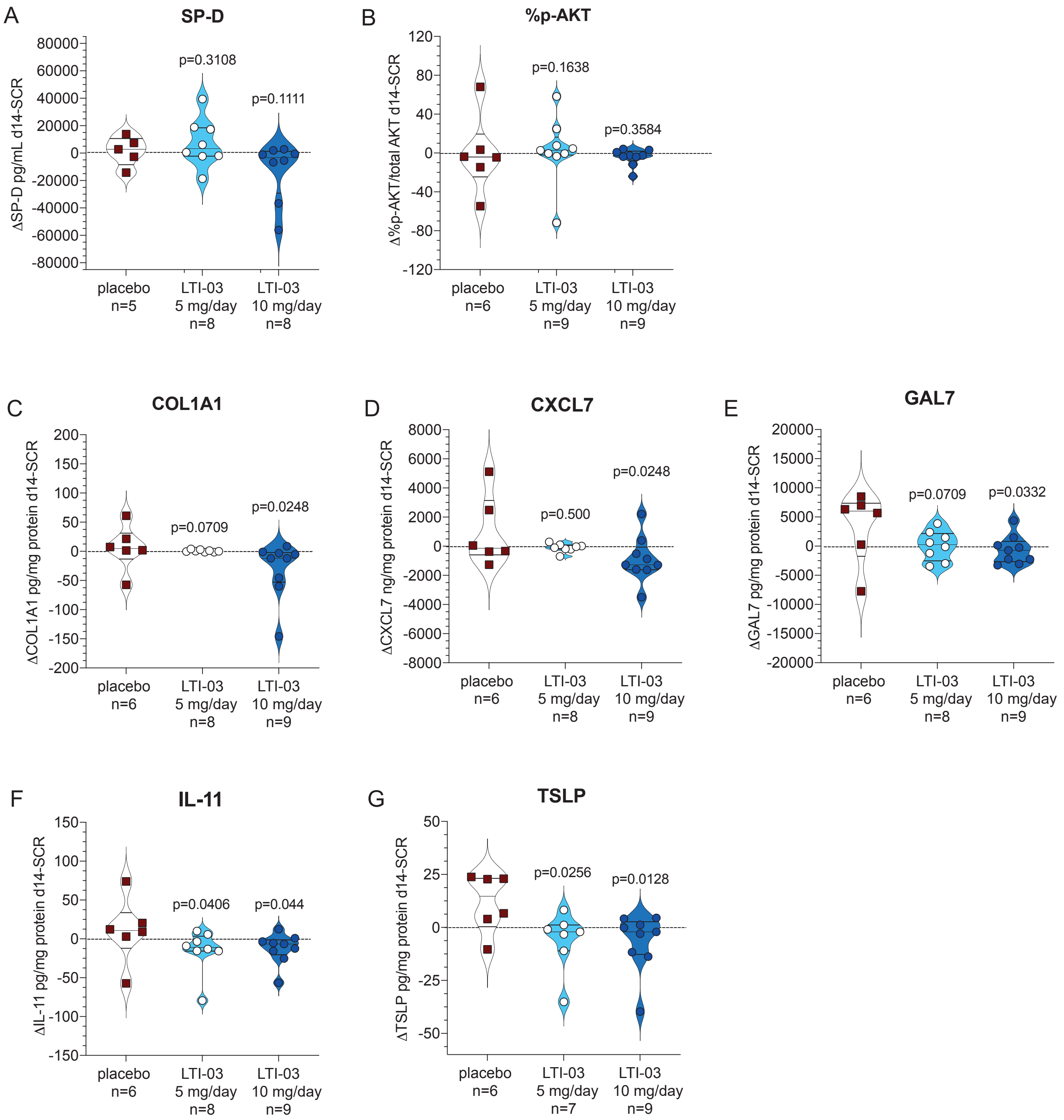

Supplementary Note 1: Clinical Protocol for Study LTI-03-1002

CLINICAL STUDY PROTOCOL

TITLE: A Randomized, Double-Blind, Placebo-Controlled, Phase 1b, Dose Escalation, Safety, Tolerability and Pharmacodynamic Biomarker Study of Caveolin-1-Scaffolding-Protein-Derived Peptide (LTI-03) in Recently Diagnosed, Treatment Naïve Subjects with Idiopathic Pulmonary Fibrosis

PROTOCOL NUMBER: LTI-03-1002

EUDRACT NUMBER: 2022-000083-21

TEST PRODUCT: LTI-03

SPONSOR: Lung Therapeutics, Inc.
3801 S. Capital of Texas Hwy Suite 330
Austin, Texas 78704

VERSION: 5.0

DATE OF PROTOCOL: 04 May 2023

SPONSOR PROTOCOL APPROVAL SIGNATURE:

DocuSigned by:
Steven Shoemaker
Signer Name: Steven Shoemaker
Signing Reason: I approve this document
Signing Time: 08-May-2023 | 08:34 MDT
989FDB26F12C48B6B6C18581BC6B1F34

Steven Shoemaker, MD
Acting Chief Medical Officer

CONFIDENTIALITY STATEMENT: The information in this document is confidential and is not to be disclosed without the written consent of Lung Therapeutics Inc. except to the extent that disclosure would be required by law and for the purpose of evaluating and/or conducting a clinical study for Lung Therapeutics Inc.

STATEMENT OF COMPLIANCE: This study will be conducted in accordance with the ethical principles that have their origin in the Declaration of Helsinki, and clinical research guidelines established by the United States Code of Federal Regulations (21 CFR Parts 50, 56, and 312), and the International Council for Harmonisation (ICH) E6(R2) Guidelines on Good Clinical Practice (GCP). This study will also adhere to the European Medicines

Authority (EMA), Medicines and Healthcare Products Regulatory Agency (MHRA), country, state and/or local regulatory requirements.

INVESTIGATOR AGREEMENT

**TITLE:**	A Randomized, Double-Blind, Placebo-Controlled, Phase 1b, Dose Escalation, Safety, Tolerability and Pharmacodynamic Biomarker Study of Caveolin-1-Scaffolding-Protein-Derived Peptide (LTI-03) in Recently Diagnosed, Treatment Naïve Subjects with Idiopathic Pulmonary Fibrosis
**PROTOCOL NUMBER:**	LTI-03-1002
**TEST PRODUCT:**	LTI-03
**VERSION:**	5.0
**DATE OF PROTOCOL:**	04 May 2023

This clinical study protocol has been subjected to critical review and has been released by the Sponsor. The information it contains is consistent with current risk and benefit evaluation of the investigational medicinal product, as well as with the moral, ethical, and scientific principles governing clinical research as set out in the Declaration of Helsinki (Version 2013), and the guidelines on Good Clinical Practices (GCP) applicable to this clinical study.

I have read the Protocol LTI-03-1002 and agree to conduct the study according to its terms. I understand that all information relating to LTI-03 and this protocol supplied to me by Lung Therapeutics, Inc. is confidential.

Principal Investigator's Name (print) Site Number

Principal Investigator's Signature Date

Please retain the signed original of this form for your study files. Please return a copy of the signed form as instructed.

TABLE OF CONTENTS

List of Abbreviations **8**

Protocol Synopsis **12**

Key Study Contacts **18**

1 Background Information And Scientific Rationale **19**

1.1 Background Information 19
1.1.1 LTI-03 19
1.2 Scientific Rationale 22
1.2.1 Dose Rationale 22
1.3 Potential Risks and Benefits 22

2 Study Objectives And Endpoints **24**

2.1 Objectives 24
2.1.1 Primary Objective 24
2.1.2 Exploratory Objectives 24
2.2 Endpoints 24
2.2.1 Primary Endpoint 24
2.2.2 Other Safety Endpoints 24
2.2.3 Exploratory Endpoints 24

3 Study Design **25**

4 Study Subjects **26**

4.1 Eligibility Criteria 26
4.1.1 Inclusion Criteria 26
4.1.2 Exclusion Criteria 26

5 Study Drug **29**

5.1 Formulation, Packaging and Labeling 29
5.2 Storage and Stability 29
5.3 Accountability 29
5.4 Administration and Management of Study Drug 29
5.5 Method of Assignment to Treatment 30
5.6 Concomitant and Prohibited Medications 31

6 Study Procedures **32**

6.1 Informed Consent 32
6.2 Demographics and Medical History 32
6.3 Vital Signs 32
6.4 Physical Examination 32
6.5 Clinical Laboratory Assessments 33
6.5.1 Pregnancy Test 33
6.6 Electrocardiogram 33

6.7 Spirometry 34
6.8 Inhaler Use Assessment 34
6.9 Bronchoscopy with Bronchoalveolar Lavage and/or Bronchoabsorption and Deep Bronchial Brushing 34
6.10 Leicester Cough Questionnaire 35
6.11 Cough Visual Analogue Scale 35
6.12 Pharmacokinetic Sampling 35
6.13 Exploratory Biomarker Sampling 35
6.14 Treatment, Subject, Study and Site Discontinuation 36
6.14.1 Premature Discontinuation of Study Drug 36
6.14.2 Subject Withdrawal of Consent 36
6.14.3 Replacement of Subjects 37
6.14.4 Study Discontinuation 37
6.14.5 Site Discontinuation 37

7 Study Schedule 38

7.1 Screening Period (Day -21 to Day -1) 38
7.2 Treatment Period 39
7.2.1 Day 1 39
7.2.2 Days 2-6 40
7.2.3 Day 7 40
7.2.4 Days 8-13 41
7.2.5 Day 14 41
7.3 Follow-up Period and Day 21 Visit 42
7.4 Early Termination 42

8 Assessment of Safety 43

8.1 Specification of Safety Parameters 43
8.2 Definitions of Adverse Events 43
8.2.1 Adverse Event 43
8.2.2 Serious Adverse Event 43
8.3 Adverse Event Classification 44
8.3.1 Relationship to Investigational Product 44
8.3.2 Severity 44
8.4 Collection and Reporting of Adverse Events 45
8.4.1 Initial Reporting of Adverse Events 45
8.4.2 Adverse Events Reporting Period 46
8.4.3 Follow-up of Adverse Events 46
8.5 Collection and Reporting of Serious Adverse Events 46
8.5.1 Initial Serious Adverse Event Reports 46
8.5.2 Follow-up of Serious Adverse Events 47
8.5.3 Reporting of Death 47

8.6 Post-Trial Adverse Events 47
8.7 Pregnancy Reporting and Follow-up 47
8.8 Stopping Rules 48
8.9 Emergency Unblinding 49
8.10 Emergency Sponsor Contact 49

9 Statistical Considerations 50

9.1 Statistical and Analytical Plans 50
9.1.1 Randomization Procedures 50
9.2 Study Hypotheses 50
9.3 Analysis Set 50
9.3.1 Safety Analysis Set 50
9.4 Statistical Methods and Analysis 50
9.4.1 General Approach 50
9.4.2 Demographics and Other Baseline Characteristics 50
9.4.3 Concomitant Medications 51
9.4.4 Primary Endpoint 51
9.4.5 Other Safety Endpoints 51
9.4.6 Analysis of Exploratory Endpoints 52
9.4.7 Planned Interim Analyses 52
9.5 Sample Size Determination 52

10 Data Handling and Record Keeping 53

10.1 Study Files and Subject Source Documents 53
10.2 Data Collection Methods 53
10.3 Retention of Records 54
10.4 Protocol Deviations 54

11 Quality Control and Quality Assurance 55

12 Ethics / Protection of Human Subjects 56

12.1 Ethical Conduct of the Study 56
12.2 Ethics Committee Review 56
12.3 Informed Consent Process 56
12.4 Confidentiality of Information 57
12.5 Future Use of Stored Specimens 57
12.6 Publication Policy 57

13 References 59

14 Appendices 60

14.1 Appendix 1 Schedule of Assessments 60
14.2 Appendix 2 Visit and Procedure Windows 62
14.3 Appendix 3 Leicester Cough Questionnaire 63
14.4 Appendix 4 Cough Visual Analogue Scale 64

14.5 Appendix 5 Protocol Amendments 65

LIST OF TABLES

Table 1 Instructions on Multiple Administrations taken at a Time and for Missing the Dose 30
Table 2 Clinical Laboratory Assessments 33
Table 3 Exploratory Biomarkers by Sample Source and Assumed Indicator Function 35
Table 4 Adverse Event Severity Grading Scale for Events not Specifically Listed in NCI CTCAE 45
Table 5 Schedule of Assessments 60

LIST OF ABBREVIATIONS

^{3}H	hydrogen isotope tritium
AE	adverse event
AECs	alveolar epithelial cells
AEC2	alveolar epithelial type 2 cells
ALP	alkaline phosphatase
ALT	alanine aminotransferase
aPTT	activated partial thromboplastin time
AST	aspartate aminotransferase
ATS	American Thoracic Society
AV	atrioventricular
BA	bronchoabsorption
BAL	bronchoalveolar lavage
BALF	bronchoalveolar lavage fluid
BD	twice daily
BLM	bleomycin
BSA	body surface area
BUN	blood urea nitrogen
Cav-1	caveolin-1
CFR	Code of Federal Regulations
CK	creatine kinase
C_{max}	maximum plasma concentration
Col-1	type 1 collagen
COVID-19	coronavirus disease 2019
CRA	Clinical Research Associate
CRF	case report form
CRO	Contract Research Organization
CRP	C-reactive protein
CSD	caveolin-1 scaffolding domain
C_{ss}	steady state concentration

CTCAE	Common Terminology Criteria for Adverse Events
DLCO	diffusion capacity of the lungs for carbon monoxide
EC	Ethics Committee
ECG	electrocardiogram
ECM	extracellular matrix
eGFR	estimated glomerular filtration rate
ELF	extracellular lining fluid
EMA	European Medicines Authority
EOS	end of study
ERS	European Respiratory Society
EU	European Union
FEV_1	forced expiratory volume 1
FLF	fibrotic lung fibroblast
FN	fibronectin
FOCBP	female of childbearing potential
FVC	forced vital capacity
GCP	Good Clinical Practice
GGT	gamma glutamyltransferase
GLP	Good Laboratory Practice
HDL-C	high density lipoprotein cholesterol
Hgb	hemoglobin
HR	Heart rate
HRCT	high-resolution computed tomography
IB	investigator brochure
ICF	informed consent form
ICH	International Council for Harmonisation
ILD	interstitial lung disease
IPF	idiopathic pulmonary fibrosis
IRT	interactive response technology
ITT	intent-to-treat

LDH	lactate dehydrogenase
LDL-C	low density lipoprotein cholesterol
MAD	multiple ascending dose
MCH	mean corpuscular hemoglobin
MCHC	mean corpuscular hemoglobin concentration
MCV	mean corpuscular volume
MedDRA	Medical Dictionary for Regulatory Activities
MHRA	Medicines and Healthcare Products Regulatory Agency
NCI	National Cancer Institute
NOAEL	no observed adverse effect level
PD	Pharmacodynamic
PK	pharmacokinetic
PP	per protocol
PT	prothrombin time
QD	once daily
QTcF	QT interval corrected for heart rate calculated using Fridericia's formula
RBC	red blood cell
RDW	red cell distribution width
RNA	ribonucleic acid
RR	Respiratory rate
SAD	single ascending dose
SAE	serious adverse event
SAP	statistical analysis plan
SAS	Statistical Analysis System
α-SMA	alpha-smooth muscle actin
SOA	schedule of assessment
SOP	Standard Operating Procedure
SP-C	surfactant protein-C
TEAE	treatment-emergent adverse event
TN-C	tenascin-C

US	United States
VAS	visual analogue scale
WBC	white blood cell
WHO-DD	World Health Organization Drug Dictionary

PROTOCOL SYNOPSIS

**Sponsor**	Lung Therapeutics, Inc
**Protocol Number:**	LTI-03-1002
**Protocol Title:**	A Randomized, Double-Blind, Placebo-Controlled, Phase 1b, Dose Escalation, Safety, Tolerability and Pharmacodynamic Biomarker Study of Caveolin-1-Scaffolding-Protein-Derived Peptide (LTI-03) in Recently Diagnosed, Treatment Naïve Subjects with Idiopathic Pulmonary Fibrosis
**Phase:**	1b
**Investigational Product**	**Active Study Drug**<br>LTI-03 as micronized dry powder in hard 2-piece hypromellose capsules.<br>**Matching Placebo**<br>Placebo as micronized lactose powder in capsule.<br>**Inhalation device:**<br>Dry powder Inhaler.
**Study Subjects**	The study subject population will consist of patients with idiopathic pulmonary fibrosis (IPF) without treatment with anti-fibrotic agents within 2 months of the Baseline bronchoscopy.
**Number of Subjects Planned**	Approximately 24 subjects will be randomized in the study.
**Duration of Participation**	Each subject's duration of participation is up to 42 days, including 21 days of Screening Period, 14 days for Treatment Period, and 7 days post-treatment Follow-up Visit.
**Investigational Sites**	Approximately 9 investigational sites.
**OBJECTIVES**	**Primary Objective:**<br>• To determine the safety and tolerability of inhaled LTI-03 in subjects with IPF<br>**Exploratory Objectives:**<br>• To assess the plasma pharmacokinetic (PK) of inhaled LTI-03 after the first dose and at steady state in subjects with IPF<br>• To evaluate the pharmacodynamic (PD) biomarkers of inhaled LTI-03 in subjects diagnosed with IPF

**ENDPOINTS**	**Primary Endpoint:**<br>• Incidence of treatment-emergent adverse events (TEAEs).<br>**Other Safety Endpoints:**<br>• Physical examination findings.<br>• Vital signs.<br>• Electrocardiogram (ECG).<br>• Clinical laboratory measurements.<br>• Spirometry.<br>• Leicester Cough Questionnaire<br>• Cough Visual Analogue Scale<br>**Exploratory Endpoints:**<br>• Plasma peak and trough concentration of LTI-03 after the first dose and at steady state.<br>• Change from baseline over time for biomarkers related to the pathophysiology of IPF.
**STUDY DESIGN**	This is a randomized, double-blind, placebo-controlled, multi-center, dose escalation, safety, and tolerability study of LTI-03 or placebo administered by inhalation in subjects with IPF who have not been treated with anti-fibrotic agents within 2 months of the Baseline bronchoscopy. An IPF diagnosis will be confirmed by high-resolution computed tomography (HRCT) of chest or lung biopsy within 3 years prior to Screening.<br>The study will contain 2 dose cohorts which will run sequentially. Subjects in the first, low-dose cohort will receive 2.5 mg of study drug (LTI-03 or placebo), administered twice daily approximately 10-12 hours apart (5 mg per day). If the 5 mg per day dose is well tolerated, a second, high-dose cohort will receive 5 mg of study drug (LTI-03 or placebo), administered twice daily approximately 10-12 hours apart (10 mg per day).<br>Eligible subjects will be randomized in a 3:1 ratio to either LTI-03 or placebo. Each cohort will have 9 subjects randomized to LTI-03 and 3 subjects randomized to placebo. Safety data will be reviewed on an ongoing basis by the Sponsor's medical officer and the Contract Research Organization (CRO)'s medical monitor. A review of safety data including but not limited to adverse events, laboratory assessments, vital signs, and spirometry will be conducted after all 12 subjects from Cohort 1 have completed Day 14 assessments. Enrollment in the second cohort will not begin until the Cohort 1 safety data has been reviewed and a dose decision

	made. A lower dose cohort may be added (2.5 mg administered once daily) or additional subjects (up to 12 subjects) may be added to the low-dose cohort (2.5 mg administered twice daily) if doses of 5 mg twice daily (10 mg per day) are not tolerated. In total, a maximum of approximately 24 subjects will be randomized in the study.<br><br>Screening assessments will take place over no more than 21 days prior to the Treatment Period. Screening assessments will include but not be limited to a complete physical examination including vital signs, ECG, spirometry, safety laboratory parameters, medical history, and concomitant medications. A Baseline bronchoscopy will be performed during the Screening Period in subjects who meet the Screening criteria and will include bronchoalveolar lavage and/or bronchoabsorption, and deep bronchial brushings.<br><br>The Treatment Period will be 14 days, with subjects self-administering the study drug using the provided commercially available dry powder inhaler. All subjects will be trained on the proper methods of inhaler use and will have access to site personnel trained to assist subjects remotely if questions arise. Retraining may occur on Day 1 and 7, if required.<br><br>Follow-up study assessments will be completed at Day 21 with all Day 14 assessments repeated except dosing, bronchoscopy, and blood collection for biomarker and PK. Also, complete physical examination will be replaced by brief physical examination.
**ELIGIBILITY CRITERIA**	**Inclusion Criteria**<br><br>Individuals must meet all the following inclusion criteria to be eligible for participation in this study.<br><br>1. Male or female subject of age 40 years or older.<br>2. Willing and able to provide written informed consent.<br>3. Diagnosis of IPF within 3 years of Screening as confirmed by HRCT of chest or lung biopsy as defined by ATS/ERS/JRS/ALAT guideline.<br>4. Forced vital capacity (FVC) percent predicted ≥ 40%.<br>5. Diffusion capacity of the lungs for carbon monoxide (DLCO) percent predicted ≥ 30 and ≤ 80.<br>6. Forced expiratory volume 1 (FEV_1)/FVC ≥ 0.7.<br><br>**Exclusion Criteria**<br><br>Individuals who meet *any* of the following exclusion criteria will not be enrolled in this study.

1. Interstitial lung disease other than IPF.
2. Evidence of significant obstructive lung disease.
3. Current diagnosis of asthma.
4. Treatment with an approved or investigational antifibrotic therapy for IPF within 2 months of the Baseline bronchoscopy.
5. Use of N-acetyl cysteine or other supplements within 7 days prior to dosing and throughout the Treatment Period.
6. Inability to use study inhaler device appropriately.
7. Pulmonary exacerbation within 6 months prior to Screening.
8. Febrile illness within 7 days prior to dosing.
9. Participation in a clinical study or treatment with an investigational drug or device within 30 days of the Screening Visit (or 5 half-lives of the investigational agent, whichever is longer).
10. History or evidence at screening of significant renal impairment with eGFR < 30 mL/min.
11. History or evidence at screening of significant hepatic impairment with bilirubin > 3 mg/dL (> 51.3 µmol/L) and albumin < 2.8 g/dL (<28 g/L) and PT prolongation > 6 sec or INR > 2.3.
12. Serious or active medical or psychiatric condition which, in the opinion of the Investigator, may interfere with treatment, assessment, or compliance with the protocol.
13. Vaccination within 2 weeks of start of dosing (Day 1) and throughout the Treatment Period.
14. Subject has severe progressive or uncontrolled, clinically significant disease that in the judgment of the investigator or designee renders the subject unsuitable for the study.

Contraception and Pregnancy

15. Positive pregnancy test in female subjects of childbearing potential as defined below.
16. Female subjects who are lactating.
17. Females of childbearing potential (FOCBP) and men with partners of childbearing potential who do not agree to use an acceptable form of contraception for the duration of study treatment and for at least 90 days after the last dose of study drug. Male subjects who do not agree to refrain from donating sperm during this same period.

	– These methods of contraception are acceptable: Bilateral tubal ligation; male sterilization; hormonal contraceptives that inhibit ovulation; hormone-releasing intrauterine devices; and copper intrauterine devices.<br>– True abstinence when this is in line with the preferred and usual lifestyle of the subject. Periodic abstinence (e.g., calendar, ovulation, symptothermal post-ovulation methods) and withdrawal are not acceptable methods of contraception.<br>– Male sterilization (with appropriate post-vasectomy documentation of the absence of sperm in the ejaculate). For female subjects, the vasectomized male partner must be documented as the sole partner.<br>Contraceptive requirements do not apply for subjects who are exclusively in same sex relationships. If a subject who is in a same sex relationship at the time of signing the informed consent form (ICF) becomes engaged in a heterosexual relationship, they must agree to use contraception as described and as outlined in the protocol and ICF.<br>NOTE: Female subjects who are surgically sterile or post-menopausal for at least 12 months without any other underlying medical cause are not considered to be of childbearing potential.
**STATISTICAL METHODS**	**Sample Size Justification:**<br>Approximately 24 subjects are planned in 2 cohorts. Each cohort will include 12 subjects (9 on LTI-03 and 3 on placebo). Selection of sample size is based on prior experience to ensure that the safety and tolerability of LTI-03 will be adequately assessed while minimizing unnecessary subject exposure. With 18 subjects receiving LTI-03 there is a 60% chance of detecting an adverse event (AE) with a true incidence rate of 5% and an 85% chance of detecting a more common AE with a true incidence rate of 10%.<br>**Statistical Analysis:**<br>The primary endpoint of the study is safety. No formal statistical hypotheses will be tested.<br>All descriptive statistical analyses will be performed using the most recently released and available Statistical Analysis System (SAS) statistical software (version 9.4 or higher), unless otherwise noted. For categorical variables, the number and percent of each category within a parameter will be

	presented. For continuous variables, the sample size (n), mean, median, and standard deviation, as well as the minimum and maximum values, will be presented. Missing data will not be imputed unless otherwise stated. Subjects on Placebo from all cohorts will be pooled to form a combined placebo group for analysis.<br><br>The primary endpoint of the study is incidence of TEAEs.<br><br>Adverse events will be graded according to National Cancer Institute (NCI) Common Terminology Criteria for Adverse Events (CTCAE), version 5.0.<br><br>Adverse events will be coded by preferred term and system organ class using the current version of Medical Dictionary for Regulatory Activities (MedDRA®) and summary tables for all AEs will be generated for the Safety Population. Incidence rates and percentages will be summarized for each preferred term and system organ class. Additional summary tables will be generated for the following population subsets: subjects with serious adverse events (SAE)s, subjects with related AEs, subjects with severe (Grade 3 or 4) AEs, and subjects who discontinue study drug due to AEs. Adverse events will be summarized by treatment and\or cohort. Severity, Investigator-attributed relationship to study drug, and action taken will also be recorded.<br><br>**Planned Interim Analysis:**<br><br>No formal interim analyses will be performed in the study. Cohort 1 biomarker data may be analyzed before final database lock without changing the overall study conduct.

KEY STUDY CONTACTS

A contact information list of the Sponsor will be provided separately.

Sponsor Medical Officer:

Name:	Steven Shoemaker, MD<br>Acting Chief Medical Officer<br>Lung Therapeutics, Inc.
Phone number:	+ 1 720 560 2167
Email:	sshoemaker@nicosof.com

Other Study Contact:

Name:	Anne Kinney, MSPH<br>Associate Director, Clinical Operations<br>Lung Therapeutics, Inc.
Phone number:	+1 737 802 1990
Email:	akinney@lungtx.com

1 Background Information And Scientific Rationale

1.1 Background Information

1.1.1 LTI-03

LTI-03 is being developed as a novel therapy for idiopathic pulmonary fibrosis (IPF) which is a progressive, fatal, age-associated lung disease with a median survival from diagnosis of 2-4 years and an incidence of 2.8 to 18 cases per 100,000 people per year in Europe and North America (Maher, Toby M., and Mary E. Strek. "Antifibrotic therapy for idiopathic pulmonary fibrosis: time to treat." *Respiratory research* 20.1 (2019): 1-9.

Martinez et al., 2017). The pathogenesis of IPF is characterized by apoptosis of alveolar epithelial type 2 cells (AEC2), proliferation and accumulation of activated myofibroblasts and fibrotic lung fibroblasts (FLF), deposition of extracellular matrix (ECM), and fibrosis, resulting in progressive dyspnea and loss of lung function (Lancaster, Lisa, et al. "Idiopathic pulmonary fibrosis: Physician and patient perspectives on the pathway to care from symptom recognition to diagnosis and disease burden." *Respirology* 27.1 (2022): 66-75.

Lederer DJ, Martinez , 2018). Idiopathic pulmonary fibrosis is rare in patients younger than 50 years old, with a median age at diagnosis of approximately 65 years. Most patients with IPF present with the advanced disease. The diagnosis is often delayed with the mean time between self-reported onset of symptoms and IPF diagnosis being 21.8 months for patients in the European IPF registry (Guenther et al., 2018) and the median time between self-reported onset of symptoms and IPF diagnosis being 13.6 months in the US IPF-PRO registry (Snyder et.al., 2020). Two anti-fibrotic drugs, nintedanib and pirfenidone, have been shown to reduce decline in lung function in patients with IPF and are approved for marketing in the United States (US) and the European Union (EU). Unfortunately, these two drugs provide only modest clinical benefit in IPF patients. These drugs slow the decline of lung function as measured by forced vital capacity (FVC). However, neither are curative and both are poorly tolerated leading to frequent drug discontinuations. Although the use of these drugs is increasing, a recent review of the time from diagnosis to treatment noted that only approximately 60% of IPF patients in Europe and the US were receiving nintedanib or pirfenidone (Maher et.al., 2019). Because these drugs are limited to only slowing the decline in lung function, there is a need to identify a more effective therapy for IPF or related interstitial lung disease (ILD).

1.1.1.1 Physical, Chemical, and Pharmaceutical Information

LTI-03 is a synthetic oligopeptide consisting of 7 natural L-amino acids which represent the mid portion of the caveolin-1 (Cav-1) scaffolding domain (CSD). LTI-03 exhibits limited solubility in water. It is formulated as a micronized dry powder in hard 2-piece hypromellose capsules. Encapsulated LTI-03 is self-administered- by subjects using a commercially available dry-powder inhaler device.

1.1.1.2 Non-clinical Background

Data from published studies and internal research suggest that LTI-03 targets aberrant signaling in lung fibrotic tissue that is characterized by decreased viability of alveolar epithelial cells (AECs) and increased proliferation and differentiation of fibroblasts. The restoration of homeostatic signaling by LTI-03 appears to increase AEC2 viability by decreasing senescence and apoptosis, while at the same time there appears to be an attenuation of a wide range of profibrotic signaling factors associated with less fibroblast proliferation and differentiation, and less ECM accumulation.

In *in vitro* studies of AECs isolated from the lungs of patients with IPF, LTI-03 lowered p53 and another apoptosis-associated protein, activated caspase-3, and restored levels of surfactant protein-C (SP-C), a key marker of AEC viability and function. LTI-03 had no effect on the expression of p53 in cultured AEC2s isolated from patients without pulmonary fibrosis. In IPF lung tissue explants cultured *ex vivo*, a dose dependent increase in lysotracker staining was observed for 7 days, suggesting that LTI-03 supported lung epithelial cell survival and possibly epithelial regeneration. In addition, *in silico* analyses using the Ingenuity Pathway Analyses software indicate that upregulation of Cav-1 may activate production of SP-C in AEC2 cells which is critical for lung homeostasis. As LTI-03 is derived from Cav-1, it would be expected to serve the same biological function.

Various tyrosine kinases play key roles in the pathologic activation of fibroblasts during fibrogenesis. Receptor tyrosine kinases, including Platelet-Derived Growth Factor Receptor, vascular Endothelial Growth Factor Receptor, Epidermal Growth Factor Receptor, and Janus tyrosine kinase, as well as non-receptor tyrosine kinases, such as c-Abl and Src kinases, stimulate the pathological synthesis and release of ECM proteins. In *in vitro* studies of primary FLFs, isolated from the lungs of patients with IPF or mice with established bleomycin (BLM)-induced pulmonary fibrosis, LTI-03 attenuated profibrotic signaling factors (p-PDGFRb, p-SMAD2/3), restored the expression of p53 to near normal levels, and inhibited the expression of fibrosis-associated proteins including type 1 collagen (Col-1), alpha-smooth muscle actin (α-SMA), fibronectin (FN), and tenascin-C (TN-C). Details on the LTI-03's effects *in vitro* and *in vivo* in non-clinical models of pulmonary fibrosis can be found in the Investigator Brochure (IB).

In summary, the potential mechanism of action of LTI-03 in lung fibrosis appears to be the restoration of homeostatic signaling associated with a novel, supportive, regenerative effect on lung epithelial cells and anti-fibrotic activity associated with attenuation of pro-fibrotic signaling in lung fibroblasts.

In pharmacokinetic (PK) studies carried out in healthy volunteers, dry powder inhaled LTI-03 appeared to be retained in the lung with slow or limited absorption into systemic circulation. As a result, systemic exposure is low and LTI-03 appears to be rapidly cleared from systemic circulation. In non-clinical PK studies of LTI-03, delivered as an aerosol or intratracheal bolus,

high lung tissue concentrations of LTI-03 were observed post-exposure while there was minimal LTI-03 absorption into the systemic circulation.

Pivotal 28-day Good Laboratory Practice (GLP) toxicity studies were performed in rats and dogs. These studies were designed such that the high dose levels would achieve at least a 50-fold margin over the proposed high clinical dose as recommended in International Council for Harmonisation (ICH)-M3(R2) guidelines. In Sprague Dawley rats, LTI-03 was well tolerated and there were no adverse test item effects reported at delivered doses of 18.4, 58.5 and 109.3 mg/kg/day. Histologic findings of goblet cell hypertrophy/hyperplasia of the nasal cavity, nasopharynx, trachea, and lungs observed in all dose levels and squamous metaplasia of the carina or nasopharynx seen at the mid and high doses were considered to be a nonspecific protective or adaptive response and were not adverse as they would not likely interfere with the functional or defensive capabilities of the respiratory tract. The no observed adverse effect level (NOAEL) in the rat was considered to be the achieved delivered dose of 109.3 mg/kg/day.

Similarly, in the dog, LTI-03 was well tolerated and no adverse effects were reported at achieved delivered doses of 8.9, 27.1 and 46.1 mg/kg/day. Histologic findings of minimal bronchiolar goblet-cell hypertrophy/ hyperplasia in the lungs of some high-dose animals and minimal to mild bronchioloalveolar duct hyperplasia in the lungs observed in all high-dose male dogs were considered a test item-related adaptive change, similar to the lesions observed in rats. The NOAEL in the dog was considered to be the achieved delivered dose of 46.1 mg/kg/day.

1.1.1.3 Clinical Background

LTI-03 was initially assessed in a Phase 1a study, LTI-03-1001: A Randomized, Double-Blind, Placebo-Controlled, Phase 1a, First-in-Man, Single Ascending Dose (SAD) and Multiple Ascending Dose (MAD) Safety, Tolerability and PK Study of a Cav-1-Scaffolding-Protein-Derived Peptide (LTI-03) in Healthy Adult Subjects. Twenty-four subjects were administered LTI-03 in 4 SAD cohorts at single doses of 10 mg, 40 mg, and 80 mg. At the 80 mg dose, subjects in 1 cohort were administered 4 20 mg capsules by inhalation and in a second cohort subjects at this 80 mg dose were administered 8 10 mg capsules by inhalation. Eight subjects in the combined SAD cohorts were administered placebo. In the SAD cohorts, 21 of the 24 subjects (88%) who were administered LTI-03 experienced treatment -emergent adverse events (TEAE), the most frequent of which were mild dry coughs that were considered to be related to LTI-03.

Twenty-nine subjects were administered LTI-03 by inhalation daily for up to 14 days in 5 MAD cohorts at doses of 2.5 mg once daily (QD), 5 mg QD, 5 mg twice daily (BID), 20 mg QD and 40 mg QD. The 2 higher dose MAD cohorts, 20 mg QD and 40 mg QD, were completed before the lower dose cohorts. The 3 lower doses were well tolerated. The most common TEAE in these 3 cohorts was a mild dry cough. During dosing in the 40 mg QD MAD cohort 1 subject developed severe TEAEs and two other subjects developed moderate TEAEs secondary to pulmonary airflow limitations (decreased forced expiratory volume 1 [FEV_1] and FEV_1/FVC ratios) that appeared to

be due to reversible airway obstruction. These events were considered to be dose limiting and related to LTI-03. There was no evidence that the TEAEs in the 3 subjects described previously were secondary to an allergic response. In the 20 mg QD MAD cohorts there were mild decreases in FEV_1 in two subjects that were not associated with TEAEs. These were not considered to be dose limiting. There were no serious adverse events (SAEs). All TEAEs were resolved 7 days after the treatment period. Further details can be found in the IB.

1.2 Scientific Rationale

Based on the non-clinical studies demonstrating the anti-fibrotic effects of LTI-03, the current study will investigate the initial safety and tolerability of inhaled LTI-03 in IPF patients diagnosed within 3 years of Screening without exposure to anti-fibrotic agents within 2 months of the Baseline bronchoscopy. Exploratory assessments will include evaluations of potential pharmacodynamic (PD) biomarkers. Newly diagnosed patients may be enrolled prior to beginning anti-fibrotic therapy. The potential 6-week delay in these patients in commencing therapy with pirfenidone or nintedanib is relatively short compared with the delay (approximately 14-22 months) from the onset of symptoms to the diagnosis of IPF in many patients (Guenther et al., 2018 and Snyder et.al., 2020). In addition to the time to diagnosis of IPF, there is frequently a delay in start of anti-fibrotic therapy post-diagnosis, with a recent physician reported survey reporting 4 months as a best case, to greater than 1-year from diagnosis to the first IPF prescription (Lancaster, et al., 2021).

1.2.1 Dose Rationale

Recent data from *in vitro* and *in vivo* pharmacology studies have been used to predict the nominal dose of LTI-03 dry powder that might be efficacious in patients. The predictions from the modeling suggest that a dose of between 1 and 10 mg LTI-03, inhaled by a patient using a commercially available dry-powder inhaler device, would result in a lung deposited dose likely to produce the desired pharmacological effect in the pulmonary compartment of the lung. The modelling approach, and the assumptions inherent to the modelling exercise, are discussed in detail in the IB.

Based on the modelling data, questionable or poor tolerability at 20 mg QD and greater in the MAD cohorts, and the good tolerability in lower dose MAD cohorts, the first cohort in this study will be dosed with LTI-03 2.5 mg administered BID (5 mg per day-low dose) for 14 days. If this dose is well tolerated the second cohort will be dosed at 5 mg administered BID (10 mg per day-high dose) for 14 days. Both doses were safe and well tolerated in healthy volunteers and will provide the best probability of finding an effective dose. Twice daily dosing is suggested by the limited non-clinical lung concentration time course data. Thus, it appears that efficacy will be more likely with BID dosing to afford more continuous lung exposure.

1.3 Potential Risks and Benefits

Dose limiting toxicities appeared in the highest dose MAD cohort with 40 mg of LTI-03 administered once daily by dry powder inhalation using 10 mg capsules. One subject developed severe TEAEs, and 2 subjects developed moderate TEAEs secondary to pulmonary airflow limitations (decreased FEV_1 and FEV_1/FVC ratios) that appeared to be due to reversible airway obstruction.

After consideration of recent *in vitro* and *in vivo* non-clinical and LTI-03 solubility studies, it is speculated that the 20 mg and 40 mg doses were supra-pharmacologic and may have resulted in a larger deposited insoluble fraction. According to the modelling data, these doses were approximately 20- to 40-fold in excess of a minimally efficacious dose, respectively. Such high predicted extracellular lining fluid (ELF) concentrations alone, or in combination with excess insoluble particulate, may have led to conducting airway irritation and/or obstruction resulting in a drop in FEV_1. The lower dose MAD cohorts used 2.5 mg capsules at doses of 2.5 mg QD and progressed to 2.5 mg BID (5 mg total daily dose) and ended with 5 mg BID (10 mg total daily dose). All three doses were well tolerated. These doses are adequate to support the proposed efficacious range of 1-10 mg and provide adequate safety margins above the rat and dog NOAELs based on body surface area (BSA) scaling (refer to the IB for further details).

IPF patients enrolled in this study will be administered study drug (LTI-03 or placebo) for 14 days. Given the short duration of treatment, subjects are not expected to experience a lung function benefit from administration of LTI-03 or placebo. In those newly diagnosed patients not previously exposed to antifibrotic agents, the potential 6-week delay in initiation of anti-fibrotic therapy following IPF diagnosis related to study participation is not expected to place these patients at undue risk as this delay is relative short compared with the common elapsed time from onset of symptoms to diagnosis in many patients, and from diagnosis to initiation of standard therapy. However, if the investigator determines that anti-fibrotic therapy should be started prior to study completion, study drug should be discontinued, and early termination procedures completed as per Section 7.4.

There is no nonclinical or clinical evidence that LTI-03 would adversely affect an immune response to COVID-19 infection or immunization. However, in the interests of caution, any vaccination (including vaccination against COVID-19) within 2 weeks of starting dosing (Day 1) and during the treatment period is prohibited.

2 Study Objectives And Endpoints

2.1 Objectives

2.1.1 Primary Objective

- To determine the safety and tolerability of inhaled LTI-03 in subjects diagnosed with IPF within 3 years of Screening.

2.1.2 Exploratory Objectives

- To assess the plasma PK of inhaled LTI-03 after the first dose and at steady state in subjects with IPF diagnosed within 3 years of Screening.
- To evaluate the PD biomarkers of inhaled LTI-03 in subjects diagnosed with IPF within 3 years of Screening.

2.2 Endpoints

2.2.1 Primary Endpoint

- Incidence of TEAEs.

2.2.2 Other Safety Endpoints

- Other safety endpoints include:
  - Physical examination findings
  - Vital signs
  - Electrocardiogram (ECG)
  - Clinical laboratory measurements
  - Spirometry
  - Leicester Cough Questionnaire
  - Cough Visual Analogue Scale

2.2.3 Exploratory Endpoints

- To determine the plasma peak and trough concentration of LTI-03 after the first dose and at steady state.
- Change from baseline over time for biomarkers related to the pathophysiology of IPF as outlined in Table 3.

3 STUDY DESIGN

This is a randomized, double-blind, placebo-controlled, multi-center, dose escalation, safety and tolerability study of LTI-03 or placebo administered by inhalation in subjects with IPF without =treatment with anti-fibrotic agents withing 2 months of the Baseline bronchoscopy. An IPF diagnosis will be confirmed by high-resolution computed tomography (HRCT) of chest or lung biopsy within 3 years prior to Screening. The study will contain 2 dose cohorts which will run sequentially. Subjects in the first, low-dose cohort will receive 2.5 mg of study drug (LTI-03 or placebo), administered twice daily approximately 10-12 hours apart (5 mg per day). If the 5 mg per day dose is well tolerated, a second, high-dose cohort will receive 5 mg of study drug (LTI-03 or placebo), administered twice daily approximately 10-12 hours apart (10 mg per day).

Eligible subjects will be randomized in a 3:1 ratio to either LTI-03 or placebo. Each cohort will have 9 subjects randomized to LTI-03 and 3 subjects randomized to placebo. Safety data will be reviewed on an ongoing basis by the Sponsor's medical officer and the Contract Research Organization (CRO)'s medical monitor. A review of safety data including but not limited to adverse events, laboratory assessments, vital signs, and spirometry will be conducted after all 12 subjects from Cohort 1 have completed Day 14 assessments. Enrollment in the second cohort will not begin until the Cohort 1 safety data has been reviewed and a dose decision made. A lower dose cohort may be added (2.5 mg administered once daily) or additional subjects (up to 12 subjects) may be added to the low-dose cohort (2.5 mg administered twice daily) if doses of 5 mg twice daily (10 mg per day) are not tolerated. In total, a maximum of approximately 24 subjects will be randomized in the study.

The Treatment Period will be 14 days, with subjects self-administering the study drug using the provided commercially available dry powder inhaler. All subjects will be trained on the proper methods of inhaler use and will have access to site personnel trained to assist subjects remotely if questions arise. Retraining may occur on Day 1 and 7, if required. During the Treatment Period, there will be on-site visits on Day 1, Day 7, and Day 14 and study assessments will occur at the investigational site on these days. The first dose of study drug will be administered at the investigational site and subjects will be monitored for at least 1 hour after dosing.

Screening assessments may take place over no more than 21 days prior to the Treatment Period. Screening assessments will include but not be limited to a complete physical examination including vital signs, ECG, spirometry, safety laboratory parameters, medical history, and concomitant medications. A Baseline bronchoscopy will be performed during the Screening Period in subjects who meet the Screening criteria and will include bronchoalveolar lavage (BAL) and/or bronchoabsorption (BA), and deep bronchial brushings.

Treatment Period will include but not be limited to physical examinations with vital signs, ECG, spirometry, clinical laboratory assessments, adverse event (AE) assessment, Leicester cough questionnaire, cough visual analogue scale (VAS), and concomitant medications. Bronchoscopies

with BAL and/or BA and deep bronchial brushings will also be performed on Day 14 approximately 2-3 hours after morning dosing.

Follow-up study assessments will be completed at Day 21 with all Day 14 assessments repeated except dosing, bronchoscopy, and blood collection for biomarkers and PK. Also, a complete physical examination will be replaced by a brief physical examination. The end of the study is defined as the date of the last visit of the last participant in the study.

4 STUDY SUBJECTS

4.1 Eligibility Criteria

The study subject population will consist of patients with IPF without treatment with anti-fibrotic agents within 2 months of the Baseline bronchoscopy.

4.1.1 Inclusion Criteria

Individuals must meet all the following inclusion criteria to be eligible for participation in this study.

1. Male or female subject of age 40 years or older.
2. Willing and able to provide written informed consent.
3. Diagnosis of IPF within 3 years of Screening as confirmed by HRCT of chest or lung biopsy as defined by ATS/ERS/JRS/ALAT guideline (Raghu G al, 2018).
4. Forced vital capacity (FVC) percent predicted ≥ 40%.
5. Diffusion capacity of the lungs for carbon monoxide (DLCO) percent predicted ≥ 30 and ≤ 80.
6. Forced expiratory volume 1 (FEV_1)/FVC ≥ 0.7.

4.1.2 Exclusion Criteria

Individuals who meet *any* of the following exclusion criteria will not be enrolled in this study.

1. Interstitial lung disease other than IPF.
2. Evidence of significant obstructive lung disease.
3. Current diagnosis of asthma.
4. Treatment with an approved or investigational anti-fibrotic therapy for IPF within 2 months of the Baseline bronchoscopy.
5. Use of N-acetyl cysteine or other supplements within 7 days prior to dosing and throughout the Treatment Period.
6. Inability to use study inhaler device appropriately.

7. Pulmonary exacerbation within 6 months prior to Screening.
8. Febrile illness within 7 days prior to dosing.
9. Participation in a clinical study or treatment with an investigational drug or device within 30 days of the Screening Visit (or 5 half-lives of the investigational agent, whichever is longer).
10. History or evidence at screening of significant renal impairment with eGFR < 30 mL/min.
11. History or evidence at screening of significant hepatic impairment with bilirubin > 3 mg/dL (> 51.3 µmol/L) and albumin < 2.8 g/dL (<28 g/L) and PT prolongation > 6 sec or INR > 2.3.
12. Serious or active medical or psychiatric condition which, in the opinion of the Investigator, may interfere with treatment, assessment, or compliance with the protocol.
13. Vaccination within 2 weeks of start of dosing (Day 1) and throughout the Treatment Period.
14. Subject has severe progressive or uncontrolled, clinically significant disease that in the judgment of the investigator or designee renders the subject unsuitable for the study.

Contraception and Pregnancy

15. Positive pregnancy test in female subjects of childbearing potential as defined below.
16. Female subjects who are lactating.
17. Females of childbearing potential (FOCBP) and men with partners of childbearing potential who do not agree to use an acceptable form of contraception for the duration of study treatment and for at least 90 days after the last dose of study drug. Male subjects who do not agree to refrain from donating sperm during this same period.

    These methods of contraception are acceptable:

    - Bilateral tubal ligation; male sterilization; hormonal contraceptives that inhibit ovulation; hormone-releasing intrauterine devices; and copper intrauterine devices.
    - True abstinence when this is in line with the preferred and usual lifestyle of the subject. Periodic abstinence (e.g., calendar, ovulation, symptothermal post-ovulation methods) and withdrawal are not acceptable methods of contraception.
    - Male sterilization (with appropriate post-vasectomy documentation of the absence of sperm in the ejaculate). For female subjects, the vasectomized male partner must be documented as the sole partner.

    Contraceptive requirements do not apply for subjects who are exclusively in same sex relationships. If a subject who is in a same sex relationship at the time of signing the Informed Consent Form (ICF) becomes engaged in a heterosexual relationship, they must agree to use contraception as described and as outlined in the protocol and ICF.

NOTE: Female subjects who are surgically sterile or post-menopausal for at least 12 months without any other underlying medical cause are not considered to be of childbearing potential.

5 STUDY DRUG

5.1 Formulation, Packaging and Labeling

LTI-03 is a Cav-1-scaffolding-protein-derived peptide and is an active study drug. LTI-03 and matching placebo are provided as inhalation powders for oral inhalation use. LTI-03 is an excipient-free, micronized dry powder that is placed into white opaque hypromellose two-piece capsules. The placebo is micronized lactose powder that is placed into white opaque hypromellose two-piece capsules and is identical in appearance to LTI-03. Individual capsules that contain 2.5 mg of study drug (LTI-03 or placebo) are not labelled.

5.2 Storage and Stability

Study drug (LTI-03 or placebo) is to be stored below 25°C and in the original package to protect from moisture.

5.3 Accountability

Bottles containing study drugs (LTI-03 or placebo) will be supplied by the Sponsor. Each bottle will be labeled with a unique packaging identification number.

The Investigator is responsible for ensuring adequate accountability of all used and unused study drug. The study site is responsible for maintaining drug accountability logs for tracking the administration of all study drug. Unused study drug may be returned to the Sponsor. Subjects will return used capsules and unused study drug to the investigational site during the Day 7 and Day 14 on-site visits.

5.4 Administration and Management of Study Drug

Study staff will receive training from the Sponsor on the proper administration of study drug.

All subjects will self-administer study drug using the provided commercially available dry-powder inhaler. Study staff will train all subjects on proper use of the inhaler. Subjects must demonstrate the ability to adequately inhale a minimum of 2 individual empty capsules using the inhaler device. Additional capsules may be used for training. Study staff will assist with training and administration of this test inhalation and will make a determination of qualification of the subject. Subject retraining may occur at Day 1 and Day 7, if required.

The first dose of study drug will be administered at the investigational site on Day 1 and subjects will be monitored for at least 1 hour after dosing. Study staff will monitor subjects' self-administration of study drug (most likely the morning dose) during on-site visits on Day 1, Day 7, and Day 14.

The first, low-dose cohort will receive 2.5 mg of study drug (LTI-03 or placebo), administered twice daily approximately 10-12 hours apart (5 mg per day). If the 5 mg per day dose is well tolerated, a second, high--dose cohort will receive 5 mg of study drug (LTI-03 or placebo), administered twice daily approximately 10-12 hours apart (10 mg per day).

As each capsule contains 2.5 mg of study drug (LTI-03 or placebo), two inhalations will be used i.e., using 2.5 mg capsule + 2.5 mg capsule to achieve a dose of 5 mg. Subjects should wait 1-2 minutes between these 2 inhalations and may drink a small amount of water (e.g., 15 mL to 30 mL) between the inhalations. These two inhalations should be administered within a 5-minute period.

As mentioned, in Section 3, a lower dose cohort may be added (2.5 mg administered once daily) or additional subjects may be added to the low-dose cohort (2.5 mg administered twice daily).

Table 1 Instructions on Multiple Administrations taken at a Time and for Missing the Dose

Study Drug	Dosing per Day	Instruction on Multiple Inhalations Taken at a Time	Instructions on Missing the Dose
**Low Dose (5 mg/day)**	5 mg/day achieved by inhalation of one 2.5 mg capsule, twice daily, approximately 10 to 12 hours apart	NA	If the morning dose is missed, subject should administer the dose by noon (12:00 pm). If the morning dose is not taken by noon, then it should be skipped, and evening dose should be taken at the regular time.<br>NOTE: the morning dose can be taken after noon during in-clinic visits if scheduling does not allow for a morning administration.
**High Dose (10 mg/day)**	10 mg/day achieved by inhalation of two 2.5 mg capsules, twice daily, approximately 10 to 12 hours apart	Subject will wait 1-2 minutes between multiple inhalations and may drink a small amount of water (e.g., 15 mL to 30 mL) between inhalations. Multiple inhalations should be administered within a 5-minute period.	

Abbreviations: NA = not applicable.

The complete details on administration of study drug will be provided to the site separately.

5.5 Method of Assignment to Treatment

The actual treatment given to individual subjects is determined by a randomization schedule. The associated treatment assignments giving details of individual subject treatment are available from the interactive response technology (IRT). The IRT will assign bottle numbers in a blinded manner. Assigned bottles will be dispensed by appropriately delegated study staff.

Each bottle of study drug will be assigned a unique packaging identification number. When a bottle of study drug is assigned to a subject, the packaging identification number will be recorded in the drug accountability records.

5.6 Concomitant and Prohibited Medications

All medications (including over-the-counter medicines, herbal treatments, supplements, and vitamins) administered within 30 days of the Screening Visit through Study Day 21 will be recorded on the Case Report Form (CRF), using generic names when possible.

If the subject had participated in any clinical study before entering this study, then treatment with the investigational product must have been discontinued within 30 days or 5 half-lives (whichever is longer) prior to Screening.

Other anti-fibrotic therapies for the treatment of IPF are prohibited two months prior to the Baseline bronchoscopy until 1 week after the last dose of the study drug. If the investigator determines that anti-fibrotic therapy should be started before study completion, study drug should be discontinued, and early termination procedures completed as per Section 7.4.

N-acetyl cysteine or quercetin is prohibited within 7 days of dosing (Day 1) and throughout the Treatment Period.

Vaccines are prohibited within 2 weeks of dosing day (Day 1) and throughout the Treatment Period.

6 Study Procedures

The procedures and assessments that are outlined in this section will be performed at the time points specified by study visit in the schedule of assessments (SOA) (see Appendix 1 and Appendix 2).

For subjects receiving multiple administrations and having time-sensitive post-dose assessments, "Time 0" is defined as start of the administration of the first capsule of study drug.

6.1 Informed Consent

Written informed consent must be provided by each subject prior to the initiation of any study-specific procedure or assessment that is not part of standard of care. Informed Consent Forms for enrolled subjects and for subjects who are not subsequently enrolled will be maintained at the study site.

All Screening evaluations must be completed and reviewed to confirm that subjects meet all the eligibility criteria before enrollment. The Investigator will maintain a Screening log to record details of all patients screened and to confirm eligibility or record reasons for Screening failure, as applicable.

6.2 Demographics and Medical History

Demographic data will include age, sex, and self-reported race and ethnicity.

All ongoing conditions, any environmental/work exposure history and relevant or significant medical history from the past 5 years (including all major hospitalizations and surgeries) will be recorded. Any neoplastic conditions, irrespective of the time of diagnosis, should be noted. Reproductive status and smoking history will be recorded.

6.3 Vital Signs

Vital signs will be obtained after subject has been sitting for at least 1 minute and will include blood pressure, heart rate, respiratory rate, body temperature and hemoglobin oxygen saturation (via pulse oximetry).

6.4 Physical Examination

A complete physical examination will include an evaluation of the head, eyes, ears, nose and throat, and the cardiovascular, dermatologic, musculoskeletal, respiratory, gastrointestinal, and neurologic systems. Any abnormality identified at baseline (Screening Physical Exam) will be recorded as medical history.

A brief physical examination will focus on systems affected by AEs and at a minimum should include the cardiovascular and respiratory systems.

Changes from baseline abnormalities should be recorded in subject notes. New or worsened clinically significant abnormalities should be recorded as TEAEs.

Height and weight will be recorded at Screening.

6.5 Clinical Laboratory Assessments

Samples taken from subjects will be sent to a central laboratory for analyses. Clinical laboratory assessments will be performed for variables provided in Table 2.

Table 2 Clinical Laboratory Assessments

**Hematology**	
red blood cell (RBC) count	red cell distribution width (RDW)
mean corpuscular hemoglobin concentration (MCHC)	hematocrit (Hct)
mean corpuscular hemoglobin (MCH)	hemoglobin (Hgb)
mean corpuscular volume (MCV)	white blood cell (WBC) count including differential count (lymphocytes, monocytes, neutrophils, eosinophils, basophils) and platelet count.
**Clinical chemistry**	
bilirubin (total and conjugated)	cholesterol (total, low density lipoprotein cholesterol [LDL-C], high density lipoprotein cholesterol [HDL-C])
Aspartate aminotransferase (AST)	glucose
alanine aminotransferase (ALT)	blood urea nitrogen (BUN)
alkaline phosphatase (ALP)	creatinine
gamma glutamyl transferase (GGT)	sodium
lactate dehydrogenase (LDH)	potassium
creatine kinase (CK)	chloride
protein	calcium
albumin	magnesium
	C-reactive protein (CRP)
**Coagulation**	
prothrombin time (PT)	
INR	
activated partial thromboplastin time (aPTT)	

Additional assessments may be added if clinically indicated and if there is no increase in required blood volume.

6.5.1 Pregnancy Test

Females of childbearing potential must have a negative urine or serum pregnancy test during Screening. A urine or serum pregnancy test will also be performed on study days as mentioned in SOA (see Appendix 1).

6.6 Electrocardiogram

A single standard 12-lead ECG will be performed. Lead placement should be as consistent as possible. ECG recordings must be performed after the subject has been resting in a supine position for at least 5 minutes.

ECG parameters to be evaluated include HR and the RR, QT, QRS, and PR intervals. In addition, Fridericia's formula should be used to calculate the QT interval corrected for heart rate (QTcF). ECG morphology statements will also be evaluated with particular attention paid to waveform morphology findings of: ST segment abnormal, T wave changes, abnormal U waves, atrioventricular (AV) block, arrhythmia (supraventricular, ventricular, atrial fibrillation/flutter, etc.), and others including infarction, ischemia, and hypertrophy. ECG abnormalities will be recorded as AEs only if they are considered to be clinically significant by the Investigator.

6.7 Spirometry

Spirometry to assess FEV1, FVC, FEV1/FVC and the percent predicted for each of the parameters will be performed during Screening, Day 1, Day 7, Day 14, and Day 21. DLCO will be assessed only at Screening. Percent predicted will be calculated based on height, age, and ethnicity. Spirometry will be performed according to the current American Thoracic Society (ATS) / European Respiratory Society (ERS) Task Force guidelines (Miller et al., 2005). Spirometry will be performed on Days 1, 7, and 14 pre-dose and post-dose at 30 and 60 minutes. Unscheduled spirometry may as be performed as needed for respiratory symptoms. If bronchospasm is suspected, spirometry may also be performed before and after a rescue beta-adrenergic agonist inhalation.

6.8 Inhaler Use Assessment

As part of study Screening, subjects must demonstrate the ability to adequately inhale a minimum of two individual empty capsules using the inhaler device. Additional capsules may be used for training. Study staff will assist with training and administration of this test inhalation and will make a determination of qualification of the subject. Retraining may occur on Day 1 and Day 7, if required.

6.9 Bronchoscopy with Bronchoalveolar Lavage and/or Bronchoabsorption and Deep Bronchial Brushing

A baseline bronchoscopy will be performed during the Screening Period and may be part of the IPF initial assessment of a recently diagnosed IPF subject who has provided informed consent for bronchoscopy. Bronchoalveolar lavage and/or BA, alternate methods of sampling airway lining fluid, and deep (small airway) bronchial brushings will be performed. Details of the BAL, BA, and brushing procedures are provided in a separate manual. Bronchoscopy with BAL and/or BA, and deep bronchial brushings will be repeated on Day 14 approximately 2-3 hours after the morning dose. Subjects who underwent BAL at the baseline bronchoscopy should have BAL performed at the Day 14 bronchoscopy. In a similar manner, BA should be performed at the Day 14 bronchoscopy in those subjects who underwent BA at the baseline bronchoscopy. Subjects will be instructed to wait to take their Day 14 dose until they arrive at their research clinic visit.

Study personnel performing BAL, BA and deep bronchial brushing will be documented as qualified to perform the procedures on the delegation log.

6.10 Leicester Cough Questionnaire

The Leicester Cough Questionnaire is a subject self-report, health related quality of life questionnaire (see Appendix 3) that will be collected as mentioned in the SOA (see Appendix 1).

6.11 Cough Visual Analogue Scale

The cough VAS is a 100 mm scale (see Appendix 4) on which subject will be asked to mark between 'no cough' and 'the worst cough severity'. This assessment will be collected as mentioned in the SOA (see Appendix 1)

6.12 Pharmacokinetic Sampling

Plasma and BAL samples for PK assessment will be collected as mentioned in SOA (see Appendix 1 and Appendix 2).

6.13 Exploratory Biomarker Sampling

Exploratory biomarkers will be assessed in peripheral blood cells (as feasible), platelet rich plasma, BALF, BA samples, and deep bronchial brushings as outlined in Table 3. Ribonucleic acid (RNA) transcription analysis from whole blood may be substituted for any assays using peripheral blood cells. Samples will be collected at the time of bronchoscopy during the Screening Period and Day 14 as shown in Appendix 1. Additional biomarkers may be added as new information is generated about the potential effects of CSD signaling.

Permission to collect these samples and their future analyses will be included in the informed consent.

Sample collection, processing and shipping instructions will be provided in a separate document (Laboratory Manual). Sample analysis will be performed in the Sponsor assigned laboratory.

Table 3 Exploratory Biomarkers by Sample Source and Assumed Indicator Function

Sample source / Indicator of	Epithelial damage / repair	Fibrosis	Inflammation	Thrombosis
**Peripheral blood cells** [b]	-	p-AKT[b], IL-11	CXCL7	-
**Platelet rich plasma (PRP)**	CYFRA 21-1, SP-D, CA-19-9, KL-6, sRAGE, Galectin 7	MMP-7, Tenascin C (TNC), Periostin, IL-11, MYDGF, MMP-2	CCL18, CXCL13, slCAM1, IL-11, sCD163, CXCL7	PAI-1

**BAL and/or BA[a]**	Galectin 7, SP-D, sRAGE	MYDGF, MMP-2, TNC, MMP-7, periostin, IL-11	CCL18, CXCL13, slCAM1, IL-11, sCD163, CXCL7	PAI-1
**Deep bronchial brushings**	-	p-SMAD2/3	-	-

Abbreviations: BAL = bronchoalveolar lavage. BA = bronchoabsorption
[a] LTI-03 levels will also be measured on BAL samples. LTI-03 will not be measured in BA samples
[b] Ribonucleic acid transcription analysis may be substituted for assays using peripheral blood cells.

6.14 Treatment, Subject, Study and Site Discontinuation

6.14.1 Premature Discontinuation of Study Drug

Subjects meeting any of the following criteria will discontinue study drug:

- Initiation of a prohibited concomitant medications (see Section 5.6).
- Unacceptable AE or failure to tolerate study drug.
- Decrease from Baseline in FEV1 of 15% associated with a decrease from Baseline in the FEV1/ FVC ratio.
- Changes in the subject's condition or development of an intercurrent illness or if subject contracts Corona virus disease-19 (COVID-19) infection that renders the subject unsuitable for further study drug in the judgment of the Investigator.
- Subject request to discontinue the study drug.
- Subject is lost to follow-up.
- Discretion of the Investigator or Sponsor.
- Termination of the study by the Sponsor.

The primary reason for the discontinuation of study drug should be documented.

Wherever possible, post-treatment follow-up should be completed for these subjects.

6.14.2 Subject Withdrawal of Consent

Subjects may withdraw their consent to participate in the study at any time and for any reason without prejudice to their future medical care by the physician at the institution.

In addition, the Investigator has the right to withdraw a subject from the study at any time. Reasons for withdrawal from the study may include, but are not limited to, the following:

- Subject withdrawal of consent.
- Study termination or site closure.

If a subject withdraws consent, the date and stated reason for consent withdrawal should be documented. No further follow-up will be done on these subjects, although study information collected up to the time of withdrawal of consent will be included in the clinical study report.

6.14.3 Replacement of Subjects

The Investigator and Sponsor may elect to replace subjects in a given cohort following subject discontinuation or withdrawal if the discontinuation or withdrawal occurs for reasons other than the safety or tolerability of the study drug. The Investigator will document the decision and rationale for subject replacement if this action is taken.

6.14.4 Study Discontinuation

The Sponsor has the right to terminate this study at any time. Reasons for terminating the study may include, but are not limited to, the following:

- The incidence or severity of AEs in this or other studies indicates a potential health hazard to subjects.
- Subject enrollment is unsatisfactory.

The Sponsor will notify the Investigator if the Sponsor decides to discontinue the study.

6.14.5 Site Discontinuation

The Sponsor has the right to close a site at any time. Reasons for closing a site may include, but are not limited to, the following:

- Excessively slow recruitment.
- Poor protocol adherence.
- Inaccurate or incomplete data recording.
- Non-compliance with the ICH guideline for GCP.

7 STUDY SCHEDULE

Written Institutional Review Board (IRB) / Independent Ethics Committee (IEC) approval of the protocol, informed consent and any additional subject information will be obtained prior to starting patient Screening and enrollment.

7.1 Screening Period (Day -21 to Day -1)

During Screening, a unique study-assigned subject number will be assigned to each subject who signs the informed consent for the study. Once subjects are in Screening or are enrolled in the study, they will only be identified by the assigned identification number. A subject may be re-screened once, providing that additional written informed consent is obtained.

The reason(s) why a subject is not eligible for the study will be collected.

The following procedures and assessments will be performed during the Screening Period to confirm eligibility.

- Obtain written informed consent
- Record demographics and medical history
- *Record prior and concomitant medications taken within 30 days prior to first dose of study drug (Day 1)
- Obtain vital signs (blood pressure, heart rate, respiratory rate, body temperature, and hemoglobin oxygen saturation via pulse oximetry)
- *Perform a complete physical examination (including documentation of height and weight)
- Assess ability to use inhaler
- Single 12-lead ECG
- Perform spirometry with DLCO
- Collect blood sample for the following:
    - Hematology
    - Clinical chemistry
    - Coagulation
    - *Biomarker assessments
- Collect urine or serum sample for pregnancy test, if applicable
- Confirm eligibility according to inclusion/exclusion criteria.
- *Leicester Cough Questionnaire and Cough VAS prior to bronchoscopy

- *Adverse event assessment
- Baseline bronchoscopy with BAL and/or BA and deep bronchial brushings for biomarkers will be performed during the Screening Period in those subjects who have provided informed consent. Prior to bronchoscopy, the assessments with * in Section 7.1 will be performed.

7.2 Treatment Period

7.2.1 Day 1

The first dose of study drug will be administered at the investigational site and subjects will be monitored for at least 1 hour after dosing. Study staff will provide retraining on inhaler use, if required.

Assessments and procedures performed prior to dosing include:

- Confirmation of eligibility
- Concomitant medication assessments
- Adverse event assessment
- Brief physical examination
- Vital signs
- Single 12-lead ECG
- Collect urine or serum sample for pregnancy test.
- Randomization
- Collect blood for the following:
    - Hematology
    - Clinical chemistry
    - PK (pre-dose)
- Leicester Cough Questionnaire and Cough VAS
- Spirometry
- Dispense study medication and inhaler kit and administer Study Drug

Assessments and procedures performed after dosing include:

- Adverse event assessment
- Vital signs
- Collection of blood sample for the PK at 5 minutes (+ 5 minutes) after dosing

- Spirometry at 30 minutes (± 10 minutes) and 1 hour (± 10 minutes) after dosing

7.2.2 Days 2-6

Subjects will self-administer study drug at home and will be contacted on Day 4 (± 2 days) by study staff to check on compliance and for potential AEs.

7.2.3 Day 7

Subjects will return to the clinic on Day 7 (± 1 day). Subjects will be instructed to hold the morning administration of study drug until subject arrives at their clinical visit. Study staff will note the time of the dose and provide retraining on inhaler use, if required and check on compliance.

Assessments and procedures performed before dosing during this visit include:

- Concomitant medication assessments
- Adverse event assessment
- Brief physical examination
- Vital signs
- Single 12-lead ECG
- Collect urine or serum sample for pregnancy test.
- Collect blood for the following:
    - Hematology
    - Clinical chemistry
    - PK (pre-dose)
- Leicester Cough Questionnaire and Cough VAS
- Spirometry
- Subject will return the used and unused study drug
- Dispense and administer Study Drug

Assessments and procedures performed after dosing include:

- Adverse event assessment
- Vital signs
- Collect blood for the following:
    - PK at 5 minutes (+ 5 minutes) after dosing

- Spirometry at 30 minutes (± 10 minutes) and 1 hour (± 10 minutes) after dosing

7.2.4 Days 8-13

Subjects will self-administer study drug at home and will be contacted on Day 10 (± 2 days) by study staff to check on compliance and for potential AEs.

7.2.5 Day 14

Subjects will return to the clinic on Day 14 (±1 day) and the follow-up bronchoscopy will be performed. Subjects will be instructed to hold the morning administration of study drug until arrival at their clinical visit. Study staff will note the time of the dose and check on compliance. Follow-up bronchoscopy should occur approximately 2-3 hours after morning dosing. The second study drug dose will not be administered on Day 14.

All assessments and procedures will be performed prior to bronchoscopy.

Assessments and procedures performed before dosing during this visit include:

- Concomitant medication assessments
- Adverse event assessment
- Complete physical examination
- Vital signs
- Single 12-lead ECG
- Collect urine or serum sample for pregnancy test.
- Collect blood for the following:
  - Hematology
  - Clinical chemistry
  - PK pre-dose
  - Biomarker assessments
- Leicester Cough Questionnaire and Cough VAS
- Spirometry
- Subject will return the used and unused study drug
- Administer Study Drug

Assessments and procedures performed after dosing include:

- Adverse event assessment

- Vital signs
- Collect blood for the following:
- PK at 5 minutes (+ 5 minutes) after dosing
- Spirometry at 30 minutes (± 10 minutes) and 1 hour (±10 minutes) after dosing
- Bronchoscopy with BAL and/or BA and deep bronchial brushings after all other Day 14 assessments are complete

7.3 Follow-up Period and Day 21 Visit

All subjects will be followed for the assessment of safety for 7 days (±2 days) after the last dose of study drug and will return to the clinic for an End of Study (EOS) visit 7 days (±2 days) after their last dose of study drug. Assessments and procedures at the EOS visit include:

- Concomitant medication assessments
- Adverse event assessment
- Brief physical examination
- Vital signs
- Single 12-lead ECG
- Collect urine or serum sample for pregnancy test.
- Collect blood for the following:
  - Hematology
  - Clinical chemistry
- Leicester Cough Questionnaire and Cough VAS
- Spirometry

7.4 Early Termination

If a subject discontinues study drug before Day 14, the Day 14 assessments (other than study drug dosing), where feasible should be performed in the clinic as close as possible to the day of discontinuation. The EOS assessments should be performed 7 days after the last dose of study drug (assuming the subject has not withdrawn consent).

8 ASSESSMENT OF SAFETY

8.1 Specification of Safety Parameters

Safety will be evaluated through continuous monitoring of AEs, physical examinations, vital signs, clinical laboratory measurements, spirometry, and ECGs.

8.2 Definitions of Adverse Events

8.2.1 Adverse Event

According to the ICH guideline for GCP, an AE is any untoward medical occurrence in a subject or clinical investigation subject administered a pharmaceutical product, and which does not necessarily have to have a causal relationship with the treatment. An AE can therefore be any of the following:

- Any unfavorable and unintended sign (including an abnormal laboratory finding), symptom, or disease temporally associated with the use of a medicinal product, whether or not considered related to the medicinal product.
- Any new disease or exacerbation of an existing disease (a worsening in the character, frequency, or severity of a known condition).
- Recurrence of an intermittent medical condition (e.g., headache) not present at baseline.
- Any deterioration in a laboratory value or other clinical test (e.g., ECG) that is associated with symptoms or leads to a change in study treatment or concomitant treatment or discontinuation from study drug.
- Adverse events that are related to a protocol-mandated intervention, including those that occur prior to assignment of study treatment (e.g., Screening invasive procedures such as biopsies).

8.2.1.1 Treatment-Emergent Adverse Events

In this study protocol, AEs which begin or increase in severity or frequency at or after the administration of first dose of study drug are to be considered as TEAEs. Safety analyses will focus on TEAEs.

8.2.2 Serious Adverse Event

An SAE is any AE that meets any of the following criteria:

- Is fatal (i.e., the AE actually causes or leads to death).
- Is life threatening (i.e., the AE, in the view of the Investigator, places the subject at immediate risk of death).

This does not include any AE that, had it occurred in a more severe form or was allowed to continue, might have caused death.

- Requires or prolongs inpatient hospitalization.
- Results in persistent or significant disability / incapacity (i.e., the AE results in substantial disruption of the subject's ability to conduct normal life functions).
- Is a congenital anomaly/birth defect in a neonate / infant born to a mother exposed to study drug.
- Is a significant medical event in the Investigator's judgment (e.g., may jeopardize the subject or may require medical / surgical intervention to prevent one of the outcomes listed above)

Examples of such medical events include allergic bronchospasm requiring intensive treatment in an emergency room (ER) or at home, blood dyscrasias or convulsions that do not result in inpatient hospitalization.

Severity and seriousness need to be independently assessed for each AE.

SAEs are required to be reported by the Investigator to the Sponsor immediately (i.e., no more than 24 hours after learning of the event) by submission of a SAE Notification Form.

8.3 Adverse Event Classification

8.3.1 Relationship to Investigational Product

The Investigator’s assessment of causality must be provided for all AEs (serious and non-serious). An Investigator’s causality assessment is the determination of whether there exists a reasonable possibility that the investigational product caused or contributed to an AE.

- Not related: The AE is clearly not related to the study drug, or the temporal relationship of the onset of the AE relative to administration of the product is not reasonable, or the AE can be explained by another cause such as an underlying medical condition or other concomitant medication, or the AE has no plausible relationship to study drug.
- Related: The AE is probably related to the study drug. The temporal relationship of the AE to administration of the product is reasonable and there is no other cause to explain the event. Adverse events should be classified as related if the Investigator feels there is evidence to suggest a causal relationship between the study drug and the AE.

8.3.2 Severity

All adverse Events will be assessed according to the National Cancer Institute (NCI) Common Terminology Criteria for Adverse Events (CTCAE), Version 5.

Table 4 will be used for assessing severity for AEs that are not specifically listed in the NCI CTCAE.

Table 4 Adverse Event Severity Grading Scale for Events not Specifically Listed in NCI CTCAE

Grade	Severity
1	Mild; asymptomatic or mild symptoms; clinical or diagnostic observations only; or intervention not indicated
2	Moderate; minimal, local, or non-invasive intervention indicated; or limiting age-appropriate instrumental activities of daily living [a]
3	Severe or medically significant, but not immediately life-threatening; hospitalization or prolongation of hospitalization indicated; disabling; or limiting self-care activities of daily living [b, c]
4	Life-threatening consequences or urgent intervention indicated [d]
5	Death related to adverse event [d]

a. Instrumental activities of daily living refer to preparing meals, shopping for groceries or clothes, using the telephone, managing money, etc.
b. Examples of self-care activities of daily living include bathing, dressing and undressing, feeding oneself, using the toilet, and taking medications, as performed by patients who are not bedridden.
c. If an event is assessed as a "significant medical event," it must be reported as a serious adverse event, per the definition of serious adverse event in Section 8.2.2.
d. Grade 4 and 5 events must be reported as serious adverse events, per the definition of serious adverse event in Section 8.2.2.

Reference source: Common Terminology Criteria for Adverse Events (CTCAE) version 5.0 published on November 27, 2017.

8.4 Collection and Reporting of Adverse Events

8.4.1 Initial Reporting of Adverse Events

Any event occurring prior to administration of the first dose of study drug will be recorded in the Medical History on eCRF unless the AE is serious and related to a protocol-mandated intervention as per Section 8.2.1. A pre-existing medical condition should be recorded as an AE only if the frequency, severity, or character of the condition worsens during the study.

All AEs from time of first dose through the EOS will be collected, regardless of relationship to study drug.

A clinically relevant deterioration in laboratory assessments or other clinical findings, as assessed by the Investigator, is considered an AE, and must be recorded on the AE CRF if it meets the reporting time frames above. In addition, an abnormal test finding will be classified as an AE if one or more of the following criteria are met:

- The test finding is accompanied by clinically significant symptoms.

- The test finding necessitates additional diagnostic evaluation(s) or medical / surgical intervention, including additional concomitant drug treatment or other therapy. Note: simply repeating a test finding, in the absence of any of the other listed criteria, does not constitute an AE.
- The test finding leads to a change in study drug dosing or discontinuation of subject participation in the clinical research study.
- The test finding is considered an AE by the Investigator.

Wherever possible, a specific disease or syndrome rather than individual associated signs and symptoms should be identified. However, if an observed or reported sign or symptom is not associated with a specific disease or syndrome, the individual sign or symptom should be recorded as a separate AE. Laboratory data are to be collected as stipulated in this protocol. Clinical syndromes associated with laboratory abnormalities are to be recorded as appropriate (e.g., diabetes mellitus rather than hyperglycemia).

8.4.2 Adverse Events Reporting Period

After informed consent has been obtained but prior to initiation of study drug, only AEs caused by a protocol-mandated intervention (e.g., invasive procedures, discontinuation of medications) should be reported.

After initiation of study drug, all TEAEs (regardless of causal assessment to study drug) will be recorded until one week after the final dose of study drug (Day 21 Visit or EOS).

8.4.3 Follow-up of Adverse Events

All AEs considered to be related to study drug will be followed until resolution or stabilization.

8.5 Collection and Reporting of Serious Adverse Events

8.5.1 Initial Serious Adverse Event Reports

All SAEs that occur up to and including EOS must be reported by the Investigator to the Sponsor within 24 hours of awareness of the event by submission of a SAE Notification Form. Investigators must report to the Sponsor any SAE, whether or not considered drug related, including those listed in the protocol or IB.

The initial report must contain at a minimum the protocol number, a subject study identifier code, subject's initials, subject's data of birth, an event term, the suspected product and an assessment of causality. An Investigator may be requested by the Sponsor to obtain specific follow-up information in an expedited fashion. This information may be more detailed than that captured on the AE CRF. In general, this will include a description of the SAE in sufficient detail to allow for a complete medical assessment of the case and independent determination of possible causality.

Information on other possible causes of the event, such as concomitant medications and illnesses must be provided. In the case of a subject death, a summary of available autopsy findings must be submitted as soon as possible to the Sponsor.

8.5.2 Follow-up of Serious Adverse Events

All SAEs should be followed up until resolution or stabilization. The timelines and procedure for follow-up reports are the same as those for the initial report.

New information regarding an SAE that becomes available after the submission of the initial SAE Notification Form must be reported by the Investigator to the Sponsor by completion of a SAE Follow-up Report Form or through other written documentation (e.g., laboratory tests, discharge summary, postmortem results). Follow-up reports and/or written documentation must be provided to the Sponsor within 24 hours of the Investigator's receipt of the information.

8.5.3 Reporting of Death

Death is an outcome of an SAE and not an SAE itself. When death is an outcome, the event(s) resulting in death should be reported (e.g., "pulmonary embolism" with a fatal outcome).

8.6 Post-Trial Adverse Events

Any AE that occurs outside of the protocol-specified observation period or after the end of the study but is considered to be caused by the investigational product must be reported to the Sponsor. Instructions for how to submit these AEs will be provided separately.

8.7 Pregnancy Reporting and Follow-up

Pregnancy in a female clinical trial subject is not an SAE, however, complications of such pregnancies, for example, spontaneous abortion, may qualify as an SAE and should be reported as an SAE even if they occur after the SAE reporting period has ended.

The Investigator must notify the Sponsor via telephone or e-mail within 24 hours of awareness of a pregnancy in a study subject and must complete the Pregnancy Notification Form and submit it to the Sponsor within 2 working days of being notified. If the female subject is pregnant, then subject will not receive any further doses of their assigned study drug. The pregnancy must be followed to determine outcome, including spontaneous or voluntary termination, details of birth, and the presence or absence of any birth defects, congenital abnormalities, or maternal and/or newborn complications. This follow-up should occur even if the intended duration of safety follow-up for the trial has ended. Information regarding the course of the pregnancy, including perinatal and postnatal offspring outcome up to 8 weeks of age should be reported as follow-up information on the Pregnancy Notification Form.

Male study subjects will be instructed to notify the Investigator if a female partner becomes pregnant during the study. The Investigator must notify the Sponsor within 24 hours via telephone or e-mail and must complete the Pregnancy Notification Form and submit it to the Sponsor within 2 working days of being notified. The Investigator should obtain informed consent from the subject's partner using an EC-approved ICF, allowing the Investigator to obtain information regarding the pregnancy and its outcome. If the subject's partner provides informed consent, the Investigator should follow the pregnancy until outcome as described above for female study subjects and report the follow-up information on the Pregnancy Notification Form.

8.8 Stopping Rules

Safety data will be reviewed on an ongoing basis by the Sponsor's Medical Officer and the CRO Medical Monitor (Section 3) who will monitor subject safety during study conduct and can make decisions regarding individual subject drug, group/cohort treatment, and/or subsequent dosing regimens. The grading of events can be used to help determine these decisions, taking into consideration the subject's baseline parameters and the possibility for differences based on demographics.

Potential safety signals that will trigger a meeting of the Sponsor and CRO medical personnel, and subsequent written recommendation about study conduct include the following:

- Any SAE considered to be related to study drug
- Any related clinical NCI CTCAE ≥ grade 3 (severe) AE
- Any unrelated similar SAE or clinical NCI CTCAE ≥ grade 3 (severe) AE in ≥ 2 subjects. For example, wheezing and chest tightness would be considered similar because they may both be related to airway obstruction.

As noted in Section 6.14.1 study drug will be discontinued in subjects with a decrease from Baseline in FEV1 of ≥ 15% associated with a decrease from Baseline in the FEV1/FVC ratio.

Related AEs or suspected adverse drug reactions means that a causal relationship between a medicinal product and an AE is at least a reasonable possibility, i.e., there is evidence to suggest a causal relationship.

When a potential safety signal as defined above occurs, enrollment will be halted pending review by the Sponsor Medical Officer and CRO Medical Monitor and study treatment will be stopped in the individual experiencing the safety signal AE. All potential safety signals will be reviewed within 7 days by the Sponsor Medical Officer and CRO Medical Monitor that could recommend:

- Re-start subject enrollment. A protocol amendment summarizing available data and justification will be required to continue enrollment at the dose level associated with a stopping rule or any higher dose.

- Add a new lower dose cohort
- Add more subjects to a cohort without dose limiting toxicities
- Continued pausing of enrollment
- Permanently discontinuing enrollment into the study

8.9 Emergency Unblinding

The blind may be broken for an individual subject at the discretion of the Investigator if knowledge of the type of study drug administered is required to guide appropriate treatment decisions. The Sponsor’s Medical Officer should be notified within 24 hours of the Investigator’s decision to unblind.

8.10 Emergency Sponsor Contact

In a medical emergency (i.e., an event that requires immediate attention regarding operation of the clinical study and/or the use of investigational study drug), study site staff will apply appropriate medical intervention according to current standards of care and contact the Sponsor’s Medical Officer.

9 Statistical Considerations

9.1 Statistical and Analytical Plans

A statistical analysis plan (SAP) will be created, which will provide a more technical and detailed description of the proposed data analysis methods and procedures.

The results of the final analysis, as well as any deviations from planned statistical analyses, will be presented in the clinical study report.

9.1.1 Randomization Procedures

Subjects will be randomized to receive LTI-03: placebo in sequential cohorts, as per the study design. Randomization codes and unique subject identification numbers will be generated by the IVRS. All study staff will remain blinded to study drug assignment.

9.2 Study Hypotheses

The primary endpoint of the study is safety. No formal statistical hypotheses will be tested.

9.3 Analysis Set

The populations for analysis will include at a minimum a Safety Analysis Set. Other potential analysis sets will be defined in the SAP.

9.3.1 Safety Analysis Set

The Safety Analysis Set will consist of all subjects randomized to study drug and received at least 1 dose of study drug.

9.4 Statistical Methods and Analysis

9.4.1 General Approach

All descriptive statistical analyses will be performed using the most recently released and available Statistical Analysis System (SAS) statistical software (version 9.4 or higher), unless otherwise noted. For categorical variables, the number and percent of each category within a parameter will be presented. For continuous variables, the sample size (n), mean, median, and standard deviation, as well as the minimum and maximum values, will be presented. Missing data will not be imputed unless otherwise stated. Subjects on Placebo from all cohorts will be pooled to form a combined placebo group for analysis.

9.4.2 Demographics and Other Baseline Characteristics

Demographics and other baseline disease characteristics will be summarized by treatment and\or cohort.

9.4.3 Concomitant Medications

Concomitant medications will be coded using the latest version of the World Health Organization Drug Dictionary (WHO-DD).

9.4.4 Primary Endpoint

The primary endpoint of the study is incidence of TEAEs.

Adverse events will be graded according to National Cancer Institute (NCI) Common Terminology Criteria for Adverse Events (CTCAE), Version 5.

Adverse events will be coded by preferred term and system organ class using the current version of Medical Dictionary for Regulatory Activities (MedDRA®) and summary tables for all AEs will be generated for the Safety Population. Incidence rates and percentages will be summarized for each preferred term and system organ class. Additional summary tables will be generated for the following population subsets: subjects with SAEs, subjects with related AEs, subjects with severe (Grade 3 or 4) AEs, and subjects who discontinue study drug due to AEs. Adverse events will be summarized by treatment and cohort.

9.4.5 Other Safety Endpoints

Other safety endpoints are based on clinical evaluations, changes in clinical laboratory tests, vital signs, ECGs and spirometry.

9.4.5.1 Clinical Evaluations

Clinical evaluations used for the assessment of the safety will include physical examination findings, vital signs, spirometry, and ECG results.

Clinically significant changes in vital signs and new findings on physical examination will be recorded as AEs. Incidences of subjects with changes from normal physical examination findings at baseline to abnormal during the study may be generated.

Information on ECGs and spirometry will be presented in listings. Summary of mean / median changes in ECG intervals and mean / median changes in spirometry parameters may be generated on a data-driven basis. The incidence of subjects with changes from normal findings at baseline to abnormal during the study will be generated as appropriate.

9.4.5.2 Laboratory Assessments

All hematology, clinical chemistry and coagulation results will be listed by subject for each assessment and descriptive statistics may be tabulated for select criteria. Changes from baseline may be summarized by treatment.

9.4.6 Analysis of Exploratory Endpoints

9.4.6.1 Biomarkers

Details of the biomarker analysis will be provided in the SAP.

9.4.6.2 Pharmacokinetic

Details of the PK analysis will be provided in the SAP.

9.4.7 Planned Interim Analyses

No formal interim analyses are planned for the study. Cohort 1 biomarker data may be analyzed before final database lock without changing the overall study conduct.

9.5 Sample Size Determination

Approximately 24 subjects are planned in 2 cohorts. Each cohort will include 12 subjects (9 on LTI-03 and 3 on placebo). Selection of sample size is based on prior experience to ensure that the safety and tolerability of LTI-03 will be adequately assessed while minimizing unnecessary subject exposure. With 18 subjects receiving LTI-03 there is a 60% chance of detecting an AE with a true incidence rate of 5% and an 85% chance of detecting a more common AE with a true incidence rate of 10%.

10 Data Handling and Record Keeping

10.1 Study Files and Subject Source Documents

The Investigator must maintain adequate and accurate records to enable the conduct of the study to be fully documented and the study data to be subsequently verified. These documents include the subject's clinical source documents and Investigator's Study Files.

Subject clinical source documents may include, but are not limited to, hospital / clinic records, physician's and nurse's notes, appointment book, original laboratory reports, spirometry, ECG, radiograph, pathology and special assessment reports and consultant letters.

The Investigator will ensure the Study Files are maintained, including the CRFs and query forms, protocol/amendments, EC and regulatory approvals with associated correspondence, signed ICFs, study treatment records, staff curriculum vitae and authorization forms, all correspondence and other appropriate documents. Such data shall be secured in order to prevent loss.

The Investigator will allow personnel authorized by the Sponsor access to all study data at any time.

10.2 Data Collection Methods

Data collection is the responsibility of the study staff at the site under the supervision of the site Investigator. The Investigator is responsible for ensuring the accuracy, completeness, legibility, and timeliness of the data reported. A CRF must be completed for each subject who signs informed consent, regardless of the duration of their trial participation. The reason(s) why subjects are not enrolled will be tracked for any subject who signs an ICF.

All source documents should be completed in a neat, legible manner to ensure accurate interpretation of data. When making changes or corrections, cross out the original entry with a single line, and initial and date the change. Do not erase, overwrite, or use correction fluid or tape on the original. Documents (including laboratory reports, hospital records subsequent to SAEs, etc.) transmitted to the CRO or the Sponsor should include the study-assigned subject number but should not include the subject's name in order to ensure confidentiality.

CRFs will be provided for recording data for each subject enrolled in the study. Data reported in the CRF derived from source documents should be consistent with the source documents or the discrepancies should be explained and maintained in the subject's official electronic study record. The Investigator should consult the eCRF Completion Guidelines for comprehensive instructions for completing the CRFs.

Clinical data (including AEs, concomitant medications, and expected adverse reactions data) and clinical laboratory data will be entered into the data capture system. The data system includes password protection and internal quality checks, such as automatic range checks, to identify data

that appear inconsistent, incomplete, or inaccurate. Clinical data will be entered directly from the source documents.

10.3 Retention of Records

All clinical study documents must be retained by the Investigator until at least two years after the last approval of a marketing application in an ICH region and until there are no pending or contemplated marketing applications in an ICH region; or, if no application is filed or if the application is not approved for such indication, until two years after the investigation is discontinued and regulatory authorities have been notified. Investigators may need to retain documents longer if required by applicable regulatory requirements or if requested by the Sponsor. The Investigator must notify the Sponsor prior to destroying any clinical study records. Should the Investigator wish to assign the study records to another party or move them to another location, the Sponsor must be notified in advance. It is the responsibility of the Sponsor to inform the Investigator when these documents no longer need to be retained.

If the Investigator cannot guarantee the archiving requirement at the study site for any or all of the documents, special arrangements must be made between the Investigator and the Sponsor to securely store the documents in an off-site storage location so that documents can be returned to the Investigator in case of a regulatory audit. Where source documents are required for the continued care of the subject, appropriate copies should be made for off-site storage.

10.4 Protocol Deviations

The Investigator and the study site staff are responsible for ensuring the study is conducted in accordance with the schedule of procedures and assessments described in this protocol and in accordance with GCP. The site must use continuous vigilance to identify and report deviations within 24 hours of knowledge of their occurrence. CRO Standard Operating Procedures (SOPs) will be followed for the reporting of study deviations.

Intentional deviations from the protocol shall not be made without discussion with the Sponsor except in a medical emergency, when the intent is to reduce immediate risk to the subject. In such cases, the Sponsor, CRO, the EC and regulatory authorities, as appropriate, should be notified, in accordance with local requirements. In all other cases, the nature of the deviation, the justification for the deviation, and prior written approval of the Sponsor must be documented.

Changes to the protocol may be made only when a written substantial protocol amendment has been approved by the Sponsor and submitted to the EC and applicable regulatory agencies in accordance with local requirements. Appropriate approval(s) must be obtained before changes can be implemented.

11 Quality Control and Quality Assurance

Quality Control procedures will be implemented beginning with the data entry system, and data quality control checks will be run on the database. Any missing data or data anomalies will be communicated to the site(s) for clarification / resolution.

The CRO is responsible for ensuring the proper conduct of the study with regard to protocol adherence and validity of data recorded in the CRFs by assigning clinical research associates (CRAs) to the site. The CRA is responsible for reviewing the CRFs at regular intervals throughout the study, verifying adherence to the protocol, assuring completeness, consistency and accuracy of the data, reviewing study files and drug accountability. The data will be verified against the original medical records as part of source document verification to ensure validity of the data as outlined in a separate Monitoring Plan. The Investigator's responsibility is to ensure that any issues detected in the course of a monitoring visit are resolved.

To ensure compliance with GCP and all applicable regulatory requirements, a quality assurance audit may be conducted by the Sponsor or the Sponsor's designee. Regulatory agencies may also conduct a regulatory inspection of this study. Such audits / inspections can occur at any time during or after completion of the study. If an audit or inspection occurs, the Investigator and institution agree to allow the auditor / inspector direct access to all relevant documents and to allocate the Investigator and the time of Investigator's staff to the auditor / inspector to discuss findings and any relevant issues.

The Investigator and study staff are responsible for maintaining a comprehensive and accurate filing system of all study-related documentation that will be suitable for inspection at any time by the Sponsor, its designees, and/or regulatory agencies. In signing this protocol, the Investigator understands and agrees to give access to the necessary documentation and files.

12 ETHICS / PROTECTION OF HUMAN SUBJECTS

This section of the protocol is subject to the terms of the Clinical Trial Agreement between the Sponsor and the study center. In the event of a discrepancy between the Clinical Trial Agreement and this protocol, the terms of the Clinical Trial Agreement shall control.

12.1 Ethical Conduct of the Study

This study will be conducted in compliance with the ICH GCP guidelines, US regulations for the ethical conduct of clinical studies under 21 Code of Federal Regulations (CFR) Parts 50, 56 and 312, the Declaration of Helsinki, and with ICH guidelines regarding scientific integrity (E4, E8, E9 and E10), or national laws, if applicable. This study will also adhere to all Medicines and Healthcare Products Regulatory Agency (MHRA), state and local regulatory requirements and requirements for data protection.

12.2 Ethics Committee Review

Before trial initiation, the Investigator and institution must have written and dated approval from an accredited IRB/IEC for the study protocol, written ICF, subject recruitment procedures (e.g., advertisements), and any written information to be provided to subjects. Any amendment to the protocol will require review and approval by the IRB/IEC before the changes are implemented to the study. All changes to the ICF will be IRB/IEC approved; a determination will be made regarding whether previously consented subjects need to be re-consented.

Appropriate reports on the progress of the study will be made by the Investigator to the IRB/IEC and the Sponsor in accordance with applicable regulations. All correspondence with the IRB/IEC should be retained in the Investigator's Study File. Copies of IRB/IEC approvals should be forwarded to the Sponsor or its designee.

12.3 Informed Consent Process

The ICF must be approved by the IRB/IEC prior to being presented to potential study subjects. Any changes to the ICF must be approved by the Sponsor

Subjects may agree to participate in the clinical trial only after the risks and possible benefits of their participation have been explained and extensively discussed. The Investigator or designee will explain the purposes, procedures, and potential risks of the research study in terms suited to their comprehension, as well as their rights as a research subject. Subjects will have the opportunity to carefully review the written ICF, discuss the study with their surrogates, and ask questions prior to signing. Written informed consent must be obtained from each study subject or the subject's legally acceptable representative prior to conducting any study-related procedures.

The Investigator must use the most current EC-approved ICF for documenting written consent. Each informed consent will be appropriately signed and dated by the subject or the subject's legally

acceptable representative and by the person obtaining consent. The site must retain the original signed ICF and provide a copy to the subject.

12.4 Confidentiality of Information

Individual subject medical information obtained as a result of this study is considered confidential. The Investigator and the study center will adhere to all applicable laws relating to the protection of subject information. To assure that subject confidentiality is maintained, subject data will be identified only by a study-assigned subject number on any Sponsor forms, reports, publications, or in any other disclosures, except where required by law.

All personnel working for, or representing, the Sponsor and CRO will handle subject data in a confidential manner in accordance with applicable regulations governing clinical research. Subject records will be inspected only in connection with this research project. Information generated as a result of a subject's participation in this study may be disclosed to third parties for research, regulatory, and other purposes in any country as determined by the Sponsor. However, subjects will not be individually identified but will be referred to by the study-assigned subject number.

12.5 Future Use of Stored Specimens

Data collected for this study will be analyzed and stored at the CRO. After the study is completed, the de-identified, archived data will be transmitted to and stored at the Sponsor's facility or a delegated contract long-term storage vendor, for use by the Sponsor and its research collaborators.

With the subject's approval and as approved by the IRB/IEC, de-identified biological samples will be stored at the Central Laboratory and then a long-term storage vendor. These samples could be used for future research performed by the Sponsor or its collaborators.

12.6 Publication Policy

Publication and/or disclosure of information or data related to this protocol is subject to and governed by the Clinical Trial Agreement between the Sponsor and the clinical site to which the Investigator is a signatory.

After conclusion of the study, Investigators in this study may make oral presentations of study results or publish such results in scientific journals or other scholarly media without prior written approval from the Sponsor, only after the following conditions have been met:

- The results of the study in their entirety have been publicly disclosed by or with the consent of the Sponsor in an abstract, manuscript or presentation forum;
- The Investigator has complied with the terms of the Clinical Trial Agreement and all requests from the Sponsor to delete any references to its confidential information (other than study results);

- The study has been completed for at least two years.

13 References

Adams TS, Schupp JC, Poli S, et al. Single-cell RNA-seq reveals ectopic and aberrant lung-resident cell populations in idiopathic pulmonary fibrosis Sci Adv. 2020 Jul 8;6(28):eaba1983. doi: 10.1126/sciadv.aba1983. eCollection 2020 Jul.

Birring SS, Prudon B, Carr AJ, et al. Development of a symptom specific health status measure for patients with chronic cough: Leicester Cough Questionnaire (LCQ). Thorax 2003;58:339–343. http://dx.doi.org/10.1136/thorax.58.4.339.

Common Terminology Criteria for Adverse Events (CTCAE) version 5.0 published on November 27, 2017.

Guenther A, Krauss E, Tello S, et al. The European IPF registry (eurIPFreg): baseline characteristics and survival of patients with idiopathic pulmonary fibrosis. Respir Res 2018;19(1):141. DOI: 10.1186/s12931-018-0845-5.

Lancaster, Lisa, et al. "Idiopathic pulmonary fibrosis: Physician and patient perspectives on the pathway to care from symptom recognition to diagnosis and disease burden." *Respirology* 27.1 (2022): 66-75.

Lederer DJ, Martinez FJ. Idiopathic pulmonary fibrosis. N Engl J Med. 2018;378:1811–23. doi:10.1056/NEJMra1705751.

Maher, Toby M., and Mary E. Strek. "Antifibrotic therapy for idiopathic pulmonary fibrosis: time to treat." *Respiratory research* 20.1 (2019): 1-9.

Martinez FJ, Collard HR, Pardo A, Raghu G, et al. Idiopathic pulmonary fibrosis. Nat Rev Dis Prim. 2017;3:17074. doi:10.1038/nrdp.2017.74.

Miller MR, Hankinson J, Brusasco V, et al. Standardisation of spirometry. Eur Respir J. 2005;26:319-38.

Raghu G, Jardin MR, Myers JL., et al. Diagnosis of idiopathic pulmonary fibrosis. An official ATS/ERS/JRS/ALAT Clinical practice guideline. Am. J. Respir. Crit. Care Med. 2018;198:44-68.

Snyder LD, Mosher C, Holtze CH, et al. Time to diagnosis of idiopathic pulmonary fibrosis in the IPF-PRO Registry. BMJ Open Respir Res 2020;7(1). DOI: 10.1136/bmjresp-2020-000567.

Spinou A and Birring S. An Update on Measurement and Monitoring of Cough: What are the Important Study Endpoints? J Thorac Dis. 2014 Oct; 6(Suppl 7): S728–S734. Doi: 10.3978/J.Issn.2072-1439.2014.10.08

14 APPENDICES

14.1 Appendix 1 Schedule of Assessments

Table 5 Schedule of Assessments

	**Screening Period**	**Treatment Period**[1]			**Follow-Up**	**Early Termination**[2]
	**(Day -21 to Day -1)**	**Day 1**	**Day 7** (± 1 day)	**Day 14** (± 1 day)	**Day 21** (± 2 days)	
Informed consent	x					
Inclusion/Exclusion Criteria	x	x				
Demographics, Medical History	x					
Prior medications	x					
Concomitant medications	→	→	→	→	→	→
Complete physical examination[3]	x			x		x
Brief physical examination[4]		x	x		x	
Vital Signs	x	x	x	x	x	x
ECG (12-Lead ECG), single	x	x	x	x	x	x
Bronchoscopy with BAL and/or BA and deep bronchial brushings biopsy	x			x		x
Blood for biomarker assessments[5]	x			x		x
Pregnancy test[6]	x	x	x	x	x	x
Safety Laboratory parameters[7]	x	x	x	x	x	x
Randomization		x				
Blood collection for PK[8]		x	x	x		
Assessment of ability to use inhaler	x					
Leicester Cough Questionnaire & Cough VAS	x	x	x	x	x	x
Spirometry[9]	x	x	x	x	x	x
Dosing[10]		x	x	x		
Subjects returning unused study drug			x	x		
Adverse Events	x	→	→	→	→	→

Abbreviations: BAL = bronchoalveolar lavage; BA = bronchoabsorption; DLCO = diffusion capacity of the lungs for carbon monoxide; ECG = electrocardiogram; PK = pharmacokinetics; VAS = visual analogue scale

Note:

1. On Days 2 to 6 and Days 8 to 13, subjects will self-administer study drug at home and will be contacted on Day 4 (± 2 days) and Day 10 (± 2 days) by study staff to check on compliance and for potential adverse events. Day 1, 7, and 14 will be clinic visit. Study staff will monitor subjects self-administrating study drug (most likely the morning dose) on Day 1, Day 7, and Day 14.
2. If a subject discontinues study drug before Day 14, the Day 14 assessments (other than study drug dosing) where feasible should be performed in the clinic as close as possible to the day of discontinuation. The EOS assessments should be performed 7 days after the last dose of study drug (assuming the subject has not withdrawn consent).
3. A complete physical examination will include an evaluation of the head, eyes, ears, nose and throat, and the cardiovascular, dermatologic, musculoskeletal, respiratory, gastrointestinal, and neurologic systems. Any abnormality identified at baseline (Screening Day) will be recorded as medical history.
4. A brief physical examination will focus on systems affected by AEs and at a minimum should include the cardiovascular and respiratory systems.
5. Biomarker assessment will be for peripheral blood cells, platelet rich plasma, bronchoalveolar lavage fluid (BALF), BA samples, and deep bronchial brushings.
6. Serum or urine test required for any FOCBP, defined as pre-menopausal or < 12 months of amenorrhea post-menopause or no history of surgical sterilization
7. Safety laboratory assessments include hematology, chemistries, and coagulation. Coagulation will be performed only at Screening.
8. Blood for PK will be collected pre-dose (trough) and +5 minutes after dosing.
9. Spirometry: DLCO on Screening only. On treatment days (Day 1, 7, 14), it will be done before dosing and post dose at 30 and 60 minutes.
10. Study staff will monitor subjects self-administrating study drug (most likely the morning dose) on Day 1, Day 7, and Day 14. On Day 1, the first dose of study drug will be administered at the investigational site and subjects will be monitored for at least 1 hour after dosing. Study drug is dispensed on Day 1 and Day 7.

14.2 Appendix 2 Visit and Procedure Windows

Procedure	Visit	Window
Pharmacokinetics	Day 1	5 minutes + 5 minutes post-dose
	Day 7	5 minutes + 5 minutes post-dose
	Day 14	5 minutes + 5 minutes post-dose
Spirometry	Day 1	30 minutes + 10 minutes post-dose and 60 minutes + 10 minutes post-dose
	Day 7	30 minutes + 10 minutes post-dose and 60 minutes + 10 minutes post-dose
	Day 14	30 minutes + 10 minutes post-dose and 60 minutes + 10 minutes post-dose

14.3 Appendix 3 Leicester Cough Questionnaire

This questionnaire is designed to assess the impact of cough on various aspects of your life. Read each question carefully and answer by CIRCLING the response that best applies to you. Please answer ALL questions, as honestly as you can.

1. In the last 2 weeks, have you had chest or stomach pains as a result of your cough?

1	2	3	4	5	6	7
All of the time	Most of the time	A good bit of the time	Some of the time	A little of the time	Hardly any of the time	None of the time

2. In the last 2 weeks, have you been bothered by sputum (phlegm) production when you cough?

1	2	3	4	5	6	7
Every time	Most times	Several times	Some times	Occasionally	Rarely	Never

3. In the last 2 weeks, have you been tired because of your cough?

1	2	3	4	5	6	7
All of the time	Most of the time	A good bit of the time	Some of the time	A little of the time	Hardly any of the time	None of the time

4. In the last 2 weeks, have you felt in control of your cough?

1	2	3	4	5	6	7
None of the time	Hardly any of the time	A little of the time	Some of the time	A good bit of the time	Most of the time	All of the time

5. How often during the last 2 weeks have you felt embarrassed by your coughing?

1	2	3	4	5	6	7
All of the time	Most of the time	A good bit of the time	Some of the time	A little of the time	Hardly any of the time	None of the time

6. In the last 2 weeks, my cough has made me feel anxious

1	2	3	4	5	6	7
All of the time	Most of the time	A good bit of the time	Some of the time	A little of the time	Hardly any of the time	None of the time

7. In the last 2 weeks, my cough has interfered with my job, or other daily tasks

1	2	3	4	5	6	7
All of the time	Most of the time	A good bit of the time	Some of the time	A little of the time	Hardly any of the time	None of the time

8. In the last 2 weeks, I felt that my cough interfered with the overall enjoyment of my life

1	2	3	4	5	6	7
All of the time	Most of the time	A good bit of the time	Some of the time	A little of the time	Hardly any of the time	None of the time

9. In the last 2 weeks, exposure to paints or fumes has made me cough

1	2	3	4	5	6	7
All of the time	Most of the time	A good bit of the time	Some of the time	A little of the time	Hardly any of the time	None of the time

10. In the last 2 weeks, has your cough disturbed your sleep?

1	2	3	4	5	6	7
All of the time	Most of the time	A good bit of the time	Some of the time	A little of the time	Hardly any of the time	None of the time

11. In the last 2 weeks, how many times a day have you had coughing bouts?

1	2	3	4	5	6	7
All of the time (continuously)	Most times during the day	Several times during the day	Some times during the day	Occasionally through the day	Rarely	None

12. In the last 2 weeks, my cough has made me feel frustrated

1	2	3	4	5	6	7
All of the time	Most of the time	A good bit of the time	Some of the time	A little of the time	Hardly any of the time	None of the time

13. In the last 2 weeks, my cough has made me feel fed up

1	2	3	4	5	6	7
All of the time	Most of the time	A good bit of the time	Some of the time	A little of the time	Hardly any of the time	None of the time

14. In the last 2 weeks, have you suffered from a hoarse voice as a result of your cough?

1	2	3	4	5	6	7
All of the time	Most of the time	A good bit of the time	Some of the time	A little of the time	Hardly any of the time	None of the time

15. In the last 2 weeks, have you had a lot of energy?

1	2	3	4	5	6	7
None of the time	Hardly any of the time	A little of the time	Some of the time	A good bit of the time	Most of the time	All of the time

16. In the last 2 weeks, have you worried that your cough may indicate serious illness?

1	2	3	4	5	6	7
All of the time	Most of the time	A good bit of the time	Some of the time	A little of the time	Hardly any of the time	None of the time

17. In the last 2 weeks, have you been concerned that other people think something is wrong with you, because of your cough?

1	2	3	4	5	6	7
All of the time	Most of the time	A good bit of the time	Some of the time	A little of the time	Hardly any of the time	None of the time

18. In the last 2 weeks, my cough has interrupted conversation or telephone calls

1	2	3	4	5	6	7
Every time	Most times	A good bit of the time	Some of the time	A little of the time	Hardly any of the time	None of the time

19. In the last 2 weeks, I feel that my cough has annoyed my partner, family or friends

1	2	3	4	5	6	7
Every time I cough	Most times when I cough	Several times when I cough	Some times when I cough	Occasionally when I cough	Rarely	Never

Thank you for completing this questionnaire.

SAMPLE

Reference source: Development of a symptom specific health status measure for patients with chronic cough: Leicester Cough Questionnaire (LCQ). http://dx.doi.org/10.1136/thorax.58.4.339.

14.4 Appendix 4 Cough Visual Analogue Scale

Please put a cross on the line to indicate the severity of your cough in the past 2 weeks.

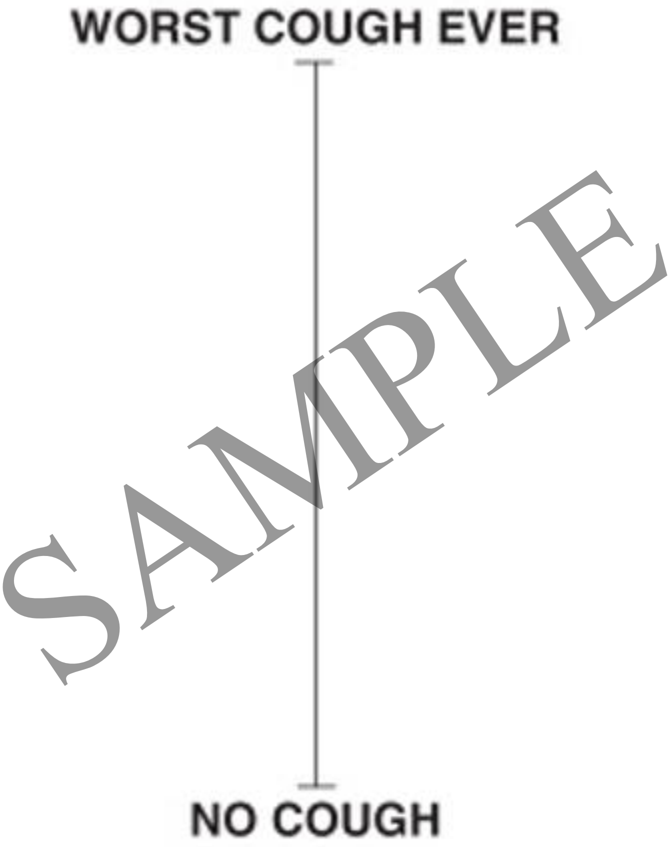

Note this scale is 100 mm long.

SAMPLE

14.5 Appendix 5 Protocol Amendments

Protocol Amendment and Date	Section and Summary of Changes	Reason
Version 5 27Apr2023	• Section 4.1.1 – Changed diagnosis to within last 3 years, removed FVC upper limit, updated DLCO upper limit to 80, and allowed historical treatment of previous anti-fibrotic therapy to within 2 months of the Baseline bronchoscopy<br>• Section 7.2 – Update PK sample to 5 minutes post-dose instead of 30 minutes	• Allows for a broader subject population<br>• More likely to measure LTI-03 level above lower limit of detection
Version 4 04Jan2023	• Administrative Changes<br>• Section 6.1.4 – Added discontinuation for decrease from Baseline in FEV1 of 15%<br>• Section 6.7 – Added additional post-dose and as needed spirometry<br>• Section 8.8 – Added unrelated similar SAE or severe or higher AE in > 2 subjects' criterion to stopping rules	• Administrative changes<br>• Added safety measure<br>• Additional monitoring for potential airway obstruction<br>• Additional safety measure
Version 3 06Oct2022	• Section 1.1.1, 1.2, and 1.3 – Update language to include rationale for placebo and SOC medication<br>• Section 2.2.2 – Added Leicester Cough Questionnaire and Cough Visual Analogue Scale to safety endpoints	• Provides rationale for SOC medication restriction during dosing of LTI-03<br>• Data to be summarized as endpoints

	• Section 3 – Describe safety reviews<br>• Section 4.12 – Update exclusion criteria to match investigator's brochure<br>• Section 5.6 – Add language to allow investigators to use SOC medications<br>• Section 6.0 and 6.9 – Update to provide both BAL and BA can be completed.<br>• Section 8.8 – Update to clarify stopping rules and describe protocol amendment requirements<br>• Section 9.3 – Remove analysis populations<br>• Section 9.4.7 Added data analysis description after Cohort 1<br>• Administrative changes	• Update to provide details regarding the safety review meetings to occur prior to dose escalation<br>• Updated to match investigator's brochure special populations<br>• Provides flexibility for investigators to allow SOC medications if necessary<br>• Allows for flexibility for investigational site to use SOC procedures<br>• Provide clear details when stopping rules would apply<br>• Details to be provided in the SAP<br>• Allows for analysis of Cohort 1<br>• Administrative changes
Version 2 17Jun2022	• Section 3.0 and 6.9 - Addition of bronchoabsorption<br>• Section 4.1 - Addition of serum to pregnancy test<br>• Section 7.1 - Allowing rescreening of subjects<br>• Section 7 – Administrative changes	• Allows for flexibility for investigational site to use SOC procedures<br>• Allows for flexibility for investigational site to use SOC procedures based on local regulations<br>• Subjects can be re-screened once<br>• Administrative changes

Supplementary Note 2. Statistical Analysis Plan for Study LTI-03-1002

Lung Therapeutics, Inc.

LTI-03-1002

A Randomized, Double-Blind, Placebo-Controlled, Phase 1b, Dose Escalation, Safety, Tolerability and Pharmacodynamic Biomarker Study of Caveolin-1-Scaffolding-Protein-Derived Peptide (LTI-03) in Recently Diagnosed, Treatment Naïve Subjects with Idiopathic Pulmonary Fibrosis

10OCT2024

Statistical Analysis Plan

Version *2.0*

Prepared by:

PPD
929 North Front Street Wilmington
NC 28401-3331

**Client:**	**Lung Therapeutics Inc.**
**Protocol Number:**	***LTI-03-1002***

**Document Description:**	Final Statistical Analysis Plan
**SAP Title:**	A Randomized, Double-Blind, Placebo-Controlled, Phase 1b, Dose Escalation, Safety, Tolerability and Pharmacodynamic Biomarker Study of Caveolin-1-Scaffolding-Protein-Derived Peptide (LTI-03) in Recently Diagnosed, Treatment Naïve Subjects with Idiopathic Pulmonary Fibrosis
**SAP Version Number:**	2.0
**Effective Date:**	10OCT2024

Author(s):

For PPD: Ichraq Latar, Senior Biostatistician I

For Lung Therapeutics: Mikki Behnke, Statistical Consultant

Approved by:

Signed by:
Ichraq Latar
Signer Name: Ichraq Latar
Signing Reason: I approve this document
Signing Time: 21-Oct-2024 | 1:23:55 PM GMT
7ED091F404EE412284998939BB80C4B7

Ichraq Latar Part of Thermo Fisher Scientific — Date *(DD-MMM-YYYY)*

Signed by:
Mikki Behnke
Signer Name: Mikki Behnke
Signing Reason: I approve this document
Signing Time: 17-Oct-2024 | 5:56:04 PM GMT
7B3A3A801DEE4B9688DF9FC97DD6D19C

Mikki Behnke, Statistical Consultant, Lung Therapeutics Inc. — Date *(DD-MMM-YYYY)*

DOCUMENT HISTORY

Version	Date	Changes
Version 1.0	01Sep2023	Initial version
Version 2.0	10Oct2024	Protocol deviation severity and significant status defined, 15% baseline FEV1 replaced by 15% decrease from Baseline FEV1, rule for BLQ value in biomarkers data

TABLE OF CONTENTS

LIST OF ABBREVIATIONS ... VI

1. INTRODUCTION ... 1

2. OBJECTIVES ... 1

2.1. PRIMARY OBJECTIVE ... 1
2.2. EXPLORATORY OBJECTIVES ... 1

3. INVESTIGATIONAL PLAN ... 1

3.1. OVERALL STUDY DESIGN AND PLAN ... 1
3.2. STUDY ENDPOINTS ... 2
3.2.1. Primary Endpoint ... 2
3.2.2 Other Safety Endpoints ... 2
3.2.3. Exploratory Endpoints ... 2
3.3. TREATMENTS ... 3
3.4. DOSE ADJUSTMENT/MODIFICATIONS ... 3

4. GENERAL STATISTICAL CONSIDERATIONS ... 3

4.1. BASELINE, END OF STUDY AND STUDY DAY ... 4
4.2. MISSING/INCOMPLETE START AND STOP DATES ... 4
4.3. SAMPLE SIZE ... 5
4.4. RANDOMIZATION AND BLINDING ... 5
4.5. ANALYSIS SETS ... 5
4.5.1. Enrolled Set ... 5
4.5.2. Randomized Set ... 5
4.5.3. Safety Set ... 6
4.5.4. Pharmacokinetics (PK) Set ... 6
4.5.5. Pharmacodynamics (PD) Set ... 6

5. SUBJECT DISPOSITION AND PROTOCOL DEVIATIONS ... 6

5.1. SUBJECT DISPOSITION ... 6
5.2. PROTOCOL DEVIATIONS ... 6

6. DEMOGRAPHICS AND BASELINE CHARACTERISTICS ... 7

6.1. DEMOGRAPHICS AND BASELINE CHARACTERISTICS ... 7
6.2. MEDICAL HISTORY ... 7
6.2.1. General Medical History ... 7
6.2.2. Disease-Specific History ... 7
6.3. INCLUSION AND EXCLUSION CRITERIA ... 7

7. TREATMENTS AND MEDICATIONS ... 8

7.1. PRIOR AND CONCOMITANT MEDICATIONS ... 8
7.1.1. Prior Medications ... 8
7.1.2. Concomitant Medications ... 8
7.2. CONCOMITANT PROCEDURES ... 8
7.3. EXTENT OF EXPOSURE AND TREATMENT COMPLIANCE RATE ... 8

8. SAFETY ANALYSIS 8
8.1 Adverse Events 9
8.1.1. Incidence of Treatment-Emergent Adverse Events 9
8.1.2. Relationship of Adverse Events to Study Drug 9
8.1.3. Severity of Adverse Event 10
8.1.4. Serious Adverse Events 11
8.1.5. Adverse Events Leading to Treatment Discontinuation 11
8.1.6. Adverse Events Leading to Death 11
8.2. Clinical Laboratory Evaluations 11
8.2.4. Hematology 12
8.2.5. Serum Chemistry 12
8.2.6. Coagulation 13
8.2.7. Pregnancy Test 13
8.3. Vital Sign Measurements 13
8.4. Physical Examination 13
8.5. Electrocardiogram 14
8.6. Spirometry 14
8.7. Leicester Cough Questionnaire 15
8.8. Cough Visual Analogue Scale 15
9. PHARMACOKINETICS 15
10. EXPLORATORY BIOMARKER SAMPLING 15
11. INTERIM ANALYSIS 16
12. CHANGES IN THE PLANNED ANALYSIS 17
13. REFERENCES 18
14. APPENDICES 19
14.1. Schedule of Assessments 19
14.2. Leicester Cough Questionnaire 21
14.3. Cough Visual Analogue Scale 22

List of Abbreviations

AE	Adverse Event
AEC2	Alveolar Epithelial Type 2 Cells
ALP	Alkaline Phosphatase
ALT	Alanine Aminotransferase
aPTT	activated Partial Thromboplastin Time
AST	Aspartate Aminotransferase
ATS	American Thoracic Society
AV	Atrioventricular
BA	Bronchoabsorption
BAL	Bronchoalveolar Lavage
BALF	Bronchoalveolar Lavage Fluid
BID	Twice daily
BUN	Blood Urea Nitrogen
CK	Creatine Kinase
CRF	Case Report Form
CRO	Contract Research Organization
CRP	C-reactive protein
CSD	Caveolin-1 Scaffolding Domain
CTCAE	Common Terminology Criteria for Adverse Events
CTMS	Clinical Trial Management System
DLCO	Diffusion Capacity of the Lungs for Carbon Monoxide
ECG	Electrocardiogram
ECM	Extracellular Matrix
EOS	End of Study
ERS	European Respiratory Society
EU	European Union
FEV_1	Forced Expiratory Volume 1
FLF	Fibrotic Lung Fibroblast
FN	Fibronectin
FOCBP	Female of Childbearing Potential
FVC	Forced Vital Capacity

GGT	Gamma Glutamyltransferase
HDL-C	High Density Lipoprotein Cholesterol
Hgb	Hemoglobin
HR	Heart Rate
HRCT	High-resolution Computed Tomography
IB	Investigator’s Brochure
ICH	International Council for Harmonization
ILD	Interstitial Lung Disease
IPF	Idiopathic Pulmonary Fibrosis
IVRS	Interactive Voice Response System
LCQ	Leicester Cough Questionnaire
LDH	Lactate Dehydrogenase
LDL-C	Low Density Lipoprotein Cholesterol
MCH	Mean Corpuscular Hemoglobin
MCHC	Mean Corpuscular Hemoglobin Concentration
MCV	Mean Corpuscular Volume
MedDRA	Medical Dictionary for Regulatory Activities
NCI	National Cancer Institute
PD	Pharmacodynamic
PR	P to R interval
PK	Pharmacokinetic
PT	Prothrombin Time
QD	Once Daily
QRS	QRS wave
QT	Q to T interval
QTcF	QT interval corrected for heart rate calculated using Fridericia’s formula
RBC	Red blood cell
RDW	Red cell Distribution Width
RR	R to R interval for ECGs
SAE	Serious Adverse Event
SAP	Statistical Analysis Plan
SAS	Statistical Analysis System

SOA	Schedule of Assessments
TEAE	Treatment-Emergent Adverse Event
VAS	Visual Analogue Scale
WBC	White Blood Cell
WHODD	World Health Organization Drug Dictionary

1. Introduction

This Statistical Analysis Plan (SAP) defines the statistical methods and data presentations for the Lung Therapeutics, Inc. protocol LTI-03-1002 study entitled "A Randomized, Double-Blind, Placebo-Controlled, Phase 1b, Dose Escalation, Safety, Tolerability and Pharmacodynamic Biomarker Study of Caveolin-1-Scaffolding-Protein-Derived Peptide (LTI-03) in Recently Diagnosed, Treatment Naïve Subjects with Idiopathic Pulmonary Fibrosis".

This SAP covers all specified analysis for the final study reports based on the study protocol Version 5.0 dated 04 May2023.

2. Objectives

2.1. Primary Objective

- To determine the safety and tolerability of inhaled LTI-03 in subjects diagnosed with IPF within 3 years of Screening.

2.2. Exploratory Objectives

- To assess the plasma pharmacokinetic (PK) of inhaled LTI-03 after the first dose and at steady state in subjects with IPF diagnosed within 3 years of Screening.
- To evaluate the pharmacodynamic (PD) biomarkers of inhaled LTI-03 in subjects diagnosed with IPF within 3 years of Screening.

3. Investigational Plan

3.1. Overall Study Design and Plan

This is a randomized, double-blind, placebo-controlled, multi-center, dose escalation, safety and tolerability study of LTI-03 or placebo administered by inhalation in subjects with IPF without prior treatment with anti-fibrotic agents within 2 months of the Baseline bronchoscopy. An IPF diagnosis will be confirmed by high-resolution computed tomography (HRCT) of chest or lung biopsy within 3 years prior to Screening. The study will contain 2 dose cohorts which will run sequentially. Subjects in the first, low-dose cohort (Cohort 1) will receive 2.5 mg of study drug (LTI-03 or placebo), administered twice daily approximately 10-12 hours apart (5 mg per day). If the 5 mg per day dose is well tolerated, a second, high-dose cohort (Cohort 2) will receive 5 mg of study drug (LTI-03 or placebo), administered twice daily approximately 10-12 hours apart (10 mg per day).

Eligible subjects will be randomized in a 3:1 ratio to either LTI-03 or placebo. Each cohort will have 9 subjects randomized to LTI-03 and 3 subjects randomized to placebo. Safety data will be reviewed on an ongoing basis by the Sponsor's medical officer and the Contract Research Organization (CRO)'s medical monitor. A review of safety data including but not limited to adverse events, laboratory assessments, vital signs, and spirometry will be conducted after all 12 subjects from Cohort 1 have completed Day 14 assessments. Enrollment in the second cohort will not begin until the Cohort 1 safety data has been reviewed and a dose decision made. A lower dose cohort may be added (2.5 mg administered once daily) or additional subjects (up to 12 subjects) may be added to the low-dose cohort (2.5 mg administered twice daily) if doses of

5 mg twice daily (10 mg per day) are not tolerated. In total, a maximum of approximately 24 subjects will be randomized in the study.

The Treatment Period will be 14 days, with subjects self-administering the study drug using the provided commercially available dry powder inhaler. All subjects will be trained on the proper methods of inhaler use and will have access to site personnel trained to assist subjects remotely if questions arise. Retraining may occur on Day 1 and 7, if required. During the Treatment Period, there will be on-site visits on Day 1, Day 7, and Day 14 and study assessments will occur at the investigational site on these days. The first dose of study drug will be administered at the investigational site and subjects will be monitored for at least 1 hour after dosing.

Screening assessments may take place over no more than 21 days prior to the Treatment Period. Screening assessments will include but not be limited to a complete physical examination including vital signs, electrocardiogram (ECG), spirometry, safety laboratory parameters, medical history, and concomitant medications. A Baseline bronchoscopy will be performed during the Screening Period in subjects who meet the Screening criteria and will include bronchoalveolar lavage (BAL) and/or bronchoabsorption (BA), and deep bronchial brushings.

Treatment Period will include but not be limited to physical examinations with vital signs, ECG, spirometry, clinical laboratory assessments, adverse event (AE) assessment, Leicester cough questionnaire, cough visual analogue scale (VAS), and concomitant medications. Bronchoscopies with BAL and/or BA and deep bronchial brushings will also be performed on Day 14 approximately 2-3 hours after morning dosing.

Follow-up study assessments will be completed at Day 21 with all Day 14 assessments repeated except dosing, bronchoscopy, and blood collection for biomarkers and PK. Also, a complete physical examination will be replaced by a brief physical examination. The end of the study is defined as the date of the last visit of the last participant in the study.

3.2. Study Endpoints

3.2.1. Primary Endpoint

- Incidence of Treatment Emergent Adverse Events (TEAE).

3.2.2 Other Safety Endpoints

- Other safety endpoints include:
    - Physical examination findings
    - Vital signs
    - ECG
    - Clinical laboratory measurements
    - Spirometry
    - Leicester Cough Questionnaire (LCQ)
    - Cough VAS

3.2.3. Exploratory Endpoints

- To determine the plasma peak and trough concentration of LTI-03 after the first dose and at steady state.

- Change from baseline over time for biomarkers related to the pathophysiology of IPF.

3.3. Treatments

LTI-03 and matching placebo will be provided as inhalation powders for oral inhalation use. LTI-03, as an excipient-free, micronized dry powder, will be placed into white opaque hypromellose two-piece capsules. The placebo will be micronized lactose powder that is placed into white opaque hypromellose two-piece capsules and will be identical in appearance to LTI-03. Individual capsules containing 2.5 mg of study drug (LTI-03 or placebo) will not be labelled.

3.4. Dose Adjustment/Modifications

No dose adjustment is planned. A lower dose cohort may be added (2.5 mg administered once daily (QD)) or additional subjects may be added to the low-dose cohort (2.5 mg administered BID) if doses of 5 mg BID (10 mg per day) are not tolerated. If a lower dose cohort is added, the treatments will be appropriately described/updated and summarized in the TFLs.

4. General Statistical Considerations

This section presents general rules for the derivation and reporting of study data. If a subsequent section related to a specific derivation or analysis differs from this general guidance, the subsequent section takes precedence.

Continuous data will be presented using descriptive statistics (i.e. n, mean, standard deviation, median, minimum, and maximum). Categorical data will be presented using frequency counts and percentages in each category. For the summary statistics of all numerical variables (unless otherwise specified), minimum and maximum will be displayed to the same level of precision of the raw data. Mean and median will be displayed to one level of precision greater than the data collected. Standard deviation will be displayed to two levels of precision greater than the data collected.

When count data are presented, the percentage will be suppressed when the count is zero in order to draw attention to the non-zero counts. A row denoted “Missing” will be included in count tabulations where specified on the shells to account for missing values. The denominator for all percentages will be the number of subjects in that treatment group within the analysis set of interest, unless otherwise specified. Percentages will be presented to one decimal place except for the display of 100% frequency which will be displayed as ‘XX (100)’.

In general, all tables will be presented by treatment (active treatments, active treatment total, and pooled placebo). Subjects from all cohorts who took placebo will be combined to form a pooled placebo group for analyses. All study data will be presented in by-subject data listings.

“No data available for this report” will be presented when there are no data available to report.

The primary endpoint of the study is safety and no formal statistical hypotheses will be tested.

All descriptive statistical analyses will be performed using Statistical Analysis System (SAS) statistical software (version 9.4 or higher). Missing data will not be imputed unless otherwise stated.

4.1. Baseline, End of Study and Study Day

Unless otherwise specified, baseline will be defined as the last non-missing evaluation prior to the date and time that the first dose of treatment is taken. Baseline values for spirometry are the mean of the Day 1 pre-dose and the value collected during screening using the same spirometry equipment (see Section 8.6).

A participant will have completed the study if all visits are completed and the End of Study eCRF is marked as completed. The end of study date for each participant is defined as the date of the last visit as recorded on the End of Study eCRF.

Study day is defined in relation to the date of first dose of study drug (on Day 1). Therefore, when an assessment date is before the first dose:

Study day = assessment date - first dose date of study drug

and when an assessment is on or after the first dose:

Study day = assessment date - first dose date of study drug + 1

4.2. Missing/Incomplete Start and Stop Dates

For the purpose of inclusion in tables, incomplete start and stop dates and times for AEs and prior/concomitant medications will be imputed as follows:

Missing start dates (where UK, UNK and UNKN indicate unknown or missing day and month respectively) will be handled as follows:

- UK-MMM-YYYY:
  - If the month and year are different from the month and year of the first dose of study drug, assume 01-MMM-YYYY.
  - If the month and year are the same as the first dose of study drug month and
    - the end date (after any imputation) is on or after the first dose of study drug, then the start date is assumed to be the date of the first dose of study drug.
    - the end date (after any imputation) is prior to the first dose of study drug, then assume the end date for the start date.

- DD-UNK-YYYY/UK-UNK-YYYY:
  - If the year is different from the year of first dose of study drug, assume 01-JAN-YYYY of the collected year.
  - If the year is the same as the first dose of study drug year and
    - the end date (after any imputation) is on or after the first dose of study drug, then the start date is assumed to be the date of the first dose of study drug.
    - the end date (after any imputation) is prior to the first dose of study drug, then the start date is assumed to be the end date for the start date.

If the start date is not recorded on eCRF then no date imputation will be performed.

Missing start times will be imputed as follows: If the missing time of an actual or imputed start date is the same as a study drug dosing date, the dosing time will be used as the imputed time. Otherwise, the earliest possible time of the actual or imputed start date (i.e. 00:01) will be used as the imputed time.

Missing stop dates (where UK and UNK indicate unknown or missing day and month respectively) will be handled as follows:

- UK-MMM-YYYY: Stop date is assumed to be the last day of the month;
- DD-UNK-YYYY/UK-UNK-YYYY: Stop date is assumed to be 31-DEC-YYYY.

If a subject dies during the study, the stop date will be imputed as the date of death if the imputed stop date is after date of death.

Missing stop times will be imputed as the latest possible time of the actual or imputed stop date (i.e. 23:59).

4.3. Sample Size

Approximately 24 subjects are planned in 2 cohorts. Each cohort will include 12 subjects (9 on LTI-03 and 3 on placebo). Selection of sample size is based on prior experience to ensure that the safety and tolerability of LTI-03 will be adequately assessed while minimizing unnecessary subject exposure. With 18 subjects receiving LTI-03 there is a 60% chance of detecting an adverse event (AE) with a true incidence rate of 5% and an 85% chance of detecting a more common AE with a true incidence rate of 10%.

4.4. Randomization and Blinding

Subjects will be randomized to receive LTI-03: placebo in sequential cohorts, as per the study design. Randomization codes and unique subject identification numbers will be generated by the Interactive Voice Response System (IVRS). All study staff will remain blinded to study drug assignment throughout the trial.

An unblinded team of Biostatistics personnel will be set up to handle unblinded data until the study is unblinded after data lock.

4.5. Analysis Sets

The following analyses sets will be used for the various analyses outlined in this SAP.

4.5.1. Enrolled Set

The Enrolled Set will consist of all subjects who signed the informed consent form (ICF), to include screen failures.

A screen failure is defined as a subject who consents to participate in the study but is not subsequently randomized.

4.5.2. Randomized Set

The Randomized Set will consist of all subjects who were enrolled and randomized to study drug.

4.5.3. Safety Set

The Safety Set will consist of all subjects randomized to study drug and received at least one dose of study drug. The Safety Set will be used for all safety analyses and subjects will be analyzed according to the study drug actually received.

4.5.4. Pharmacokinetics (PK) Set

The PK Set will consist of all subjects who receive at least one dose of LTI-03 and have at least one evaluable PK concentration.

4.5.5. Pharmacodynamics (PD) Set

The PD Set will consist of all subjects who received at least one dose of LTI-03 and have at least one measured value at a scheduled PD time point after the start of dosing for at least one PD analyte.

5. Subject Disposition and Protocol Deviations

5.1. Subject Disposition

Subjects will be summarized by the number and percent of subjects dosed, completed the study, discontinued from the study (with discontinuation reasons), completed treatment, and discontinued from treatment (with treatment discontinuation reasons). The number of subjects randomized (i.e., subjects with a randomization date/time/number recorded on the eCRF) will also be summarized. Subject disposition summary will be presented by treatment (active treatments, active treatment total, and pooled placebo) and overall.

All subject disposition data recorded on the eCRF, including for subjects not randomized will be listed by subject using the Enrolled Set.

5.2. Protocol Deviations

All protocol deviations will be recorded and documented according to the Study Deviation Rules document in PPD's Clinical Trial Management System (CTMS) system. Once the deviations have been finalized, a MS Excel file with all deviations will be converted into a SAS dataset and used as the source for the SDTM DV domain to generate a listing. Protocol deviations will be tabulated by severity (i.e., Major/Minor). The summary table will include the number of subjects reporting protocol deviations and as a percent of the number of subjects dosed by treatment (active treatments, active treatment total, and pooled placebo) and overall. If a subject reports protocol deviations at more than one level of severity, both categories will be summarized during analysis.

The following definitions will be used:

- Major protocol deviations are defined as those deviations with substantial effect on patient safety or data integrity.
- Minor protocol deviations are defined as those deviations with non-substantial effect on patient safety or data integrity.
- Significant protocol deviations are defined as those deviations which affect the analysis population.

- Non-significant protocol deviations are defined as those deviations which do not affect the analysis population.

The summary and listing of the protocol deviations will be provided for the Safety Set.

6. Demographics and Baseline Characteristics

6.1. Demographics and Baseline Characteristics

The number and percentage of subjects by sex (Male, Female), reproductive status (Of Child-bearing Potential, Surgically Sterile, Post-menopausal), race (White, Black or African American, Asian, American Indian or Alaska Native, Native Hawaiian or Other Pacific Islander, Not Reported, Unknown and Other) and ethnicity (Hispanic or Latino, Not Hispanic or Latino, Not Reported and Unknown) will be summarized.

The baseline characteristics consist of age (years), baseline height (cm), baseline weight (kg), baseline body mass index (BMI) (kg/m^2), number of cigarettes (or the equivalent) per day (pack-years (applicable only to subjects with past and/or current tobacco use)), and Diffusion Capacity of the Lungs for Carbon Monoxide (DLCO) (measured value only, in units of mmol/min/kPa), baseline spirometry (FVC), baseline LCQ total score and baseline cough VAS score. Demographics and baseline characteristics will be summarized descriptively.

Body mass index is calculated as (body weight in kilograms) / $(\text{height in meters})^2$. A subject's age in years is calculated using the date of the informed consent and date of birth.

Cigarette consumption will be calculated in pack-years using: (the number of cigarettes smoked per day/20) * number of years smoked.

The summary of subject demographics and baseline characteristics will be presented by treatment (active treatments, active treatment total, and pooled placebo) and overall. Subject demographics and baseline characteristics will be listed. The summary and listing of demographics and baseline characteristics will be provided for the Safety Set.

6.2. Medical History

6.2.1. General Medical History

Medical history will be coded using the Medical Dictionary for Regulatory Activities (MedDRA) version 26.0 or higher by system organ class (SOC) and preferred term (PT).

Subject medical history data including specific details as collected in the eCRF will be presented in a listing for the Safety Set.

6.2.2. Disease-Specific History

Details of IPF diagnosis (diagnostic method and date of test) will be listed for the Safety Set.

6.3. Inclusion and Exclusion Criteria

The details of the inclusion and exclusion criteria can be found in Sections 4.1.1 and 4.1.2 of the study protocol. The status of eligibility criteria met, inclusion criteria not met, or exclusion criteria met details will be listed for the Enrolled Set.

7. Treatments and Medications

7.1. Prior and Concomitant Medications

All medications (including over-the-counter medicines, herbal treatments, supplements, and vitamins) administered within 30 days of the Screening Visit through Study Day 21 will be recorded on the Case Report Form (CRF), using generic names when possible.

Prior and concomitant medication will be coded according to the latest World Health Organization Drug Dictionary (WHODD) version March, 2023 or later. Missing/partial start and stop dates will be imputed as specified in Section 4.2.

The listing of prior and concomitant medications will be provided for the Safety Set. All prior and concomitant medications will be presented in the same listing.

7.1.1. Prior Medications

A prior medication is defined as a medication that ends prior to the first study drug administration.

7.1.2. Concomitant Medications

A concomitant medication is defined as a medication that ends at the time of or after the first study drug administration.

7.2. Concomitant Procedures

All concomitant procedures recorded during the study will be listed. The listing will be provided for the Safety Set.

7.3. Extent of Exposure and Treatment Compliance Rate

Exposure to study drug is defined as administration of any amount of study drug. Duration of exposure is defined as the total number of days a subject is exposed to study drug and will be presented as the total number of days from the first dose date and time (Day 1) to the last dose date and time (date of last dose minus the date of first dose + 1) as recorded in the eCRF.

Drug compliance over the total dosing period will be assessed as the rate of compliance. The rate of compliance (%) will be calculated as follows and recorded on the eCRF: [(number of capsules used/number of capsules expected to be used)]*100.

The extent of exposure and rate of compliance will be summarized by treatment (active treatment arms, total of active arms, pooled placebo) and overall. A listing of exposure and compliance rate data will be presented.

The summary table and listing of exposure and compliance rate will be provided for the Safety Set.

8. Safety Analysis

Safety will be evaluated through continuous monitoring of AEs, physical examinations, vital signs, clinical laboratory measurements, spirometry, and ECGs.

Safety summaries and listings will be presented for the Safety Set.

8.1 Adverse Events

According to the International Council for Harmonization (ICH) guideline for GCP, an AE is any untoward medical occurrence in a subject or clinical investigation subject administered a pharmaceutical product, and which does not necessarily have to have a causal relationship with the treatment. An AE can therefore be any of the following:

- Any unfavorable and unintended sign (including an abnormal laboratory finding), symptom, or disease temporally associated with the use of a medicinal product, whether or not considered related to the medicinal product.
- Any new disease or exacerbation of an existing disease (a worsening in the character, frequency, or severity of a known condition).
- Recurrence of an intermittent medical condition (e.g., headache) not present at baseline.
- Any deterioration in a laboratory value or other clinical test (e.g., ECG) that is associated with symptoms or leads to a change in study treatment or concomitant treatment or discontinuation from study drug.
- Adverse events that are related to a protocol-mandated intervention, including those that occur prior to assignment of study treatment (e.g., Screening invasive procedures such as biopsies).

Adverse events will be coded using the current version of Medical Dictionary for Regulatory Activities (MedDRA® Version 26.0 or higher). Missing/partial AE dates and times will be imputed as specified in Section 4.2. In general, summaries will be provided for the Safety Set and presented by SOC, PT and treatment (active treatments, active treatment total, and pooled placebo) and overall. Participants will be counted only once within each SOC or PT.

All AEs will be presented in a listing.

8.1.1. Incidence of Treatment-Emergent Adverse Events

AEs which begin or increase in severity or frequency at or after the administration of first dose of study drug are to be considered as TEAEs.

An overall summary of TEAEs will include the number and percentage of participants with at least one TEAE, any TEAE with CTCAE severity Grade 3 or higher, any related TEAE, any serious TEAE, any fatal TEAE, and any TEAEs leading to discontinuation of study drug.

The number and percentage of participants with TEAEs and the total number of TEAEs will be summarized by SOC and PT for each treatment (active treatments, active treatment total, and pooled placebo) and overall.

8.1.2. Relationship of Adverse Events to Study Drug

The Investigator's assessment of causality must be provided for all AEs (serious and non-serious). An Investigator's causality assessment is the determination of whether there exists a reasonable possibility that the investigational product caused or contributed to an AE.

- Not related: The AE is clearly not related to the study drug, or the temporal relationship of the onset of the AE relative to administration of the product is not reasonable, or the AE can

be explained by another cause such as an underlying medical condition or other concomitant medication, or the AE has no plausible relationship to study drug.

- Related: The AE is probably related to the study drug. The temporal relationship of the AE to administration of the product is reasonable and there is no other cause to explain the event. Adverse events should be classified as related if the Investigator feels there is evidence to suggest a causal relationship between the study drug and the AE.

Study drug related TEAEs will be summarized by SOC and PT for each treatment (active treatments, active treatment total, and pooled placebo) and overall.

8.1.3. Severity of Adverse Event

All AEs will be assessed according to the National Cancer Institute (NCI) Common Terminology Criteria for Adverse Events (CTCAE), Version 5.

Table 1 will be used for assessing severity for AEs that are not specifically listed in the NCI CTCAE.

Table 1: Adverse Event Severity Grading Scale for Events not Specifically Listed in NCI CTCAE

**Grade**	**Severity**
1	Mild; asymptomatic or mild symptoms; clinical or diagnostic observations only; or intervention not indicated
2	Moderate; minimal, local, or non-invasive intervention indicated; or limiting age-appropriate instrumental activities of daily living [a]
3	Severe or medically significant, but not immediately life-threatening; hospitalization or prolongation of hospitalization indicated; disabling; or limiting self-care activities of daily living [b, c]
4	Life-threatening consequences or urgent intervention indicated [d]
5	Death related to adverse event [d]

a. Instrumental activities of daily living refer to preparing meals, shopping for groceries or clothes, using the telephone, managing money, etc.
b. Examples of self-care activities of daily living include bathing, dressing and undressing, feeding oneself, using the toilet, and taking medications, as performed by patients who are not bedridden.
c. If an event is assessed as a "significant medical event," it must be reported as a serious adverse event, per the definition of serious adverse event in Section 8.1.4.
d. Grade 4 and 5 events must be reported as serious adverse events, per the definition of serious adverse event in Section 8.1.4.

Reference source: Common Terminology Criteria for Adverse Events (CTCAE) version 5.0 published on November 27, 2017.

CTCAE severity grade will be presented in the AE listing. TEAEs by maximum severity will be summarized by SOC and PT for each treatment (active treatments, active treatment total, and pooled placebo) and overall.

8.1.4. Serious Adverse Events

Serious Adverse Events include adverse events that result in death, require either inpatient hospitalization or the prolongation of hospitalization, are life-threatening, result in a persistent or significant disability/incapacity, result in a congenital anomaly/birth defect, or are a significant medical event in the Investigator's judgement.

All serious TEAEs will be presented in a separate listing.

8.1.5. Adverse Events Leading to Treatment Discontinuation

The AEs where the answer to "Action Taken" on the AE eCRF is "Drug Withdrawn" will be identified in the AE listing.

8.1.6. Adverse Events Leading to Death

The AEs where the answer to "Outcome" on the AE eCRF is "Death" will be identified in the AE listing.

8.2. Clinical Laboratory Evaluations

Clinical laboratory assessments will be performed by a central laboratory and collected according to the SOA (see Appendix 14.1).

For all numeric serum chemistry and hematology laboratory values, descriptive statistics for observed and change from baseline values will be presented for each laboratory test by scheduled visit and treatment (active treatments, active treatment total, and pooled placebo) and overall for the Safety Set. Listings will include all measured values.

All relevant clinical laboratory tests will be classified as Low, Normal, and High according to the normal ranges. This categorical data will be summarized in shift tables comparing the minimum post-baseline value, maximum post- baseline value, and the results at timepoint of interest post-baseline visit with those at the baseline visit. Shift table summaries will be

presented by treatment (active treatments, active treatment total, and pooled placebo) and overall for the Safety Set.

8.2.4. Hematology

The following hematology laboratory tests will be included:

- red blood cell (RBC) count
- mean corpuscular hemoglobin concentration (MCHC)
- mean corpuscular hemoglobin (MCH)
- mean corpuscular volume (MCV)
- red cell distribution width (RDW)
- hematocrit (Hct)
- hemoglobin (Hgb)
- white blood cell (WBC) count
- including differential count (neutrophils, eosinophils, basophils, lymphocytes, monocytes)
- platelet count

Summary tables will be presented as described in Section 8.2 and data will be provided in a listing.

8.2.5. Serum Chemistry

The following serum chemistry laboratory tests will be included:

- bilirubin (total and conjugated)
- aspartate aminotransferase (AST)
- alanine aminotransferase (ALT)
- alkaline phosphatase (ALP)
- gamma glutamyl transferase (GGT)
- lactate dehydrogenase (LDH)
- creatine kinase (CK)
- protein
- albumin
- cholesterol (total, low density lipoprotein cholesterol (LDL-C))
- high density lipoprotein cholesterol (HDL-C)
- glucose
- blood urea nitrogen (BUN)
- creatinine
- sodium
- potassium
- chloride
- calcium
- magnesium
- C-reactive protein (CRP)

Summary tables will be presented as described in Section 8.2 and data will be provided in a listing.

8.2.6. Coagulation

The following coagulation laboratory tests will be included:

- prothrombin time (PT)
- INR
- activated partial thromboplastin time (aPTT)

Since coagulation data are collected only at the screening visit, only a listing will be presented.

8.2.7. Pregnancy Test

Females of childbearing potential must have a negative urine or serum pregnancy test during screening. A urine or serum pregnancy test will also be performed on study days as mentioned in the schedule of assessments (SOA) (see Appendix 14.1).

A listing showing pregnancy test date and time, test result and reason for not doing pregnancy test (if not done) will be presented for the Safety Set.

8.3. Vital Sign Measurements

Vital signs will be obtained after subject has been sitting for at least 1 minute and will include systolic blood pressure (mmHg), diastolic blood pressure (mmHg), heart rate (beats/minute), respiratory rate (breaths/minute), body temperature (C) and hemoglobin oxygen saturation (%) (via pulse oximetry).

Descriptive statistics for observed and change from baseline values will be presented for each vital sign test by scheduled visit and treatment (active treatments, active treatment total, and pooled placebo) and overall for the Safety Set.

Vital signs data will be presented in a listing for the Safety Set.

8.4. Physical Examination

A complete physical examination will include an evaluation of the head, eyes, ears, nose and throat, and the cardiovascular, dermatologic, musculoskeletal, respiratory, gastrointestinal, and neurologic systems. Any abnormality identified at baseline (Screening Physical Exam) will be recorded as medical history.

A brief physical examination will focus on systems affected by AEs and at a minimum should include the cardiovascular and respiratory systems. Height and weight will be recorded at screening.

Changes from baseline abnormalities should be recorded in subject notes. New or worsened clinically significant abnormalities should be recorded as TEAEs. A categorical summary of physical examination results will be presented by scheduled visit and treatment (active treatments, active treatment total, and pooled placebo) and overall for the Safety Set.

Physical examination data will be presented in a listing for the Safety Set.

8.5. Electrocardiogram

A single standard 12-lead ECG will be performed at each scheduled visit. Lead placement should be as consistent as possible. ECG recordings must be performed after the subject has been resting in a supine position for at least 5 minutes.

ECG parameters to be evaluated include heart rate (HR) and the R to R interval (RR), Q to T (QT), P to R (PR) intervals and QRS duration. In addition, Fridericia's formula should be used to calculate the QT interval corrected for heart rate (QTcF). ECG morphology statements will also be evaluated with particular attention paid to waveform morphology findings of: ST segment abnormal, T wave changes, abnormal U waves, atrioventricular (AV) block, arrhythmia (supraventricular, ventricular, atrial fibrillation/flutter, etc.), and others including infarction, ischemia, and hypertrophy. ECG abnormalities will be recorded as AEs only if they are considered to be clinically significant by the Investigator.

Descriptive statistics for observed and change from baseline values will be presented for each ECG measurement by scheduled visit and treatment (active treatments, active treatment total, and pooled placebo) and overall for the Safety Set.

All ECG data will be listed with flags for QTcF values > 450 msec and increase from baseline > 30 msec.

8.6. Spirometry

Spirometry to assess forced expiratory volume 1 (FEV1), FVC, FEV1/FVC and the percent predicted for each of the parameters will be performed during Screening, Day 1, Day 7, Day 14, and Day 21. DLCO will be assessed only at Screening. Percent predicted will be calculated based on height, age, and ethnicity. Spirometry will be performed according to the current American Thoracic Society (ATS) / European Respiratory Society (ERS) Task Force guidelines (Miller et al., 2005). Spirometry will be performed on Days 1, 7, and 14 pre-dose and post-dose at 30 and 60 minutes (See Protocol section 7). Unscheduled spirometry may as be performed as needed for respiratory symptoms. If bronchospasm is suspected, spirometry may also be performed before and after a rescue beta-adrenergic agonist inhalation.

It is important to note that baseline values for spirometry are the mean of the Day 1 pre-dose and the value collected during screening using the same spirometry equipment. For example, if the initial spirometry for screening is done in a central spirometry laboratory, but Day 1 and subsequent spirometry is done with portable equipment in the clinic, then a second spirometry should be done in the screening period using the same portable equipment used in the clinic. The baseline value for each spirometry would thus be the mean of screening value using portable equipment and the Day 1 pre-dose value using portable equipment.

Descriptive statistics for observed and change from baseline values will be presented for each spirometry measurement by scheduled visit, timepoint, and treatment (active treatments, active treatment total, and pooled placebo) and overall for the Safety Set.

Line graphs of FEV1, FVC and 15% decrease from Baseline FEV1 will be presented by subject and visit for the Safety Set.

Spirometry data will be presented in a listing for the Safety Set.

8.7. Leicester Cough Questionnaire

The Leicester Cough Questionnaire (LCQ) is a subject self-report, health related quality of life questionnaire (see Appendix 14.2) that will be collected per SOA (see Appendix 14.1). The LCQ consists of three domains where domain scores are equal to the total score from the questions in the domain divided by the number of questions in the domain (domains scores range from 1 to 7):

1. Physical: Questions 1, 2, 3, 9, 10, 11, 14, and 15
2. Psychological: Questions 4, 5, 6, 12, 13, 16, and 17
3. Social: Questions 7, 8, 18, and 19

LCQ total score is equal to the sum of the domain scores (total score range from 3 to 21) with a lower score indicating greater impairment of health status due to cough. The LCQ domain and total scores will be derived for use during analysis. If a response is not provided to a question, the domain score will be calculated as the average of the scores to the answered questions within the domain. If a subject responds to less than 80% of questions in a domain, the LCQ domain score will not be calculated and will be treated as missing during analysis. If it is not possible to calculate all domain scores following this criteria, total score will not be calculated and will be treated as missing during analysis.

Descriptive statistics for observed and change from baseline values will be presented for LCQ domain and total scores by scheduled visit and treatment (active treatments, active treatment total, and pooled placebo) and overall for the Safety Set.

LCQ data will be presented in a listing for the Safety Set.

8.8. Cough Visual Analogue Scale

The cough VAS is a 100 mm scale (See Appendix 14.3) on which a subject will be asked to mark between ‘no cough’ (0 mm) and ‘the worst cough severity’ (100 mm).

Descriptive statistics for observed and change from baseline values will be presented for cough severity VAS by scheduled visit and treatment (active treatments, active treatment total, and pooled placebo) and overall for the Safety Set.

Cough VAS data will be presented in a listing for the Safety Set.

9. Pharmacokinetics

Plasma and BAL samples for PK assessment will be collected as indicated in the SOA (see Appendix 14.1). Plasma PK analyses and BAL bioanalytical results will be covered by a vendor reported by the sponsor in a separate document.

10. Exploratory Biomarker Sampling

Exploratory biomarkers will be assessed in peripheral blood cells (as feasible), platelet rich plasma, BALF, BA samples, and deep bronchial brushings as outlined in Table 2. Ribonucleic acid (RNA) transcription analysis from whole blood may be substituted for any assays using peripheral blood cells. Samples will be collected at the time of bronchoscopy during the screening period and Day 14 as shown in Appendix 14.1. Additional biomarkers may be added as new information is generated about the potential effects of CSD (caveolin-1 scaffolding domain) signaling.

Table 2: Exploratory Biomarkers by Sample Source and Assumed Indicator Function

Sample source / Indicator of	Epithelial damage / repair	Fibrosis	Inflammation	Thrombosis
**Peripheral blood cells** [b]	-	p-AKT[b], IL-11	CXCL7	-
**Platelet rich plasma (PRP)**	CYFRA 21-1, SP-D, CA-19-9, KL-6, sRAGE, Galectin 7	MMP-7, Tenascin C (TNC), Periostin, IL-11, MYDGF, MMP-2	CCL18, CXCL13, slCAM1, IL-11, sCD163, CXCL7	PAI-1
**BAL and/or BA**[a]	Galectin 7, SP-D, sRAGE	MYDGF, MMP-2, TNC, MMP-7, periostin, IL-11	CCL18, CXCL13, slCAM1, IL-11, sCD163, CXCL7	PAI-1
**Deep bronchial brushings**	-	p-SMAD2/3	-	-

Abbreviations: BAL = bronchoalveolar lavage. BA = bronchoabsorption

[a] LTI-03 levels will also be measured on BAL samples. LTI-03 will not be measured in BA samples.

[b] Ribonucleic acid transcription analysis may be substituted for assays using peripheral blood cells.

For each PD biomarker, concentrations, change from baseline, percent change from baseline, and/or fold change, as applicable, will be summarized by scheduled visit and treatment (active treatments, active treatment total, and pooled placebo) and overall for all subjects in the Safety Set.

Biomarker results that are Below the Limit of Quantification will be treated as zero for calculation of concentration descriptive statistics. Percent change and fold change will be estimated for subjects with both baseline and post-baseline results, but will not be estimated in cases where the baseline result is zero.

Biomarker data will be presented in a listing for the Safety Set.

11. Interim Analysis

No formal interim analyses are planned for the study. Cohort 1 biomarker data may be analyzed before final database lock without changing the overall study conduct. If required, these analyses will be handled by Lung Therapeutics. PPD Biostatistics will provide relevant blinded SDTM and ADaM datasets to Lung Therapeutics to conduct the analyses. If requested, unblinded PPD Biostatistics personnel will be assigned and appropriate documentation updated to detail the unblinding event.

12. Changes in the Planned Analysis

Section 8.6: Line graphs of 15% Baseline FEV1 is updated to 15% decrease from Baseline FEV1.

13. References

Guenther A, Krauss E, Tello S, et al. The European IPF registry (eurIPFreg): baseline characteristics and survival of patients with idiopathic pulmonary fibrosis. Respir Res 2018;19(1):141. DOI: 10.1186/s12931-018-0845-5.

Lancaster, Lisa, et al. "Idiopathic pulmonary fibrosis: Physician and patient perspectives on the pathway to care from symptom recognition to diagnosis and disease burden." Respirology 27.1 (2022): 66-75.

Lederer DJ, Martinez FJ. Idiopathic pulmonary fibrosis. N Engl J Med. 2018;378:1811–23. doi:10.1056/NEJMra1705751.

Maher, Toby M., and Mary E. Strek. "Antifibrotic therapy for idiopathic pulmonary fibrosis: time to treat." Respiratory research 20.1 (2019): 1-9.

Martinez FJ, Collard HR, Pardo A, Raghu G, et al. Idiopathic pulmonary fibrosis. Nat Rev Dis Prim. 2017;3:17074. doi:10.1038/nrdp.2017.74.

Miller MR, Hankinson J, Brusasco V, et al. Standardisation of spirometry. Eur Respir J. 2005;26:319-38.

Snyder LD, Mosher C, Holtze CH, et al. Time to diagnosis of idiopathic pulmonary fibrosis in the IPF-PRO Registry. BMJ Open Respir Res 2020;7(1). DOI: 10.1136/bmjresp-2020-000567.

14. Appendices

14.1. Schedule of Assessments

	**Screening Period**	**Treatment Period**[1]			**Follow-Up**	**Early Termination**[2]
	**(Day -21 to Day -1)**	**Day 1**	**Day 7** (± 1 day)	**Day 14** (± 1 day)	**Day 21** (± 2 days)	
Informed consent	x					
Inclusion/Exclusion Criteria	x	x				
Demographics, Medical History	x					
Prior medications	x					
Concomitant medications	→	→	→	→	→	→
Complete physical examination[3]	x			x		x
Brief physical examination[4]		x	x		x	
Vital Signs	x	x	x	x	x	x
ECG (12-Lead ECG), single	x	x	x	x	x	x
Bronchoscopy with BAL and/or BA and deep bronchial brushings biopsy	x			x		x
Blood for biomarker assessments[5]	x			x		x
Pregnancy test[6]	x	x	x	x	x	x
Safety Laboratory parameters[7]	x	x	x	x	x	x
Randomization		x				
Blood collection for PK[8]		x	x	x		
Assessment of ability to use inhaler	x					
Leicester Cough Questionnaire & Cough VAS	x	x	x	x	x	x
Spirometry[9]	x	x	x	x	x	x

Dosing[10]		x	x	x		
Subjects returning unused study drug			x	x		
Adverse Events	x	→	→	→	→	→

Abbreviations: BAL = bronchoalveolar lavage; BA = bronchoabsorption; DLCO = diffusion capacity of the lungs for carbon monoxide; ECG = electrocardiogram; PK = pharmacokinetics; VAS = visual analogue scale

Note:

1. On Days 2 to 6 and Days 8 to 13, subjects will self-administer study drug at home and will be contacted on Day 4 (± 2 days) and Day 10 (± 2 days) by study staff to check on compliance and for potential adverse events. Day 1, 7, and 14 will be clinic visit. Study be clinic visit. Study staff will monitor subjects self-administrating study drug (most likely the morning dose) on Day 1, Day 7, and Day 14.
2. If a subject discontinues study drug before Day 14, the Day 14 assessments (other than study drug dosing) where feasible should be performed in the clinic as close as possible to the day of discontinuation. The EOS assessments should be performed 7 days after the last dose of study drug (assuming the subject has not withdrawn consent).
3. A complete physical examination will include an evaluation of the head, eyes, ears, nose and throat, and the cardiovascular, dermatologic, musculoskeletal, respiratory, gastrointestinal, and neurologic systems. Any abnormality identified at baseline (Screening Day) will be recorded as medical history.
4. A brief physical examination will focus on systems affected by AEs and at a minimum should include the cardiovascular and respiratory systems.
5. Biomarker assessment will be for peripheral blood cells, platelet rich plasma, bronchoalveolar lavage fluid (BALF), BA samples, and deep bronchial brushings.
6. Serum or urine test required for any FOCBP, defined as pre-menopausal or < 12 months of amenorrhea post-menopause or no history of surgical sterilization.
7. Safety laboratory assessments include hematology, chemistry, and coagulation. Coagulation will be performed only at Screening.
8. Blood for PK will be collected pre-dose (trough) and +5 minutes after dosing (peak).
9. Spirometry: DLCO on Screening only. On treatment days (Day 1, 7, 14), it will be done before dosing and post dose at 30 and 60 minutes.
10. Study staff will monitor subjects self-administrating study drug (most likely the morning dose) on Day 1, Day 7, and Day 14. On Day 1, the first dose of study drug will be administered at the investigational site and subjects will be monitored for at least 1 hour after dosing. Study drug is dispensed on Day 1 and Day 7.

14.2. Leicester Cough Questionnaire

This questionnaire is designed to assess the impact of cough on various aspects of your life. Read each question carefully and answer by CIRCLING the response that best applies to you. Please answer ALL questions, as honestly as you can.

1. In the last 2 weeks, have you had chest or stomach pains as a result of your cough?

1	2	3	4	5	6	7
All of the time	Most of the time	A good bit of the time	Some of the time	A little of the time	Hardly any of the time	None of the time

2. In the last 2 weeks, have you been bothered by sputum (phlegm) production when you cough?

1	2	3	4	5	6	7
Every time	Most times	Several times	Some times	Occasionally	Rarely	Never

3. In the last 2 weeks, have you been tired because of your cough?

1	2	3	4	5	6	7
All of the time	Most of the time	A good bit of the time	Some of the time	A little of the time	Hardly any of the time	None of the time

4. In the last 2 weeks, have you felt in control of your cough?

1	2	3	4	5	6	7
None of the time	Hardly any of the time	A little of the time	Some of the time	A good bit of the time	Most of the time	All of the time

5. How often during the last 2 weeks have you felt embarrassed by your coughing?

1	2	3	4	5	6	7
All of the time	Most of the time	A good bit of the time	Some of the time	A little of the time	Hardly any of the time	None of the time

6. In the last 2 weeks, my cough has made me feel anxious

1	2	3	4	5	6	7
All of the time	Most of the time	A good bit of the time	Some of the time	A little of the time	Hardly any of the time	None of the time

7. In the last 2 weeks, my cough has interfered with my job, or other daily tasks

1	2	3	4	5	6	7
All of the time	Most of the time	A good bit of the time	Some of the time	A little of the time	Hardly any of the time	None of the time

8. In the last 2 weeks, I felt that my cough interfered with the overall enjoyment of my life

1	2	3	4	5	6	7
All of the time	Most of the time	A good bit of the time	Some of the time	A little of the time	Hardly any of the time	None of the time

9. In the last 2 weeks, exposure to paints or fumes has made me cough

1	2	3	4	5	6	7
All of the time	Most of the time	A good bit of the time	Some of the time	A little of the time	Hardly any of the time	None of the time

10. In the last 2 weeks, has your cough disturbed your sleep?

1	2	3	4	5	6	7
All of the time	Most of the time	A good bit of the time	Some of the time	A little of the time	Hardly any of the time	None of the time

11. In the last 2 weeks, how many times a day have you had coughing bouts?

1	2	3	4	5	6	7
All of the time (continuously)	Most times during the day	Several times during the day	Some times during the day	Occasionally through the day	Rarely	None

12. In the last 2 weeks, my cough has made me feel frustrated

1	2	3	4	5	6	7
All of the time	Most of the time	A good bit of the time	Some of the time	A little of the time	Hardly any of the time	None of the time

13. In the last 2 weeks, my cough has made me feel fed up

1	2	3	4	5	6	7
All of the time	Most of the time	A good bit of the time	Some of the time	A little of the time	Hardly any of the time	None of the time

14. In the last 2 weeks, have you suffered from a hoarse voice as a result of your cough?

1	2	3	4	5	6	7
All of the time	Most of the time	A good bit of the time	Some of the time	A little of the time	Hardly any of the time	None of the time

15. In the last 2 weeks, have you had a lot of energy?

1	2	3	4	5	6	7
None of the time	Hardly any of the time	A little of the time	Some of the time	A good bit of the time	Most of the time	All of the time

16. In the last 2 weeks, have you worried that your cough may indicate serious illness?

1	2	3	4	5	6	7
All of the time	Most of the time	A good bit of the time	Some of the time	A little of the time	Hardly any of the time	None of the time

17. In the last 2 weeks, have you been concerned that other people think something is wrong with you, because of your cough?

1	2	3	4	5	6	7
All of the time	Most of the time	A good bit of the time	Some of the time	A little of the time	Hardly any of the time	None of the time

18. In the last 2 weeks, my cough has interrupted conversation or telephone calls

1	2	3	4	5	6	7
Every time	Most times	A good bit of the time	Some of the time	A little of the time	Hardly any of the time	None of the time

19. In the last 2 weeks, I feel that my cough has annoyed my partner, family or friends

1	2	3	4	5	6	7
Every time I cough	Most times when I cough	Several times when I cough	Some times when I cough	Occasionally when I cough	Rarely	Never

Thank you for completing this questionnaire.

14.3. Cough Visual Analogue Scale

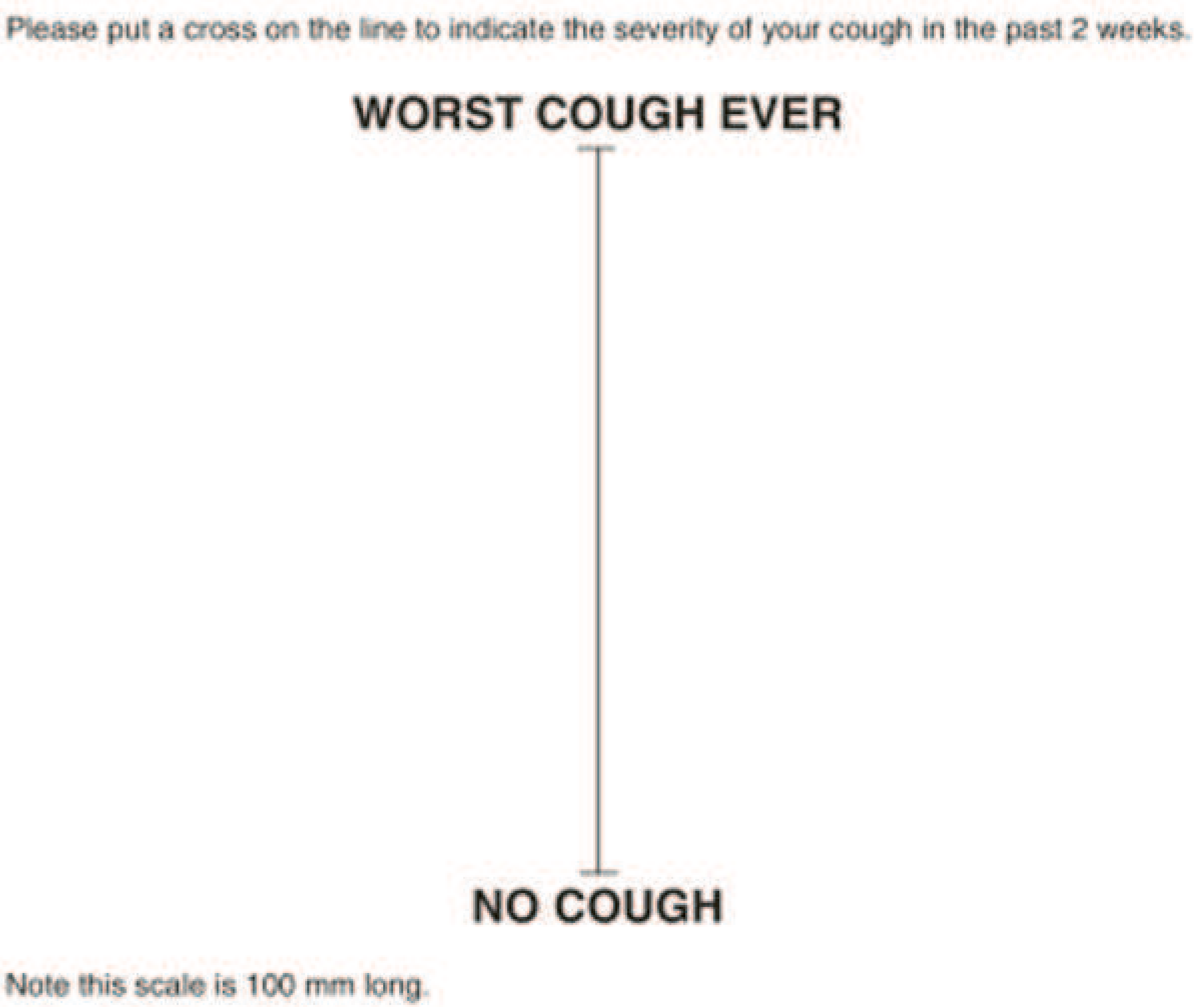

Please put a cross on the line to indicate the severity of your cough in the past 2 weeks.

WORST COUGH EVER

NO COUGH

Note this scale is 100 mm long.

DocuSign

Certificate Of Completion

Envelope Id: 55C9ECD7E1DD44A38456B9DEE6502240

Status: Completed

Subject: Complete with Docusign: LTI LTI031002 SAP - V2.0.docx

Source Envelope:

Document Pages: 30	Signatures: 2	Envelope Originator:
Certificate Pages: 3	Initials: 0	Katie Pottorff
AutoNav: Enabled		929 N Front St
EnvelopeId Stamping: Disabled		Wilmington, NC 28401
Time Zone: (UTC) Monrovia, Reykjavik		Katie.Pottorff@ppd.com
		IP Address: 208.127.228.158

Record Tracking

Status: Original<br>17 October 2024 | 15:05	Holder: Katie Pottorff<br>Katie.Pottorff@ppd.com	Location: DocuSign

Signer Events | Signature | Timestamp

Signer Events	Signature	Timestamp
Mikki Behnke<br>mbehnke@lungtx.com<br>Security Level: Email, Account Authentication (Required)	Mikki Behnke<br><br>Signature Adoption: Pre-selected Style<br>Signature ID:<br>7B3A3A80-1DEE-4B96-88DF-9FC97DD6D19C<br>Using IP Address: 174.22.239.59<br><br>With Signing Authentication via DocuSign password<br>With Signing Reasons (on each tab):<br>I approve this document	Sent: 17 October 2024 | 15:13<br>Viewed: 17 October 2024 | 17:54<br>Signed: 17 October 2024 | 17:56
**Electronic Record and Signature Disclosure:**<br>Accepted: 19 July 2023 | 15:05<br>ID: 43a759fc-8a24-4d43-88e6-089725d35c89		
Ichraq Latar<br>Ichraq.Latar@ppd.com<br>Security Level: Email, Account Authentication (Required)	Ichraq Latar<br><br>Signature Adoption: Pre-selected Style<br>Signature ID:<br>7ED091F4-04EE-4122-8499-8939BB80C4B7<br>Using IP Address: 15.188.233.136<br><br>With Signing Authentication via DocuSign password<br>With Signing Reasons (on each tab):<br>I approve this document	Sent: 17 October 2024 | 17:56<br>Viewed: 21 October 2024 | 13:23<br>Signed: 21 October 2024 | 13:24
**Electronic Record and Signature Disclosure:**<br>Not Offered via DocuSign		

In Person Signer Events	Signature	Timestamp

Editor Delivery Events	Status	Timestamp

Agent Delivery Events	Status	Timestamp

Intermediary Delivery Events	Status	Timestamp

Certified Delivery Events	Status	Timestamp

Carbon Copy Events	Status	Timestamp

Witness Events	Signature	Timestamp

Notary Events	Signature	Timestamp

Envelope Summary Events	Status	Timestamps
Envelope Sent	Hashed/Encrypted	17 October 2024 | 15:13
Certified Delivered	Security Checked	21 October 2024 | 13:23
Signing Complete	Security Checked	21 October 2024 | 13:24
Completed	Security Checked	21 October 2024 | 13:24

Payment Events	Status	Timestamps

Electronic Record and Signature Disclosure

PPD has established a corporate policy regarding the appropriate use of electronic records and electronic signatures, POL-00392, Appropriate Use of Electronic Records and Electronic Signatures

Supplementary Note 3. CONSORT 2010 Checklist

CONSORT 2010 checklist of information to include when reporting a randomised trial*

Section/Topic	Item No	Checklist item	Reported on page No
**Title and abstract**			
	1a	Identification as a randomised trial in the title	Title Page
	1b	Structured summary of trial design, methods, results, and conclusions (for specific guidance see CONSORT for abstracts)	1
**Introduction**			
Background and objectives	2a	Scientific background and explanation of rationale	2, 3
	2b	Specific objectives or hypotheses	1, 3, 13
**Methods**			
Trial design	3a	Description of trial design (such as parallel, factorial) including allocation ratio	13
	3b	Important changes to methods after trial commencement (such as eligibility criteria), with reasons	n/a
Participants	4a	Eligibility criteria for participants	13, 14
	4b	Settings and locations where the data were collected	13
Interventions	5	The interventions for each group with sufficient details to allow replication, including how and when they were actually administered	15, Fig 1
Outcomes	6a	Completely defined pre-specified primary and secondary outcome measures, including how and when they were assessed	13, 15-18
	6b	Any changes to trial outcomes after the trial commenced, with reasons	n/a
Sample size	7a	How sample size was determined	18
	7b	When applicable, explanation of any interim analyses and stopping guidelines	n/a
Randomisation:			
Sequence generation	8a	Method used to generate the random allocation sequence	15
	8b	Type of randomisation; details of any restriction (such as blocking and block size)	15
Allocation concealment mechanism	9	Mechanism used to implement the random allocation sequence (such as sequentially numbered containers), describing any steps taken to conceal the sequence until interventions were assigned	15
Implementation	10	Who generated the random allocation sequence, who enrolled participants, and who assigned participants to interventions	15
Blinding	11a	If done, who was blinded after assignment to interventions (for example, participants, care providers, those	15

		assessing outcomes) and how	
	11b	If relevant, description of the similarity of interventions	n/a
Statistical methods	12a	Statistical methods used to compare groups for primary and secondary outcomes	18
	12b	Methods for additional analyses, such as subgroup analyses and adjusted analyses	19-20
**Results**			
Participant flow (a diagram is strongly recommended)	13a	For each group, the numbers of participants who were randomly assigned, received intended treatment, and were analysed for the primary outcome	3, Figure1
	13b	For each group, losses and exclusions after randomisation, together with reasons	3, Figure1
Recruitment	14a	Dates defining the periods of recruitment and follow-up	3
	14b	Why the trial ended or was stopped	n/a
Baseline data	15	A table showing baseline demographic and clinical characteristics for each group	Table 1
Numbers analysed	16	For each group, number of participants (denominator) included in each analysis and whether the analysis was by original assigned groups	Table 1
Outcomes and estimation	17a	For each primary and secondary outcome, results for each group, and the estimated effect size and its precision (such as 95% confidence interval)	4, 5 ,Tables 2, 3
	17b	For binary outcomes, presentation of both absolute and relative effect sizes is recommended	n/a
Ancillary analyses	18	Results of any other analyses performed, including subgroup analyses and adjusted analyses, distinguishing pre-specified from exploratory	5, 6 Table 4
Harms	19	All important harms or unintended effects in each group (for specific guidance see CONSORT for harms)	4, 5
**Discussion**			
Limitations	20	Trial limitations, addressing sources of potential bias, imprecision, and, if relevant, multiplicity of analyses	11,12
Generalisability	21	Generalisability (external validity, applicability) of the trial findings	9, 12
Interpretation	22	Interpretation consistent with results, balancing benefits and harms, and considering other relevant evidence	6-12
**Other information**			
Registration	23	Registration number and name of trial registry	1,13
Protocol	24	Where the full trial protocol can be accessed, if available	13, 21
Funding	25	Sources of funding and other support (such as supply of drugs), role of funders	1, 25

*We strongly recommend reading this statement in conjunction with the CONSORT 2010 Explanation and Elaboration for important clarifications on all the items. If relevant, we also recommend reading CONSORT extensions for cluster randomised trials, non-inferiority and equivalence trials, non-pharmacological treatments, herbal interventions, and pragmatic trials. Additional extensions are forthcoming: for those and for up to date references relevant to this checklist, see www.consort-statement.org.
