# Supplementary material for: Inhaled LTI-03 for idiopathic pulmonary fibrosis: a randomized dose escalation study
Source: Nat Commun. 2026 Jul 31;17:7620. doi: 10.1038/s41467-026-75291-3 (PMC13427755; doi:10.1038/s41467-026-75291-3)
Supplement: Supplementary file 2 — Reporting Summary [file 41467_2026_75291_MOESM2_ESM.pdf]

nature portfolio

Corresponding author(s): ANDREAS GUENTHER MD, PhD

Last updated by author(s): 06/02/2026

Reporting Summary

Nature Portfolio wishes to improve the reproducibility of the work that we publish. This form provides structure for consistency and transparency in reporting. For further information on Nature Portfolio policies, see our Editorial Policies and the Editorial Policy Checklist.

Statistics

For all statistical analyses, confirm that the following items are present in the figure legend, table legend, main text, or Methods section.

n/a	Confirmed	
☐	☒	The exact sample size (n) for each experimental group/condition, given as a discrete number and unit of measurement
☐	☒	A statement on whether measurements were taken from distinct samples or whether the same sample was measured repeatedly
☐	☒	The statistical test(s) used AND whether they are one- or two-sided<br>*Only common tests should be described solely by name; describe more complex techniques in the Methods section.*
☒	☐	A description of all covariates tested
☐	☒	A description of any assumptions or corrections, such as tests of normality and adjustment for multiple comparisons
☐	☒	A full description of the statistical parameters including central tendency (e.g. means) or other basic estimates (e.g. regression coefficient) AND variation (e.g. standard deviation) or associated estimates of uncertainty (e.g. confidence intervals)
☐	☒	For null hypothesis testing, the test statistic (e.g. F, t, r) with confidence intervals, effect sizes, degrees of freedom and P value noted<br>*Give P values as exact values whenever suitable.*
☒	☐	For Bayesian analysis, information on the choice of priors and Markov chain Monte Carlo settings
☒	☐	For hierarchical and complex designs, identification of the appropriate level for tests and full reporting of outcomes
☐	☒	Estimates of effect sizes (e.g. Cohen's d, Pearson's r), indicating how they were calculated

Our web collection on statistics for biologists contains articles on many of the points above.

Software and code

Policy information about availability of computer code

Data collection	Clinical data were prospectively collected by clinical sites using a validated electronic data capture (EDC) system.
Data analysis	Clinical data analyses was performed using SAS statistical software (Version 9.4). Biomarker analyses were performed using GraphPAD Prism (Version 10.0).

For manuscripts utilizing custom algorithms or software that are central to the research but not yet described in published literature, software must be made available to editors and reviewers. We strongly encourage code deposition in a community repository (e.g. GitHub). See the Nature Portfolio guidelines for submitting code & software for further information.

Data

Policy information about availability of data

All manuscripts must include a data availability statement. This statement should provide the following information, where applicable:

- Accession codes, unique identifiers, or web links for publicly available datasets
- A description of any restrictions on data availability
- For clinical datasets or third party data, please ensure that the statement adheres to our policy

De-identified individual participant data for the analyses reported in this article are provided in the Source Data file. Additional de-identified clinical study data are unavailable due to ethical, legal, and proprietary restrictions protecting participant confidentiality. The full study protocol is provided as Supplementary Note 1 and

the statistical analysis plan is provided as Supplementary Note 2.

Research involving human participants, their data, or biological material

Policy information about studies with human participants or human data. See also policy information about sex, gender (identity/presentation), and sexual orientation and race, ethnicity and racism.

Reporting on sex and gender	The sex of participants was self-reported (as male or female) for demographic purposes after Informed Consent was obtained. Gender identity was not collected. Specific written informed consent for publication of participant-level data were was not obtained, therefore only aggregate data for sex is provided in Table 1. Results are presented for the overall study population only; no formal subgroup comparisons by sex were performed. The study enrolled 20 male and 4 female participants. Sex was not considered in the design of this study due to the small sample size (N=24).
Reporting on race, ethnicity, or other socially relevant groupings	Data on participant race and ethnicity were self-reported, and were collected for demographic purposes after Informed Consent was obtained. Results are presented for the overall study population only; no formal subgroup comparisons by race or ethnicity were performed. All participants were While/Caucasian and none were of Hispanic ethnicity.
Population characteristics	Eligible patients were male or female, ≥ 40 years of age willing and able to provide written informed consent. Participants were required to have a diagnosis of Idiopathic Pulmonary Fibrosis per ATS/ERS/JRS/ALAT guidelines made within 3 years of enrollment, with Forced Vital Capacity (FVC) percent predicted ≥ 40%, diffusion capacity of the lungs for carbon monoxide (DLCO) percent predicted between 30% and 80%, and Forced Expiratory Volume in 1 second (FEV_1)/FVC ≥ 0.7. Participant disposition included 24 participants (20 male, 4 female), with a median age of 68.1 and 71.7 years in th active and placebo groups, respectively.
Recruitment	Participants were recruited at 6 clinical sites in the United States, United Kingdom, Germany, and Australia beginning 22 June 2023. The first participant was enrolled on 06 July 2023, and the last participant completed all outcome assessments on and 01 October 2024. Recruitment was not stratified by sex or gender. Participants were screened according to protocol-defined eligibility criteria. Eligible participants who provided written informed consent were enrolled/randomized. Participants were compensated for their time and travel expenses according to local IRB/ethics committee-approved guidelines and standard clinical trial practices. Selection and self-selection biases may have occurred because participants had to meet eligibility criteria and be willing and able to complete study procedures. Together with the small sample size of 24 participants, these factors may limit generalization but are not expected to invalidate within-study comparisons based on protocol-defined assessments.
Ethics oversight	The study protocol and informed consent form were approved by the Institutional Review Board or Independent Ethics Committee for each site: South Central – Oxford A Research Ethics Committee for the Royal Brompton and Harefield Hospitals and the Royal Infirmary of Edinburgh (UK); Bellberry Human Research Ethics Committee for Launceston General Hospital (Australia); WCG IRB for University of Alabama at Birmingham (USA); Advarra , Inc. for Cedars-Sinai Medical Center (USA); and the Ethics Committee of the Faculty of Medicine for Justus-Liebig-University Giessen (Germany).

Note that full information on the approval of the study protocol must also be provided in the manuscript.

Field-specific reporting

Please select the one below that is the best fit for your research. If you are not sure, read the appropriate sections before making your selection.

☒ Life sciences ☐ Behavioural & social sciences ☐ Ecological, evolutionary & environmental sciences

For a reference copy of the document with all sections, see nature.com/documents/nr-reporting-summary-flat.pdf

Life sciences study design

All studies must disclose on these points even when the disclosure is negative.

Sample size	A total of 24 participants were enrolled: 18 received LTI-03 and 6 received placebo). The sample size of 18 LTI-03 participants provided a 60% chance of detecting an AE with a true incidence rate of 5% and an 85% chance of detecting a more common AE with a true incidence rate of 10%. Placebo participants in each cohort were pooled for safety and biomarker analyses.
Data exclusions	For biomarker analyses, occasional samples were excluded due to missed collection or values below the lower limit of quantification (BLQ). These exclusions are explicitly reported in the relevant Data Tables and Methods. No data were excluded from the primary safety analysis.
Replication	The findings were consistent across study participants and cohorts. Key measurements were obtained using validated methods, and results were robust to standard data quality assessments without evidence of undue influence from outliers. Given the sample size typical of an early-phase clinical trial, independent replication was not performed.
Randomization	Eligible participants were randomized 3:1 to LTI-03 or placebo into 2 sequential cohorts (n=12 per cohort). The 5 mg/day (low-dose) cohort received 2.5 mg LTI-03 or placebo BID and the 10 mg/day (high dose) cohort received 5 mg BID LTI-03 or placebo. Randomization was performed by interactive response system.
Blinding	Participants, study staff, and sponsor personnel were blinded to treatment assignment throughout the study.

Reporting for specific materials, systems and methods

We require information from authors about some types of materials, experimental systems and methods used in many studies. Here, indicate whether each material, system or method listed is relevant to your study. If you are not sure if a list item applies to your research, read the appropriate section before selecting a response.

Materials & experimental systems

n/a	Involved in the study
☐	☒ Antibodies
☒	☐ Eukaryotic cell lines
☒	☐ Palaeontology and archaeology
☒	☐ Animals and other organisms
☐	☒ Clinical data
☒	☐ Dual use research of concern
☒	☐ Plants

Methods

n/a	Involved in the study
☒	☐ ChIP-seq
☒	☐ Flow cytometry
☒	☐ MRI-based neuroimaging

Antibodies

Antibodies used	Biomarker analyses. SP-D: MSD #K1519XR-2; AKT/pAKT: MSD #K15100D-1; COL1A1: Abcam #ab210966<br>CXCL7: LS Bio #LS-F4967-1; Galectin-7: R&D Systems #DY1339; IL-11: R&D Systems #DY218<br>TSLP: R&D Systems #DY1398.
Validation	All biomarker assays were performed according to the manufacturers' instructions after optimization for IPF matrices. No additional in-house validation of individual antibodies was performed.

Clinical data

Policy information about clinical studies

All manuscripts should comply with the ICMJE guidelines for publication of clinical research and a completed CONSORT checklist must be included with all submissions.

Clinical trial registration	Clinicaltrials.gov NCT05954988
Study protocol	The full study protocol is provided as Supplementary Note 1.
Data collection	Data was collected from 22 June 2023 to 01 October 2024. Data was collected during Screening and on Days 1, 7, 14 (treatment), and Day 21 (follow-up).
Outcomes	Primary outcome: incidence of treatment-emergent adverse events (TEAEs).<br>Exploratory outcomes: pharmacokinetics and change from baseline in disease-related biomarkers.

Plants

Seed stocks	*Report on the source of all seed stocks or other plant material used. If applicable, state the seed stock centre and catalogue number. If plant specimens were collected from the field, describe the collection location, date and sampling procedures.*
Novel plant genotypes	*Describe the methods by which all novel plant genotypes were produced. This includes those generated by transgenic approaches, gene editing, chemical/radiation-based mutagenesis and hybridization. For transgenic lines, describe the transformation method, the number of independent lines analyzed and the generation upon which experiments were performed. For gene-edited lines, describe the editor used, the endogenous sequence targeted for editing, the targeting guide RNA sequence (if applicable) and how the editor was applied.*
Authentication	*Describe any authentication procedures for each seed stock used or novel genotype generated. Describe any experiments used to assess the effect of a mutation and, where applicable, how potential secondary effects (e.g. second site T-DNA insertions, mosiacism, off-target gene editing) were examined.*
