# Supplementary material for: Inhaled LTI-03 for idiopathic pulmonary fibrosis: a randomized dose escalation study
Source: Nat Commun. 2026 Jul 31;17:7620. doi: 10.1038/s41467-026-75291-3 (PMC13427755; doi:10.1038/s41467-026-75291-3)
Supplement: Supplementary file 3 — Transparent Peer Review file [file 41467_2026_75291_MOESM3_ESM.pdf]

nature portfolio

Peer Review File

Inhaled LTI-03 for Idiopathic Pulmonary Fibrosis: A Randomized Dose Escalation Study

Corresponding Author: Professor Andreas Günther

This file contains all reviewer reports in order by version, followed by all author rebuttals in order by version.

Version 0:

Reviewer comments:

Reviewer #1

(Remarks to the Author)
This article presents the results of a randomized dose escalation study (2 doses) of an inhaled therapy for the treatment of IPF. The novelty of this work is in the therapeutic approach: use of an inhaled formulation and therapeutic mechanism aiming to protect the alveolar epithelium. Though the numbers are small, the results of the analysis of deep bronchial brushing samples support a pharmacodynamic effect at the higher dose. Overall however there is insufficient level of detail provided for the primary safety endpoint.

-The abstract states "there was no airway obstruction by spirometry or symptoms." The use of symptoms here is vague. What symptoms are being referred to here? Cough?
-FEV1 and FVC data over the 14 days should be reported as these are important safety measurements - suggest both numerical summary in the text and as a figure with values over time
-More detailed reporting of AE's is needed - it would be helpful to detail the etiology of all AEs
-More discussion of the PK results would be helpful - are these results c/w what would be expected that there should be no systemic absorption with an inhaled delivery formulation
-This study was done in participants not on SOC IPF treatment and the field is moving toward combination therapy approaches - consider adding mention of this in the discussion

Reviewer #2

(Remarks to the Author)
Philip et al. conducted a dose-escalation trial to assess the potential of LTI-03 in patients with IPF. This is a Phase Ib, placebo-controlled study following a standard trial design. I do not have any major concerns; however, the following points may help improve the reporting:

1,The results in the abstract are presented as text only, without quantitative data or comparisons. Including key numerical results would improve clarity.
2,Please clarify why 90% confidence intervals were used for reporting the results.
3,Could you provide a reference for Cliff's delta and explain how the interpretation thresholds were determined?
4,Please revise Figure 1 to align with CONSORT guidelines.

Reviewer #3

(Remarks to the Author)
General comments:
The authors report the results of a phase 1b study of a novel inhaled agent for IPF. The authors provide BAL biomarker evidence of drug target engagement and a rationale for potential benefit in IPF patients. This study lays the foundation for a future phase 2 clinical trial of this novel agent. The conclusions of the paper are justified by the findings. There are no obvious flaws in the data analysis, interpretation and conclusions. The methodologies employed are sound with sufficient detail provided for this work to be reproduced. This is a very well-written paper and we don't have any major comments or concerns.

Minor comments
1. The instructions on the journal website state “The main text of an Article should begin with a section headed Introduction of referenced text that expands on the background of the work (some overlap with the abstract is acceptable), followed by sections headed Results, Discussion (if appropriate) and Methods (if appropriate).” In our opinion, the article would be an easier read and more appropriate to this type of manuscript if the methods were sequenced before the results.
2. How many patients were screened and didn’t qualify?
3. “. No prior use of any approved or investigational antifibrotic drug was reported for any participant.” Would that be “prior” and “current”?
4. I suspect the sample size was too small, but was there a dose response in any of the BAL biomarkers?
5. Although 2 weeks is a very short period of time in the context of clinical change in IPF, it might be informative to see spaghetti plots of FVC change over this time period for all the subjects.

Reviewer #4

(Remarks to the Author)
I co-reviewed this manuscript with one of the reviewers who provided the listed reports. This is part of the Nature Communications initiative to facilitate training in peer review and to provide appropriate recognition for Early Career Researchers who co-review manuscripts.

Version 1:

Reviewer comments:

Reviewer #1

(Remarks to the Author)
My comments have been addressed.

Reviewer #2

(Remarks to the Author)
thanks for providing the response. I have no further comments

Reviewer #3

(Remarks to the Author)
Thank you for addressing all the issues raised.
I have no additional suggestions.

Reviewer #4

(Remarks to the Author)
I co-reviewed this manuscript with one of the reviewers who provided the listed reports. This is part of the Nature Communications initiative to facilitate training in peer review and to provide appropriate recognition for Early Career Researchers who co-review manuscripts.

Open Access This Peer Review File is licensed under a Creative Commons Attribution 4.0 International License, which permits use, sharing, adaptation, distribution and reproduction in any medium or format, as long as you give appropriate credit to the original author(s) and the source, provide a link to the Creative Commons license, and indicate if changes were made.
In cases where reviewers are anonymous, credit should be given to 'Anonymous Referee' and the source.
The images or other third party material in this Peer Review File are included in the article's Creative Commons license, unless indicated otherwise in a credit line to the material. If material is not included in the article's Creative Commons license and your intended use is not permitted by statutory regulation or exceeds the permitted use, you will need to obtain permission directly from the copyright holder.
To view a copy of this license, visit https://creativecommons.org/licenses/by/4.0/

Point-by-Point Response to Reviewers
Manuscript Title: A Randomized Dose Escalation Study of Inhaled LTI-03 for Idiopathic Pulmonary Fibrosis
Manuscript ID: NCOMMS-26-012740
Corresponding Author: Andreas Günther, MD. PhD.
April 22, 2026

Reviewer #1 (Remarks to the Author)
This article presents the results of a randomized dose escalation study (2 doses) of an inhaled therapy for the treatment of IPF. The novelty of this work is in the therapeutic approach: use of an inhaled formulation and therapeutic mechanism aiming to protect the alveolar epithelium. Though the numbers are small, the results of the analysis of deep bronchial brushing samples support a pharmacodynamic effect at the higher dose. Overall however there is insufficient level of detail provided for the primary safety endpoint.

1.The abstract states "there was no airway obstruction by spirometry or symptoms" The use of symptoms here is vague. What symptoms are being referred to here? Cough?

We thank the reviewer for their thoughtful feedback. Airway obstruction symptoms manifest most commonly as wheezing, dyspnea, and chest tightness. We have added a description of these symptoms to the Abstract (pg 1), Safety and Tolerability results (pg 4), and Discussion pg 7. ’

2. FEV1 and FVC data over the 14 days should be reported as these are important safety measurements.

We have added Table 3, which summarizes mean (SD) change from baseline in FEV_1 and FVC for each timepoint, from Baseline through follow-up. We have also provided the by-patient data for change in FEV_1 and FVC over time as Supplemental Fig. 1.

3. *More detailed reporting of AE's is needed - it would be helpful to detail the etiology of all AEs*

Thank you. We have provided a list of all TEAEs reported in the study as a new Supplemental Table 1. This table includes a listing of all TEAEs and all TEAEs related to study drug. Other than cough and rhinorrhea, the remaining TEAEs reported across all body systems were reported in only 1 participant each. The table also notes which TEAEs were serious (n=1, post-procedural fever), or Grade 2 severity (n=2 cough; n=1 post-procedural fever).

We have also clarified which AEs were Grade 2 in severity in the text (pg 4).

‘Grade 2 TEAEs were reported by 3 participants (16.7%) receiving LTI-03, including 2 cases of cough and 1 case of post-procedural fever.’

Additionally, please refer to the discussion, pg. 7, where additional details regarding the cough are discussed.

‘The majority of cough TEAEs were mild in severity except for 2 study drug-related TEAEs of Grade 2 severity, each resolving on the same day: one occurred as a single episode and the other occurred intermittently. All other study drug-related TEAEs of cough were intermittent in frequency and Grade 1 in severity, occurring intermittently after study drug administration.’

4. More discussion of the PK results would be helpful - are these results c/w what would be expected that there should be no systemic absorption with an inhaled delivery formulation.

We appreciate the helpful request for additional clarity and context. We have added language to the discussion section (pg.7) as follows:

‘Undetectable plasma levels of LTI-03, a hydrophobic peptide, are consistent with the PK behavior of other inhaled hydrophobic compounds, such as inhaled cyclosporine formulations, which exhibit rapid and sustained lung absorption with minimal systemic exposure 32. Taken together, these data support lung-targeted delivery of LTI 03 with minimal systemic exposure.’

5.*This study was done in participants not on SOC IPF treatment and the field is moving toward combination therapy approaches - consider adding mention of this in the discussion*

We appreciate the recommendation and have added the following to the discussion. (pg .11).

‘While none of the participants were receiving antifibrotic therapy in this study, combination therapies are emerging as standard of care. Larger studies of longer treatment durations in patients with IPF receiving antifibrotic therapy will be required to confirm and expand upon the findings of this first-in-patient study.’

Additionally, we clarified that “no prior use of antifibrotics” was defined as no use within the 2 months prior to screening, in accordance with the protocol. These edits may be found in Results (pg. 4) and Discussion (pg.6).

Reviewer #2 (Remarks to the Author)
Philip et al. conducted a dose-escalation trial to assess the potential of LTI-03 in patients with IPF. This is a Phase Ib, placebo-controlled study following a standard trial design. I do not have any major concerns; however, the following points may help improve the reporting:

1. The results in the abstract are presented as text only, without quantitative data or comparisons.

We thank the reviewer for these constructive suggestions that improve clarity and transparency. Unfortunately, since we must accommodate additional mandatory edits, we do not have the word count to list out quantitative data in the abstract.

However, based on the reviewer's suggestion, we have added p-values to the abstract (pg. 1) for the comparative biomarker data.

2. *Please clarify why 90% confidence intervals were used for reporting the results.*

We are happy to provide clarity. We first want to highlight existing language in the 'Statistical Analyses' section (pg. 19):

"The Hodges-Lehmann estimator was used for the median difference between groups (treatment minus placebo), with approximate 90% confidence intervals (asymmetric, exact level varied slightly with small n)."

We chose 90% (rather than the conventional 95%) CIs for two closely related reasons that are directly tied to the study design:

(a) the biomarker analyses were exploratory and based on one-tailed Mann-Whitney U tests; and

(b) the sample sizes were very small (n = 5–9 per group).

The one-tailed approach itself was chosen because the biomarkers were pre-selected from nonclinical and translational experiments (precision-cut lung slices, alveolar organoids, and bleomycin-challenged aged mice) that consistently showed directional *reduction* of these analytes following LTI-03 treatment. We hope this is adequately explained with existing language in the Statistical Analysis section (pg. 18).

'Since exploratory biomarkers were selected based on data generated by prior translational experiments which demonstrated reduction following LTI-03 treatment in various models, one-tailed statistical analyses were performed.'

For the Hodges-Lehmann estimator in small, non-normally distributed datasets, the exact confidence interval is often asymmetric; using a 90% level maintains appropriate power and produces informative (non-overly wide) intervals when n < 10. This practice is standard for early-phase clinical studies with pre-specified directional hypotheses. The full analyses details are provided for reproducibility.

3. *Could you provide a reference for Cliff's delta and explain how the interpretation thresholds were determined?*

Cliff's delta (δ) was used to provide an effect-size measurement, accompanying the one-tailed Mann-Whitney U test. We have clarified thresholds are effect-size thresholds in the manuscript (pg 19):

*"Cliff's delta was calculated as effect size using the formula $\delta = (2U / (n1 \times n2)) - 1$, with thresholds: $|\delta| < 0.3$ small **effect**, 0.3–0.5 medium **effect**, > 0.5 large **effect**. The sign was flipped to negative when treatment changes were lower than placebo (indicating reductions)."*

Interpretation of the thresholds is as follows (and fully described in Romano et al, 2006; see below):The cut-offs (|δ| < 0.3 = small, 0.3–0.5 = medium, > 0.5 = large) are the original, widely accepted conventions established by Cliff and are the default thresholds implemented in GraphPad Prism v10.0 (the software used for all biomarker analyses). Because we report both the signed Cliff's delta values and the corresponding 90% CIs for the median differences in Table 4 and Supplementary Figure 2, readers can fully interpret the magnitude of the LTI-03 effect on each biomarker (e.g., δ = 0.72 for COL1A1 at 10 mg/day indicates a large effect).

We have also added the key reference for this in the bibliography:

Romano, J., Kromrey, J. D., Coraggio, J. & Skowronek, J. Appropriate statistics for ordinal level data: Should we really be using t-test and Cohen's d for evaluating group differences on the NSSE and other surveys? Presented at the Annual meeting of the Florida Association of Institutional Research. 1–3 February 2006, Cocoa Beach, Florida.

Finally, we added further context to the discussion (pg. 11)

"Although the sample size was limited, the reductions in profibrotic biomarkers were supported by medium-to-large effect sizes (Cliff's delta), reinforcing the biological relevance of these pharmacodynamic signals despite the short 14-day treatment period."

The reference and these clarifications are now highlighted in the revised manuscript We hope they provide the additional transparency requested by the reviewer.

4. Please revise Figure 1 to align with CONSORT guidelines

Thank you, this has been completed and provided as updated Figure 1.

Reviewer #3 (Remarks to the Author)

General comments: The authors report the results of a phase 1b study of a novel inhaled agent for IPF. The authors provide BAL biomarker evidence of drug target engagement and a rationale for potential benefit in IPF patients. This study lays the foundation for a future phase 2 clinical trial of this novel agent. The conclusions of the paper are justified by the findings. There are no obvious flaws in the data analysis, interpretation and conclusions. The methodologies employed are sound with sufficient detail provided for this work to be reproduced. This is a very well-written paper and we don't have any major comments or concerns. Minor comments:

1. The instructions on the journal website state "The main text of an Article should begin with a section headed Introduction of referenced text that expands on the background of the work (some overlap with the abstract is acceptable), followed by sections headed Results, Discussion (if appropriate) and Methods (if appropriate)." In our opinion, the article would be an easier read and more appropriate to this type of manuscript if the methods were sequenced before the results.

We appreciate the suggestion. However, the journal's submission guidelines dictate the flow of sections which this manuscript follows. We have retained the current structure to follow journal style.

2. How many patients were screened and didn't qualify?

Please refer to updated Figure 1, which now displays a formal CONSORT Diagram. That includes the total number of participants screened (N=26) and the number of screen failures (N=2). We have also added context for exclusion to the figure legend:

3. "No prior use of any approved or investigational antifibrotic drug was reported for any participant." Would that be "prior" and "current"?

Thank you for this clarification request. We have updated appropriate sections to clarify that that this was within 2 months of screening, or during the study. These edits may be found in the Results (pg. 4) and Discussion (pg.6).

4. I suspect the sample size was too small, but was there a dose response in any of the BAL biomarkers?

The reviewer's suspicion is correct. Given the small sample size (Phase 1b safety design), we did not perform formal dose-response testing. We note in the Discussion that greater reductions were generally observed with the 10 mg/day dose for several key biomarkers (e.g., SP-D, COL1A1, CXCL7, GAL-7).

5. Although 2 weeks is a very short period of time in the context of clinical change in IPF, it might be informative to see spaghetti plots of FVC change over this time period for all the subjects.

We have now included spaghetti plots for change from baseline in FVC and FEV_1 as part of the new Table 3 and new Supplemental Figure 1.

Reviewer #4 (Remarks to the Author):
I co-reviewed this manuscript with one of the reviewers who provided the listed reports. This is part of the Nature Communications initiative to facilitate training in peer review and to provide appropriate recognition for Early Career Researchers who co-review manuscripts.

We thank the reviewer for co-reviewing the manuscript and for their time.

We believe these revisions have substantially strengthened the manuscript and fully address the editorial and reviewer feedback. All source data files have been updated to reflect the new figures and analyses. We look forward to your continued consideration of this work for publication in Nature Communications.
